# Hypobaric hypoxia induces iron mobilization from liver and spleen and increases serum iron via activation of ghrelin/GHSR1a/MAPK signalling pathway in mice

**DOI:** 10.1038/s41598-023-47596-6

**Published:** 2023-11-20

**Authors:** Wanping Yang, Jiayan Li, Jianan Hu, Xiaoyu Yuan, Jie Ding, Hui Jiang, Guohua Wang, Qianqian Luo

**Affiliations:** 1 Department of Physiology and Hypoxic Biomedicine, Institute of Special Environmental Medicine, Nantong University, 9 Seyuan Road, Chongchuan District, Nantong, 226019 Jiangsu China; 2 Department of Emergency, Affiliated Hospital of Nantong University, 20 XiSi Road, Nantong, 226001 China

**Keywords:** Cell biology, Molecular biology, Physiology, Environmental sciences, Endocrinology, Gastroenterology, Medical research

## Abstract

Hypobaric hypoxia (HH) exposure affects appetite and serum iron levels in both humans and animals. Thus, whether appetite-regulating ghrelin is involved in iron regulation under HH needs to be elucidated. In vivo, C57BL/6J mice were placed in a hypobaric chamber to establish a 6000-m-high altitude exposure animal model. In vitro, mouse primary hepatocytes and peritoneal macrophages were exposed to hypoxia (1% O_2_) to examine the effects of ghrelin on iron-regulating proteins. HH obviously reduced the body weight of mice and significantly increased the levels of erythrocytes, and also significantly enhanced the levels of serum iron and plasma ghrelin. However, iron content in the liver and spleen was decreased, while ferroportin (Fpn) expression was increased. Moreover, ghrelin significantly induced Fpn and pERK expression in both hepatocytes and macrophages under hypoxia, which were reversed by pretreatment with growth hormone secretagogue receptor 1a (GHSR1a) antagonist or pERK inhibitor. Our findings indicated that HH leads to decreased appetite and insufficient dietary intake, which may negatively regulate the levels of ghrelin. Furthermore, GHSR1a/ERK signalling pathway is further activated to upregulate the expression of Fpn, and then promoting iron mobilization both in the liver/hepatocytes and spleen/macrophages in mice. Thus, these results revealed that ghrelin may be a potential iron regulatory hormone, and raised the possibility of ghrelin as a promising therapeutic target against iron disorders under HH.

## Introduction

Hypobaric hypoxia (HH) is the main cause of stress on the human body in high-altitude environments. The human body evolved and developed beneficial physiological defence mechanisms to adapt to HH conditions^1–3^, including inducing an appropriate increase in the number of red blood cells (RBCs) in the blood and thereby haemoglobin (HGB) and oxygen content^4^. However, HGB is the main component of RBCs, and approximately 70% of the iron in the human body exists in HGB^5^. The intricate network of iron metabolism within the human body predominantly encompasses the critical processes of absorption, storage, and subsequent re-utilization^6^. Hepcidin, a key regulator of iron homeostasis, plays a crucial role in the body’s response to hypoxia^7^. Under hypoxic conditions, the body increases erythropoiesis to enhance oxygen delivery to tissues, subsequently elevating the demand for iron^8^. Hepcidin expression is modulated in response to this increased iron demand to facilitate iron availability^8^. Specifically, hypoxia has been shown to suppress hepcidin expression, thereby promoting iron mobilization from stores and enhancing iron absorption from the intestine^9^. This intricate balance ensures adequate iron supply for erythropoiesis while preventing iron overload.

While the intestinal absorption of iron plays an integral role in the overall schema of iron metabolism, its contribution remains relatively minor in the grand spectrum of iron regulation^10^. Under hypoxic conditions, hypoxia-inducible factor-2α (HIF-2α) plays a pivotal role in upregulating the expression of genes involved in iron absorption, such as divalent metal transporter 1 (DMT1) and ferroportin (Fpn)^11^. This adaptation facilitates increased iron absorption to meet the elevated iron requirements during hypoxia. However, it is important to note that while these changes occur, the contribution of intestinal iron absorption to overall iron metabolism remains relatively modest compared to the iron recycled from aged erythrocytes by the liver and spleen^6^. The liver and spleen are considered iron storage and iron recycling organs that maintain body iron homeostasis because hepatocytes of the liver are the major cell type responsible for iron storage, and red pulp macrophage (RPM) is the main cell type responsible for iron recycling^12,13^. It was reported that iron used for daily HGB synthesis was mainly supplied by the spleen. RPMs are known to phagocytize and degrade RBCs that are senescent, aged, dead or opsonized, leading to increased iron release and recycling^14^. The balance of iron homeostasis depends on the expression levels and activities of iron carriers, iron transporters, and iron regulatory and storage proteins^13^. Transferrin receptor (TfR) is an important receptor that contributes to cellular iron uptake, and the function of ferritin light chains (Ft-L) is related to cellular iron storage^15^. Fpn functions in exporting iron into the circulation and is the only distinct cellular iron exporter known to date^16^. Investigations into the molecular mechanisms of cellular iron metabolism may promote the fields of iron physiology and pathophysiology under HH conditions.

In addition to the above abnormalities in iron metabolism^17^, loss of appetite and reduced food intake are also observed under HH^18^. Ghrelin, a 28-amino acid polypeptide secreted by P/D1 cells at the bottom of the stomach^19^, participates in the regulation of food intake and energy metabolism^20^. Serine-3 of ghrelin can be acylated by ghrelin O-acyltransferase (GOAT) with an eight-carbon fatty acid (octanoate)^21^. Acylated ghrelin exhibits its physiological functions via recognition and binding of the growth hormone secretagogue receptor (GHSR1a), leading to the activation of downstream signalling pathways. It was reported that ghrelin expression was enhanced both in humans and rats after high-altitude exposure or HH treatment^22,23^. Our previous studies also suggested that ghrelin mediates iron metabolism and especially iron export both in the spleen and liver after starvation in mice^24,25^. Other research reported that HH exposure affected appetite and serum iron levels in healthy humans^26^; however, the role of appetite-regulating ghrelin signalling in iron metabolism under HH remains to be elucidated^17,27^. Here, we hypothesize that HH-induced ghrelin release may take effect on regulating iron metabolism and intend to illustrate the potential mechanisms of the influence of HH on iron homeostasis.

## Materials and methods

### Animal ethics approval

All animal care and experimental protocols were carried out according to the Chinese Animal Management Rules of the Ministry of Health and were authorized by the Animal Ethics Committees of Nantong University research program protocol (#NTU-19-023). The study was carried out in accordance with ARRIVE guidelines.

### Chemicals and reagents

Unless otherwise stated, all chemicals and reagents used in this study were supplied by Thermo Fisher Scientific (Waltham, MA USA). Ghrelin and acylated-ghrelin ELIST kits (A-05118 and A-05317) were purchased from Cayman (Spi-bio, Bertin Pharma, MI, USA). Ghrelin was obtained from BioVision (4990-1000, CA, USA). U0126 (abs810003) was supplied by Absin (Shanghai, China). D-(lys-3)-GHRP-6 (DLG) was purchased from Selleck (136054-22-3, Houston, TX, USA).

### Animals and hypobaric hypoxia treatment

C57BL/6 male mice (8-weeks-old) were obtained from the animal experimental centre of Nantong University. All mice used were housed at an ambient temperature of 22 ± 2 °C with a relative humidity of 50% to 60%, alternating 12-h periods of light and dark, and free access to water and food. After one week of acclimatization, all animals were randomly assigned to different groups (n = 9 per group). A hypobaric hypoxia chamber was used to mimic the high-altitude exposure at an altitude of 6000 m. There was water and food in the chamber, and the mice in the HH group were placed in the chamber for different durations up to 7 days. The control group animals were maintained in a similar chamber under normobaric normoxia (NN) conditions for the same time. After HH exposure, the animals were removed from the chamber. The mice were then euthanized by asphyxiation using CO_2_, and all in vitro samples were collected within 30 min. Blood was collected for the detection of ghrelin and acylated ghrelin expression in the plasma, serum iron levels, unsaturated iron-binding capacity, total iron-binding capacity (TIBC) and transferrin (Tf) saturation in the serum as described previously^28^. The spleen was collected for iron staining, iron content determination and measurement of iron-regulating proteins as described previously^25^.

### Haematological indices

The blood of the mice was collected after different periods of HH exposure. RBC count, HGB content, haematocrit (HCT) and mean corpuscular haemoglobin (MCH) in blood were measured using a haematology analyser (Mindray, BC-2800, China) according to the manufacturer's instructions.

### Immunohistochemistry and semiquantitative analysis

As described previously^24,29^, liver and spleen tissues from mice were fixed in 4% formaldehyde and embedded in paraffin. Tissue sections (5 μm thick) were routinely passed through xylene, a graded alcohol series, and washed with PBS. After the antigens repairing and the blocking of endogenous catalase, the slice specimens were incubated with anti-ghrelin (1:250; ab104307, Abcam, USA) and Ft-L antibody (1:200, ab69090, Abcam, USA). The specific bindings were visualized with DAB detection Kit (Polymer) (Kit-0015, MXB Biotechnologies, China), and then lightly counterstained with hematoxylin. Five slices of each group were chosen for determining the distribution and expression of ghrelin and Ft-L under a DM4000B microscope (Leica, Germany). Five visual fields were selected randomly in each slice for semiquantitative analysis by ImageJ software (National Institutes of Health, Bethesda, MD, USA). Integrated density of different treatment groups was finally normalized to NN group. The analysis was done by an investigator blinded to experimental group.

### Enhanced Perls’ staining

To observe iron accumulation in paraffin-embedded liver and spleen sections, DAB-enhanced Perls’ staining was generated as described in our previous work^30^. According to the manufacturer’s instructions, liver tissue sections were washed with PBS and cultured in freshly prepared Perls’ solution (1% potassium ferricyanide in 0.1-M hydrochloric acid buffer). The slides were then immersed and stained with DAB. All slides were counterstained with haematoxylin and visualized under a DM4000B microscope (Leica, Germany). Data were collected from three fields of view per mice, and semi-quantitatively analyzed with ImageJ software. Quantitative results of Perls’ staining were finally normalized to NN group.

### Tissues iron measurements

Iron content was detected using the tissue iron measurement method as previous method^30^. Briefly, liver and spleen tissue (0.1 g) were obtained, and then, 1 mL of tissue digestive liquid (3 M hydrochloric acid and 0.61 M trichloroacetic acid) was added at 65 °C for 3 days to ensure complete tissue digestion. The volume of tissue digestion liquid was then fixed to 1.5 mL, and centrifuged at 10,000 g for 10 min. The supernatant was collected for analysis. The 96-well plate was further used for detection, and 200 μL iron developing working solution was added to each well. Then, 10 μL ddH_2_O, 10 μL tissue digestion solution or 10 μL iron standard solution (500 µg/dL) was added to different wells. Finally, 10 μL of sample was added to the sample well, fully mixed, and incubated at 37 °C for 10 min. Absorbance was measured at 535 nm using a Synergy 2 multimode microplate reader (Biotek Instruments, USA). The quantification of iron content (μg/g Tissue wet weight) was calculated as: OD/tissue weight × (1.5–0.25 × tissue weight) × (1/iron standard 500 OD × 4.77).

### Isolation and culture of mouse hepatocytes

Primary hepatocytes were obtained according to methods described in our previous study^24^. In brief, male C57BL/6J mice were used to obtain hepatocytes by a two-step collagenase IV perfusion procedure. The livers were first perfused with pre-perfusion buffer at 37 °C for 15 min, followed by enzyme buffer containing 0.1 mg/mL collagenase IV perfusion until the liver capsule was incised at 37 °C. Then, the liver was isolated and digested into single cells in dishes with 10% foetal bovine serum (FBS) 1640 medium. The suspension was filtered and centrifuged to collect all of the cells. To obtain purified hepatocytes, the whole cells were subsequently centrifuged in Percoll solution. Finally, the collected hepatocytes were seeded into plates (~ 2 × 10^6^ cells/well, six-well plates) and cultured in 1640 medium with 10% FBS at 37 °C in a 5% CO2 incubator.

### Primary peritoneal macrophage culture

Primary peritoneal macrophages were harvested and cultured as previously described^25^. In brief, C57BL/6 male mice (8-weeks old) were injected with 1 mL of 3% thioglycollate medium (i.p.). Three days later, the animals were euthanized, and their peritoneal macrophages were obtained by peritoneal lavage with sterile PBS. The cells were grown in DMEM supplemented with 10% FBS in the presence of antibiotics (penicillin 100 U/ml, streptomycin 100 µg/ml) and cultured in a 5% CO_2_ incubator (Thermo, Forma 311, USA) at 37 °C. Peritoneal macrophages were seeded onto six-well plates (~ 2 × 10^6^ cells/well) for the following experiments.

### Cell treatment

The dosages and time points of ghrelin, GHSR1a antagonist, and U0126 administrations for hepatocytes and macrophages were described in our previous research^24,25^. To determine the effects of the GHSR1a antagonist and pErK inhibitor, cultured hepatocytes or macrophages were pretreated with 100 nM DLG or 10 μM U0126 for 1 h, followed by 10^−7^ M ghrelin treatment for another 12 h under 1% O_2_. Then, the cells were washed with PBS and lysed to obtain total proteins for further analysis.

### Western blot

Proteins were extracted from spleen tissue or primary peritoneal macrophages, washed with PBS, homogenized with RIPA lysis buffer (Beyotime, Haimen, China) and then sonicated using Selecta Sonopuls (Jinxin, Shanghai, China)^20^. Protein concentrations were determined by a BCA assay kit. Western blot samples containing 30 μg of protein were loaded and run in each well of 10% SDS–PAGE gels under reducing conditions and subsequently transferred to a pure PVDF membrane (Millipore, Merk, Germany), which was then blocked with 5% nonfat milk and incubated with different primary antibodies overnight at 4 °C. The primary antibodies used in this study included anti-TfR (1:1,000, 13-6800, Life Technologies, USA), anti-Fpn (1:1000, NBP1-21502, Novus Biologicals, USA), anti-Ft-L (1:1,000, ab69090, Abcam，USA), anti-pErK against 42/44 (1:1000, 1240s, Cell siganling technology, USA), anti-ErK (1:1000, 4695, Cell siganling technology, USA), and anti-ghrelin (1:500, ab129383, Abcam, USA). The blotted PVDF membranes were washed with PBS and incubated with goat anti-rabbit (926-32211, LI-COR, USA) or anti-mouse (926-32210, LI-COR, USA) IRDye 800 CW secondary antibody (1:5000) for 2 h at room temperature. The densities of the specific blots were determined by an Odyssey infrared imaging system (LI-COR Biosciences, Lincoln, NE). To ensure equal loading of samples, β-actin monoclonal antibody (1:10,000, A5316, Sigma, USA) was used as a loading control. The intensity of the specific bands was scanned and analysed with ImageJ software.

### Statistical analysis

Statistical analyses were performed using GraphPad Prism 8.0.1. All the data are presented as the mean ± SEM. Student’s t-test (non-directional) was used to analyze the statistical significance of the data of two groups. Variations between the means in multiple groups were determined by one-way ANOVA, followed by Newman-Keuls post hoc tests. A probability value of *P* < 0.05 was considered statistically significant.

## Results

### HH exposure reduced mouse appetite and stimulated erythropoiesis

**Figure 1:**
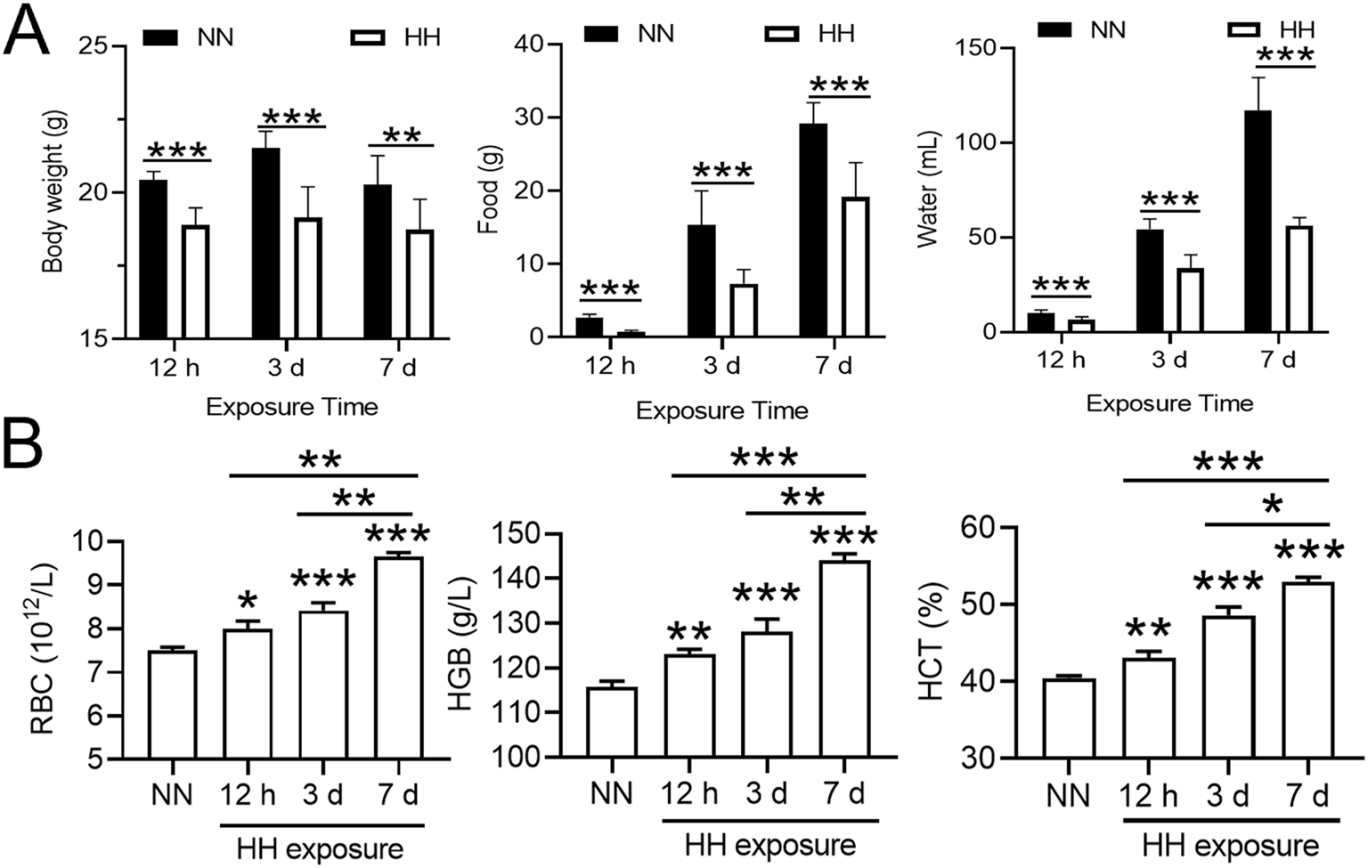
HH exposure reduced mouse body weight and food and water intake and increased RBC, HGB and HCT in the blood of mice. (**A**) Changes in body weight and total (cumulative) food and water intake of mice after 12 h, 3 d and 7 days of HH exposure. (**B**) Changes in RBC, HGB and HCT in the blood of mice after HH exposure for 12 h, 3 and 7 days. Values are displayed as the mean ± SEM (n = 9 mice), * P < 0.05, ** P < 0.01, *** P < 0.001 versus the NN group or the indicated group.

Compared to the NN group, the mice in the HH group appeared to be in a state of lethargy. The activities and excretions of the mice were diminished following HH exposure. Moreover, the cumulative food and water intake of the mice, as well as their body weight, experienced a significant decline after HH exposure for 12 h to 7 days (Fig. 1A). Conversely, the indices of erythropoiesis, including RBC number, HGB content, and HCT in mouse blood, were found to be significantly elevated with increased HH exposure time (Fig. 1B).

### HH exposure increased plasma ghrelin and acylated-ghrelin content

**Figure 2:**
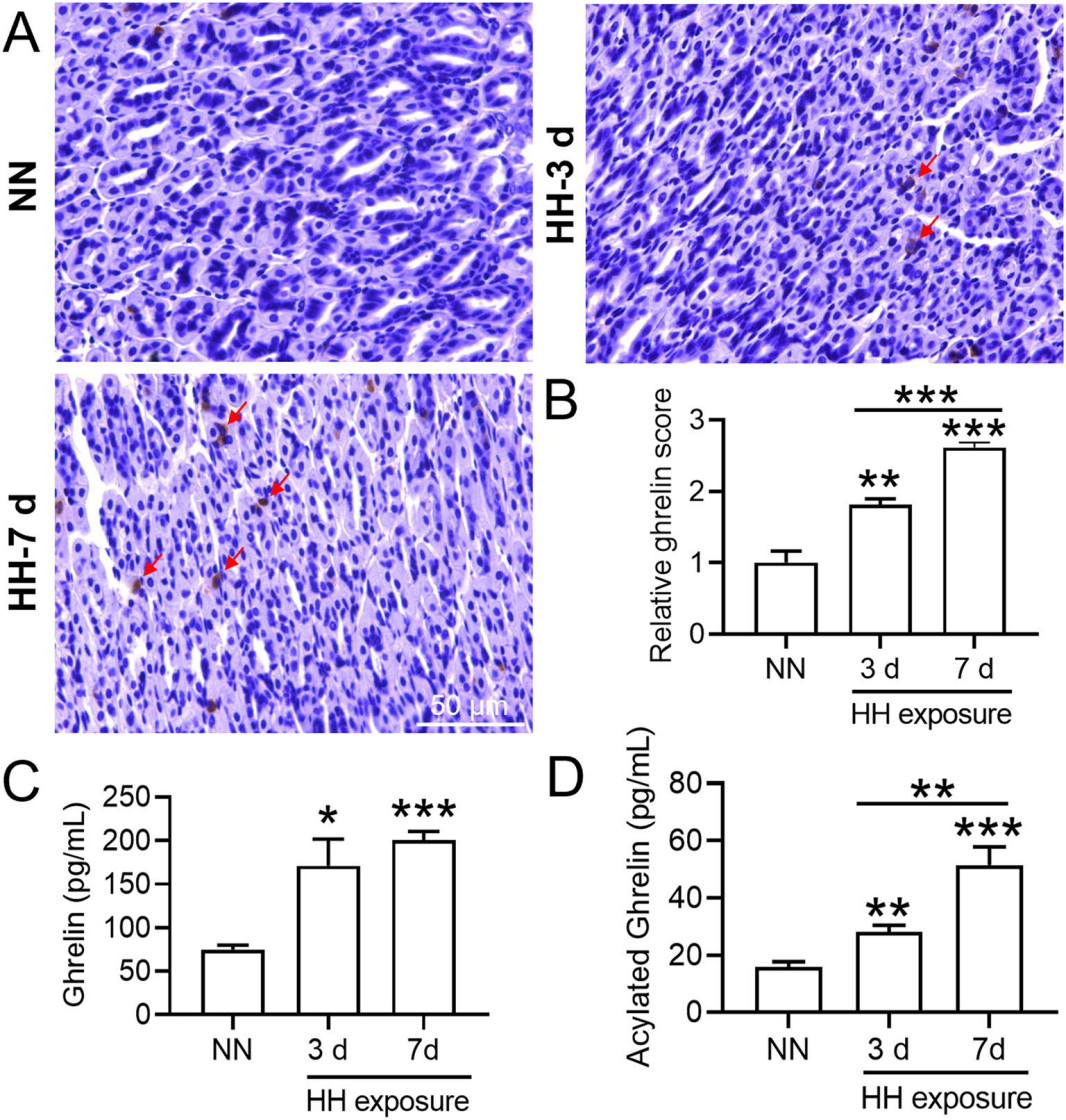
HH exposure induced ghrelin expression in the mouse stomach as well as plasma levels of ghrelin and acylated ghrelin. (**A**) Expression and distribution of ghrelin (red arrow) in the stomach of mice after HH exposure for 3 and 7 days; (**B**) Semiquantitative analysis of ghrelin expression in the stomach. (**C**,**D**) The contents of ghrelin and acylated ghrelin in the plasma of mice after HH treatment for 3 and 7 days. Values are displayed as the mean ± SEM (n = 6 mice), * P < 0.05, ** P < 0.01, *** P < 0.001 versus NN or indicated group.

To determine whether expression of the appetite hormone ghrelin changed after HH exposure, we detected ghrelin content in the stomach and plasma by IHC and ELISA. IHC results showed that ghrelin expression in the mouse stomach increased after HH exposure (Fig. 2A,B). Figure 2C,D demonstrated that 3 and 7 days of HH exposure induced significant elevation of ghrelin and acylated-ghrelin levels in plasma. In addition, the acylated-ghrelin content was increased significantly after HH exposure for 7 days when compared to that measured after 3 days (Fig. 2D). The above results suggested that after exposure to HH for 3 and 7 days, the stomach synthesized and secreted ghrelin into the blood and then was further modified to form acylated ghrelin.

**Figure 3:**
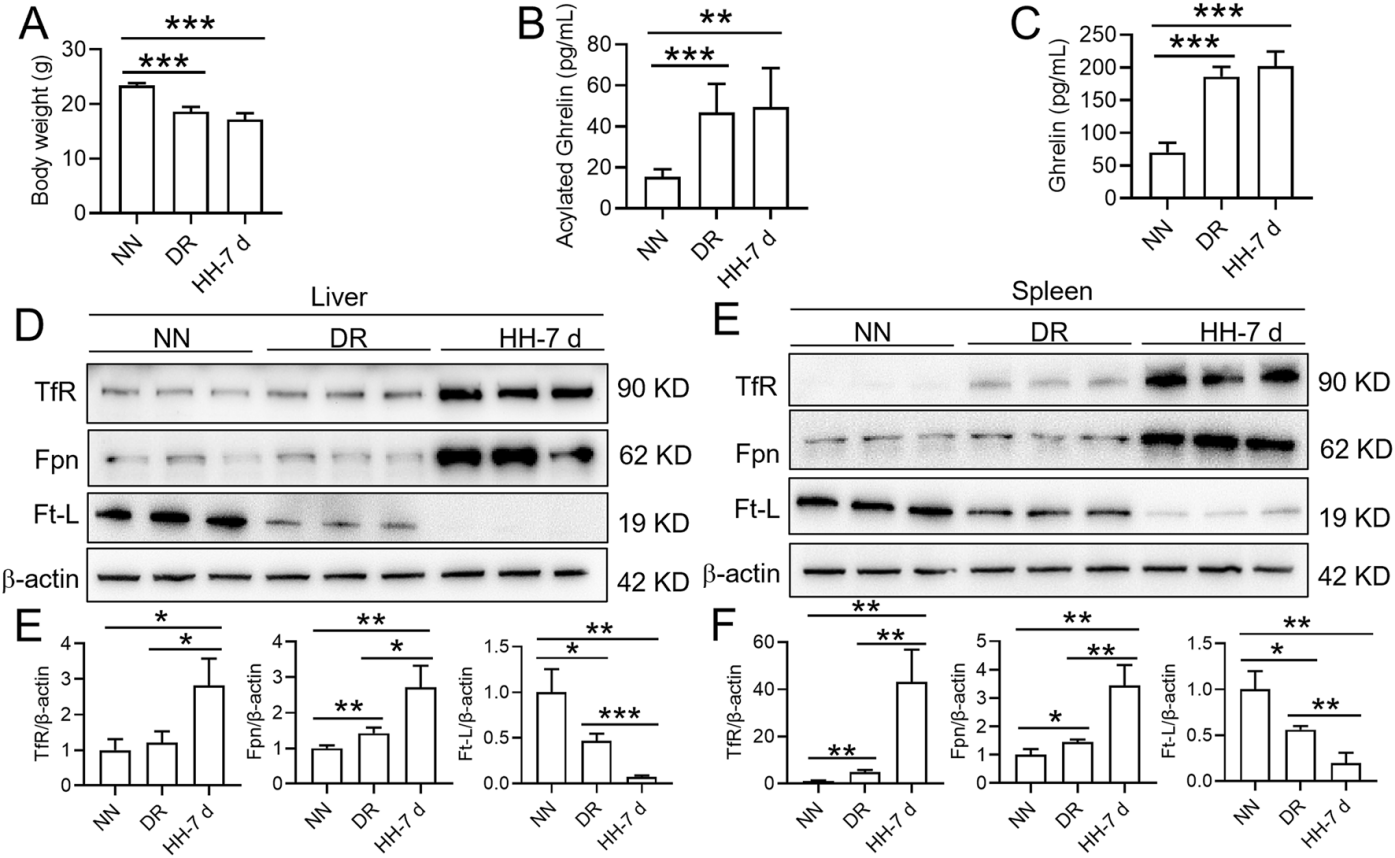
Effects of hypobaric hypoxia (HH) or normoxia dietary restriction (DR) for 7 days on the ghrelin levels and iron metabolism. (**A**) Changes in body weight of mice after 35% DR under normobaric normoxia or HH exposure. (**B**,**C**) The contents of ghrelin and acylated ghrelin in the plasma of mice after DR or HH treatment for 7 days. (**D**,**F**) Western blotting analysis of TfR, Fpn and Ft-L expression in the liver. (**E**,**G**) Western blotting analysis of TfR, Fpn and Ft-L expression in the spleen. Values are displayed as the mean ± SEM (n = 6 mice), * P < 0.05, ** P < 0.01, *** P < 0.001 versus the indicated group.

In order to demonstrate whether hypoxia per se or insufficient dietary intake is responsible for the ghrelin levels and iron metabolism, we also added a pair of control animals with 35% dietary restriction (DR) for 7 days under normoxic condition and made a comparison. Compared with the NN group, the DR and HH treatment groups had lower body weight (Fig. 3A) and significantly increased ghrelin and acylated ghrelin contents (Fig. 3B,C). However, there were no significant differences in the above parameters between the DR and HH groups. We also found that the expression of both TfR and Fpn was significantly enhanced, while the expression of Ft-L was decreased after DR or HH treatment (Fig. 3D–G). However, compared with DR group, the expression of TfR and Fpn was significantly increased in the liver and spleen of HH group, and the expression of Ft-L was suppressed (Fig. 3D–G).

### HH exposure reduced tissue iron content and increased serum iron content

**Figure 4:**
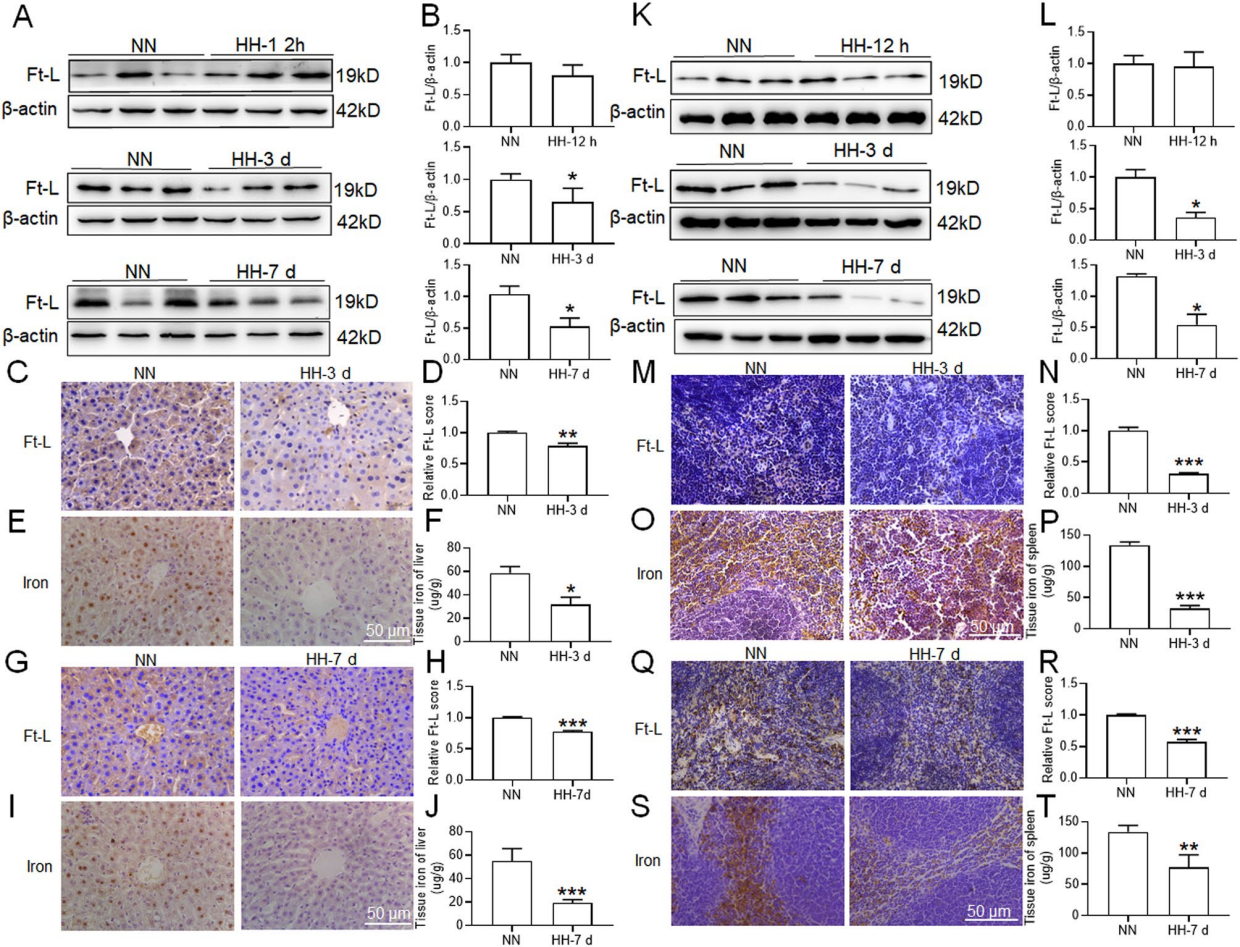
HH exposure induced a reduction in Ft-L protein expression and iron content in the liver and spleen of mice. (**A**,**K**) Expression of Ft-L protein in the liver and spleen of mice after HH exposure for 12 h, 3 d and 7 d; (**B**,**L**) Relative protein expression levels of Ft-L protein expression in panels (**A**) and (**K**); (**C**,**G**) IHC images of Ft-L in the liver and (**M**,**Q**) spleen of mice after HH exposure for 3 d and 7 d; (**D**,**N**,**H**,**R**) Semiquantitative analysis of Ft-L protein expression in panels (**C**) and (**G**), (**M**) and (**Q**); (**E**,**O**,**I**,**S**) Iron staining and tissue iron content in the liver and spleen of mice exposed to HH for 3 and 7 days. Values are displayed as the mean ± SEM (n = 6 mice), * P < 0.05, ** P < 0.01, *** P < 0.001 versus NN or indicated group.

**Figure 5:**
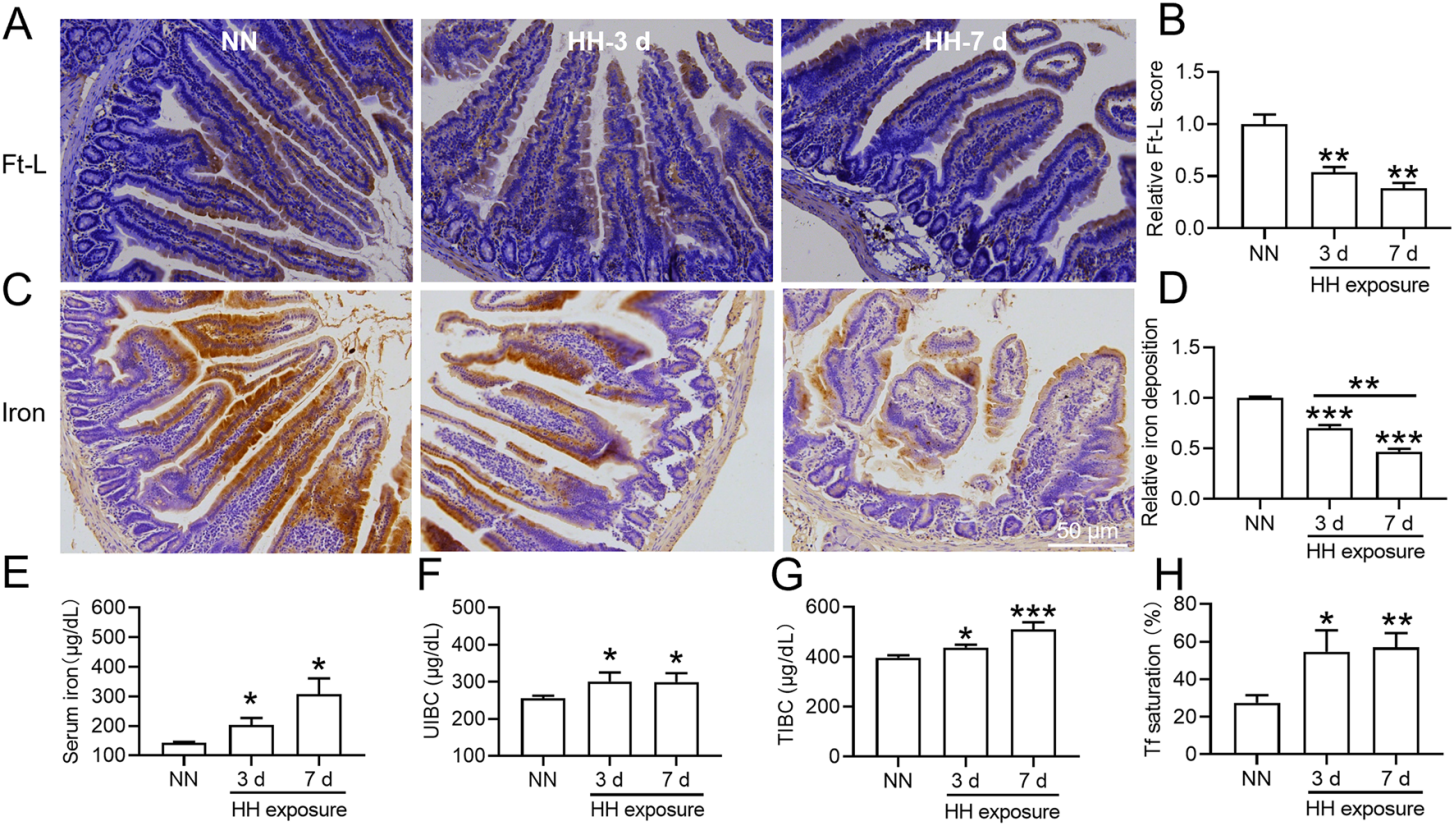
HH exposure induced a reduction in duodenal Ft-L expression and iron content while increasing serum iron, UIBC, TIBC and Tf saturation. (**A**) IHC images of Ft-L in the duodenum of mice after HH exposure for 3 and 7 days; (**B**) Semiquantitative analysis of Ft-L expression in Figure (**A**); (**C**) Iron staining of the duodenum after 3 and 7 days of HH exposure; (**D**) Semiquantitative analysis of iron staining in panel C; (**E**,**F**,**G**,**H**) Changes in serum iron, UIBC, TIBC and Tf saturation after HH exposure for 3 and 7 days. Values are displayed as the mean ± SEM (n = 6 mice), * P < 0.05, ** P < 0.01, *** P < 0.001 versus NN or indicated group.

Due to the increasing demand for iron for HGB synthesis that occurs under HH exposure, we examined the iron content after HH exposure in the mouse liver, spleen and duodenum, which are known as storage, recycling and absorption sites of iron. The results showed that Ft-L expression (Fig. 4A–D,G–H,K–N,Q–R), iron staining (Fig. 4E,I,O,S) and tissue iron content (Fig. 4F,J,P,T) were reduced significantly in the liver and spleen after HH exposure for 3 and 7 days, while Ft-L expression was not changed after HH exposure for 12 h. Then, we measured the iron content in the duodenum. Compared to those of the NN group, duodenal Ft-L expression (Fig. 5A,B) and iron staining (Fig. 5C,D) also decreased significantly after HH exposure for 3 and 7 days. We also measured serum iron content, TIBC, UIBC and Tf saturation after HH exposure for 3 and 7 days. Compared to those of the NN group, levels of all four metrics assessed above were increased in the group exposed to HH for 3 and 7 days (Fig. 5E,H). These results demonstrated that iron was released from the liver, spleen and duodenum into the plasma, which permitted HGB synthesis under HH exposure conditions.

### HH exposure accelerated iron mobilization in the liver and spleen of mice

**Figure 6:**
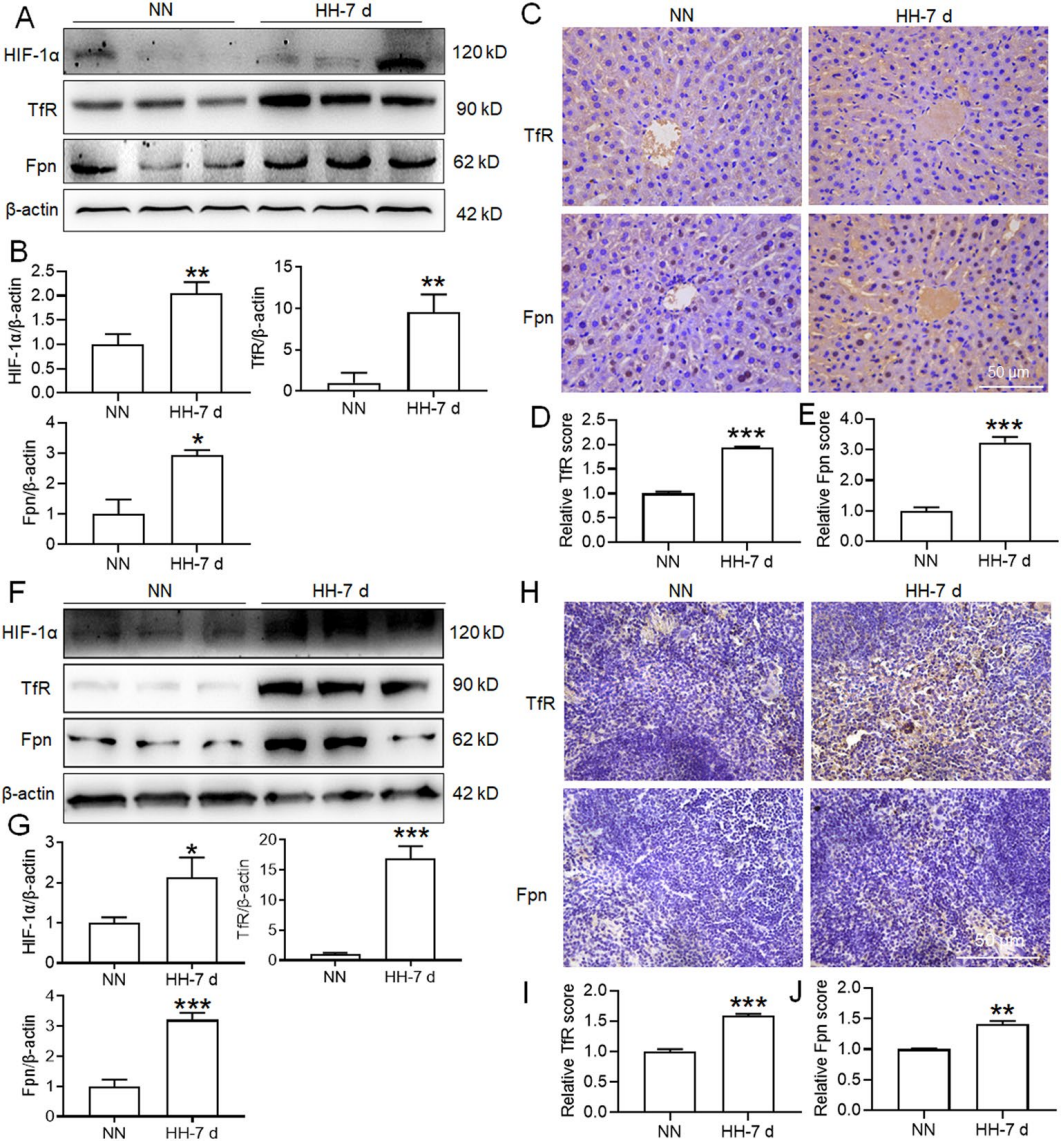
HH exposure of 7 days promoted TfR and Fpn expression in the liver and spleen of mice. (**A**,**F**) Western blotting analysis of HIF-1α, TfR and Fpn expression in the liver and spleen after 7 days of HH exposure. (**B**,**G**) Semiquantitative analysis of HIF-1α, TfR and Fpn expression in panels (**A**) and (**F**). (**C**,**H**) IHC images of TfR and Fpn expression in the liver and spleen after 7 days of HH exposure. (**D**–**E**,**I**–**J**) Semiquantitative analysis of TfR and Fpn expression in panels (**C**) and (**H**). Values are displayed as the mean ± SEM (n = 6 mice), * P < 0.05, ** P < 0.01, *** P < 0.001 versus the NN or indicated group.

We next investigated iron mobilization in the liver and spleen of mice after HH exposure, which was reflected by the expression of TfR and Fpn. Our findings indicate that with HH exposure for 7 days, all the protein expressions, including HIF-1α, exhibited changes similar to those observed in our primary outcomes. However, it's worth noting that after 12 h of HH exposure, we did not observe any significant change in the expression of HIF-1α or any of the other proteins we monitored (data not shown). Here, we only presented the results of HH treatment for 7 days. As shown in Fig. 6A–B,F–G, HIF-1α expression increased significantly in the liver and spleen after 7 days of HH treatment, which suggested that the liver and spleen were subjected to hypoxia after 7 days of HH exposure. Notably, the results of IHC and Western blot analyses both showed that TfR and Fpn expression increased significantly after HH treatment for 7 days (Fig. 6C–E,H–J). These results suggested that 7 days of HH treatment promoted iron uptake and export, which facilitated iron mobilization in the liver and spleen.

### Ghrelin induced Fpn expression via activation of the MAPK signalling pathway under hypoxia in both primary hepatocytes and peritoneal macrophages

**Figure 7:**
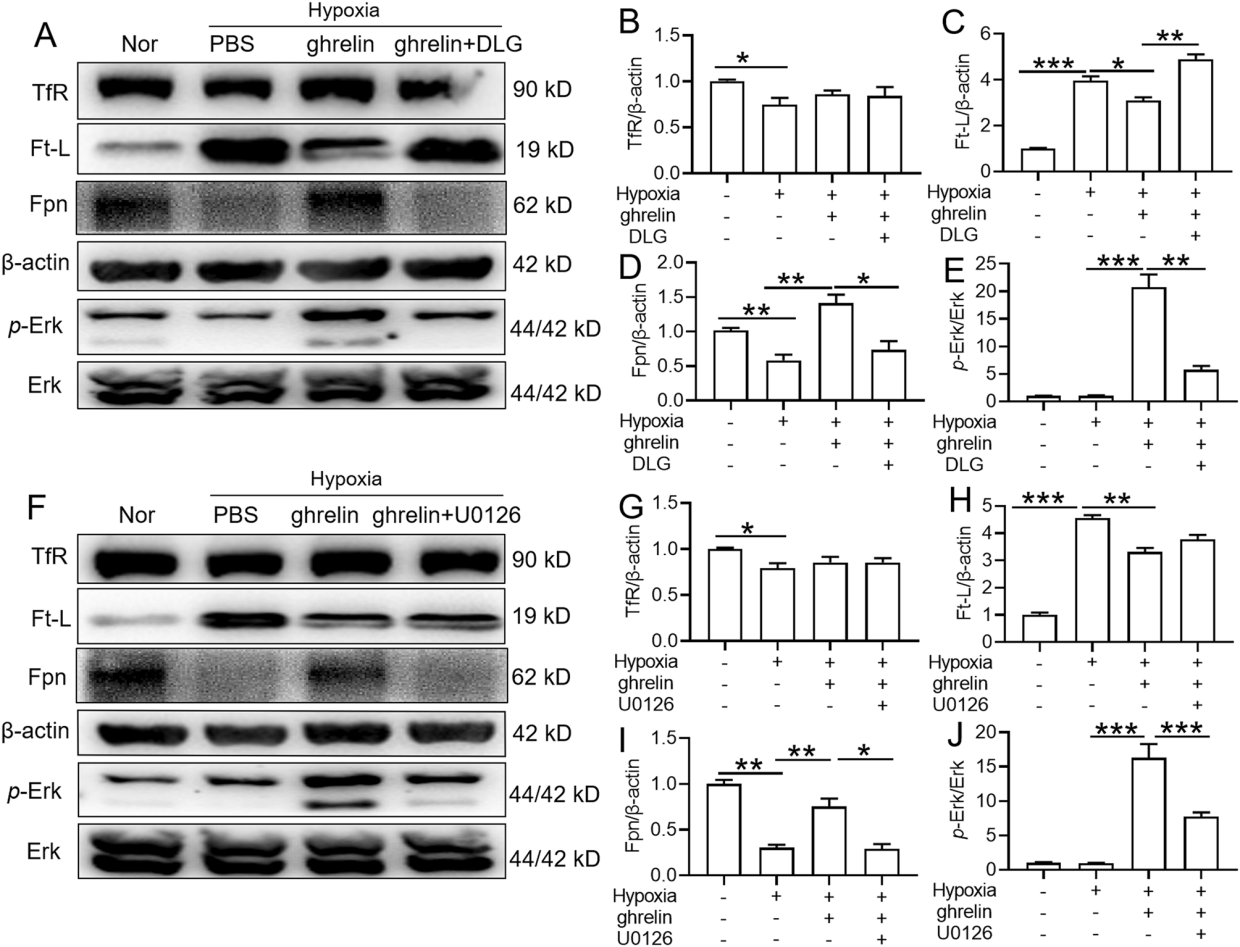
Effects of ghrelin on expression of iron-regulating proteins and Erk phosphorylation in mouse hepatocytes under hypoxia condition. (**A**,**F**) Primary hepatocytes of mice were pretreated with a GHSR1a receptor antagonist (DLG, 100 nM) or MAPK signalling pathway inhibitor (U0126) for 1 h, followed by 1% O_2_ treatment with or without the presence of ghrelin (10^–7^ M) for 12 h. The relative expression levels of TfR, Ft-L, Fpn, pErk/ErK were detected by WB. (**B**–**E**,**G**–**J**) Semiquantitative analysis of TfR, Ft-L, Fpn, pErk/ErK in panels (**A**) and (**F**). Values are displayed as the mean ± SEM (n = 3 independent experiments), * P < 0.05, ** P < 0.01, *** P < 0.001 versus NN or the indicated group.

**Figure 8:**
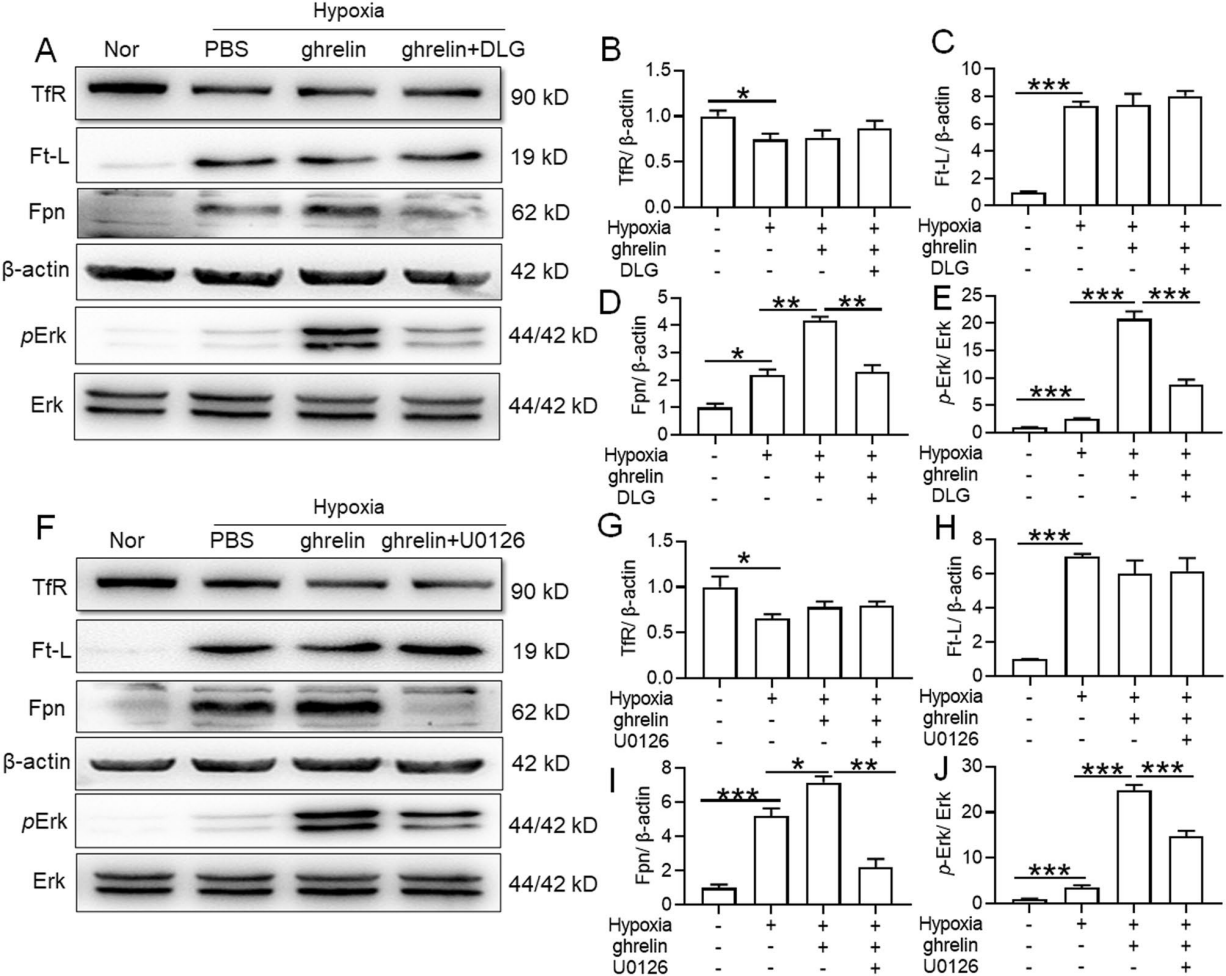
Effects of ghrelin on expression of iron-regulating proteins and Erk phosphorylation in mouse peritoneal macrophages under hypoxia. (**A**,**F**) Peritoneal macrophages of mice were pretreated with GHSR1a receptor antagonist (DLG, 100 nM) or MAPK signalling pathway inhibitor (U0126) for 1 h, followed by 1% O_2_ treatment with or without the presence of ghrelin (10^–7^ M) for 12 h. The expressions of TfR, Ft-L, Fpn, pErk/ErK were detected by WB; (**B**–**E**,**G**–**J**) Semiquantitative analysis of TfR, Ft-L, Fpn, pErk/ErK in panels (**A**) and (**F**). Values are displayed as the mean ± SEM (n = 3 independent experiments), * P < 0.05, ** P < 0.01, *** P < 0.001 versus NN or indicated group.

We next investigated whether HH-induced ghrelin was involved in the regulation of iron metabolism in the liver and spleen. We used primary hepatocytes and macrophages to observe the ghrelin response. Our preliminary results showed that HIF-1α expression increased significantly at 12 h of hypoxia both in hepatocytes and macrophages (data not shown). Therefore, 12 h of hypoxia treatment was used in our subsequent study. We then studied the effect of ghrelin on iron-related protein expression and explored whether the MAPK pathway was involved in this process both in hepatocytes (Fig. 7) and macrophages (Fig. 8). The results showed that hypoxia significantly activated Ft-L expression and inhibited TfR expression when compared to those of the normoxic group both in primary hepatocytes and macrophages (Figs. 7, 8A–C). Although Fpn expression was decreased in hepatocytes and increased in macrophages under hypoxia when compared to the normoxic group, exogenous ghrelin administration obviously induced Fpn and pErK expression under hypoxia both in hepatocytes and macrophages. And, pretreatment with DLG and U0126 suppressed the effects of ghrelin on Fpn and pErK expression both in hepatocytes and macrophages (Figs. 7, 8A,D–F,I–J). In addition, ghrelin administration had no effect on TfR both in hepatocytes and macrophages under hypoxia. However, although ghrelin had no effect on the expression of Ft-L in macrophages, it significantly reduced Ft-L expression in hepatocytes in hypoxic condition. Although DLG inhibited the effects of ghrelin on Ft-L expression in hepatocytes, both DLG and U0126 had no effect on TfR expression in the two cell lines under hypoxia (Figs. 7, 8A–C,F,G–H). These results demonstrated that ghrelin upregulated Fpn expression specifically via activation of the GHSR1a/MAPK signalling pathway under hypoxia. Besides, ghrelin downregulated Ft-L expression via GHSR1a but not activate MAPK signalling pathway in hepatocytes under hypoxia.

## Discussion

The main purpose of present study was to investigate whether ghrelin was involved in and how ghrelin affects iron mobilization in mouse liver/spleen and primary hepatocytes/peritoneal macrophages under HH or hypoxia exposure conditions. The present study employed a hypobaric chamber to simulate a 6000-m altitude exposure, which is physiologically relevant in the context of understanding the effects of HH on various physiological parameters^31,32^. The altitude chosen falls within the range that has been previously demonstrated to induce significant physiological responses^32^. The use of a hypobaric chamber provides a controlled environment to observe the effects of hypobaric hypoxia, ensuring consistency and reproducibility in the experimental conditions^31^. These results showed that HH exposure led to a significant reduction in food and water intake as well as in weight loss in mice. Interestingly, the decrease in food intake in HH animals was nearly equivalent to that in diet restriction (DR) group.

Ghrelin is an appetite-inducing brain-gut peptide secreted by endocrine cells in the stomach and other tissues^19^. Ghrelin is acylated on its serine 3 residue by ghrelin O-acyltransferase and the acylated ghrelin plays a role in stimulating gastric emptying and further prompting ingestion^33,34^. Previous studies have reported that both fasting and dietary restriction could induce the ghrelin expression^24,28,33^. Here we also observed a significant increase in serum iron as well as plasma ghrelin and acylated ghrelin levels after HH exposure. Our results were similar to those of other studies that showed ghrelin levels were increased after high altitude exposure for 14 days in men^23^ and in hypoxia-induced anorexia rat models^22^. To further demonstrate whether HH per se or the less food intake is responsible for the ghrelin levels and iron mobilization, a paired of control animals with 35% DR for 7 days under normoxic condition were also added and compared. However, there were no significant differences on the body weight, ghrelin and acylated ghrelin contents between the DR and HH groups. HH-induced ghrelin expression may be attributed to two primary factors: the dietary restriction caused by HH exposure and the direct effects of hypoxia itself. Both of these factors have the potential to induce ghrelin expression. This dual influence in the HH group could explain the more pronounced effects observed in iron metabolism-related protein expressions compared to the DR group. Our data implied that a reduction in food intake stimulates ghrelin synthesis and secretion, as well as the acylation of ghrelin in the blood, suggesting that insufficient dietary intake may play a major role in ghrelin expression caused by HH exposure. In addition, the HH group exhibited more pronounced alterations in TfR, Ft-L, and Fpn expression compared to the DR group, notwithstanding the similar ghrelin expression observed between the two groups. The inhibition of hepcidin expression after 7 days of HH exposure (data not presented) aligns with findings reported in the literature under conditions of hypoxia or HH exposure^35–37^. The results imply that the body may concurrently employ ghrelin and hepcidin pathways to expedite iron efflux from the liver and spleen, satisfying the heightened iron demands for new RBC synthesis during hypobaric hypoxia exposure. However, it remains to be conclusively determined whether additional factors (e.g., HIF-2, DMT1) or pathways are implicated in iron efflux, particularly in the regulation of Fpn expression. These findings underscore the potential for ghrelin-independent mechanisms in the HH group, highlighting the complex nature of iron homeostasis in response to hypoxic conditions, as previously mentioned.

Moreover, changes in iron levels in the liver, spleen and duodenum under HH conditions were examined because the liver is the main site of iron storage site^38,39^, the spleen is a key organ for iron recycling^14,40^, and the duodenum is the main site of iron absorption^41^. We found that the iron content indicated by Ft-L expression, iron staining or iron detection was significantly decreased in the liver, spleen and duodenum after HH for 3–7 days, which suggested that iron mobilization, especially iron release, in these tissues was enhanced. We further measured TfR and Fpn expression to assess iron mobilization in the liver and spleen because TfR is a major iron importer, while Fpn is the only known iron exporter in cells^42,43^. The increased TfR and Fpn expression in the liver and spleen after 7 days of HH also verified that iron mobilization in the two tissues was activated. These data were consistent with results from another study in rats, which demonstrated that intermittent HH induced TfR and Fpn expression both in the liver and spleen^17^. Moreover, the increased serum iron and Tf saturation in the blood induced by HH further hinted that the hepatic and splenic iron was released into the blood for utilization by bone marrow for erythropoiesis. In contrast, HH induced a significant increase in RBC production in the blood.

An investigation of iron deficiency anaemia confirmed that iron content was tightly related to ghrelin levels in the blood^44^. Our previous studies also demonstrated that total/acylated ghrelin content was closely related to iron levels both in humans and mice^28^. In addition, we also confirmed that ghrelin induces iron export by increasing Fpn expression both in the liver and spleen^24,25^. Thus, we speculated that the increased ghrelin expression induced by HH may affect iron metabolism, especially iron export, both in the liver and spleen. In addition, in order to exclude the hepcidin effects on iron metabolism after HH in vivo, which was suppressed by erythropoietin-induced erythropoiesis and led to further increased Fpn expression in cells under hypoxia^9,45^. We investigated the effects of ghrelin on the expression of Ft-L, Fpn, and TfR proteins in primary hepatocytes and peritoneal macrophages under hypoxia. Hepatocytes are the main iron storage cell type in the liver, and the RPM in the spleen is the dominant cell population responsible for clearing and recycling iron from senescent or damaged erythrocytes and providing most of the iron for erythropoiesis^46,47^. We observed that iron storage reflected by Ft-L expression was increased both in hepatocytes and macrophages after hypoxia exposure, while iron uptake reflected by TfR expression was reduced after hypoxia treatment.

Interestingly, Fpn expression was reduced in hepatocytes and was induced in macrophages. The differences of iron metabolism status between the two cell lines under hypoxia may be because hepatocytes contain more iron than macrophages under physiological conditions^47,48^, which results in different reactions to Fpn expression under hypoxia. In addition, Fpn expression might be altered by macrophage differentiation and polarization which can be affected by hypoxia. It is noteworthy that increased Fpn expression is typically indicative of M2 macrophage polarization, as supported by existing literature^49,50^. This underscores the cell-specific regulation of Fpn transcription, suggesting that a unified model might not always capture the nuances of Fpn expression across different cellular contexts. However, after exposing the two cell lines to hypoxia, ghrelin stimulated Fpn expression both in hepatocytes and macrophages, and the effect of ghrelin was reversed by the pretreatment of GHSR1a receptor antagonist DLG and MAPK signalling pathway inhibitor U0126. We observed differences in the in vitro hypoxic responses of Fpn between hepatocyte and macrophage cell lines. It is likely that Fpn regulation is multifaceted, with FPN mRNA levels exhibiting variability depending on the cell type and context. Thus, tissue content may play a role in Fpn expression. The regulation of Fpn is inherently complex and can be influenced by several factors, leading to cell-specific responses^51^. Since Fpn was required for the efficient mobilization of body iron stores in mice^52^, it suggested that iron mobilization enhanced after ghrelin treatment under hypoxia.

Furthermore, Ft-L expression was only decreased in hepatocytes, while it was not changed in macrophages after hypoxia treatment in the presence of ghrelin. Ft-L expression was only reversed by DLG pretreatment, while U0126 had no effects on it. These results suggested that ghrelin activates Fpn expression via the GSHR1a/MAPK signalling pathway both in hepatocytes and macrophages, while Ft-L may be influenced by another signalling pathway via activation of the ghrelin/GHSR1a system in hepatocytes under hypoxia. Many studies have also reported that ghrelin functions in growth and in mitigating injury through the activation of the MAPK signalling pathway in various cellular systems^34,53^. Iron is a crucial factor in growth and injury processes^54^. Investigating whether ghrelin affects iron metabolism in these processes is an area that warrants future exploration. Although our study focused solely on the effects of ghrelin on iron mobilization, several questions remain unanswered. For instance, it is unclear whether ghrelin has a similar impact on iron metabolism in other tissues or cells in living organisms. Additionally, the reason behind ghrelin's differential effect on Ft-L expression in hepatocytes and macrophages requires further investigation. While our in vitro findings shed light on the direct influence of ghrelin on these proteins, it is important to acknowledge the broader and intricate role of hepcidin in maintaining iron homeostasis during hypoxia in living organisms.

**Figure 9:**
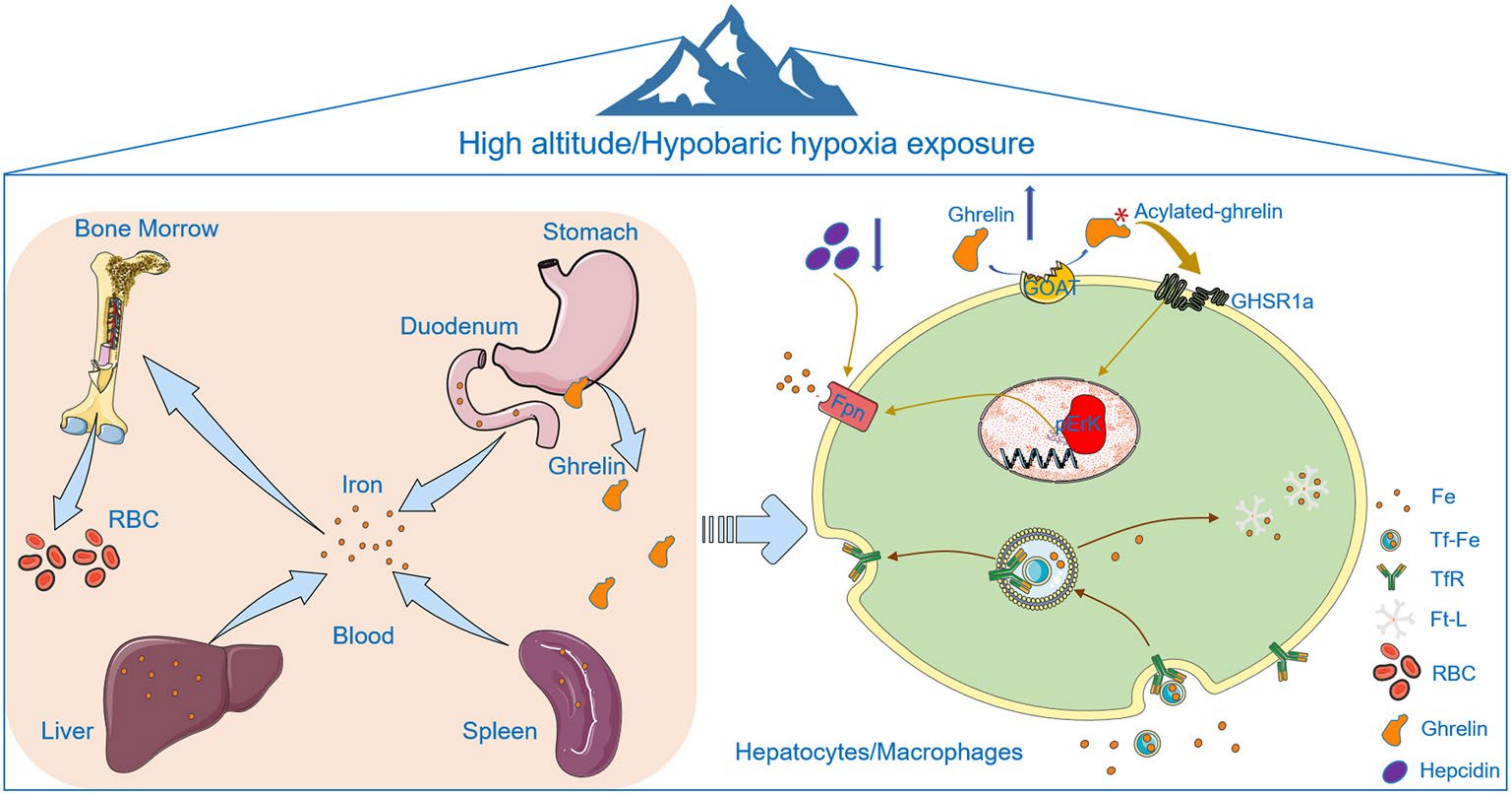
Elucidated pathways and impacts of ghrelin on iron mobilization and export amidst hypobaric hypoxia (HH) conditions. In the course of this investigation, we observed a prominent release of iron from the duodenum, liver, and spleen into the bloodstream, a physiological adaptation aimed at fulfilling the augmented iron requirements for RBC synthesis under HH conditions. This phenomenon appears to be intricately associated with elevated ghrelin levels and diminished hepcidin concentrations in the plasma. Concurrently, we discerned an enhanced exportation of iron from the liver, specifically hepatocytes, as well as from spleen macrophages. This is facilitated through an upregulation of Fpn expression, meticulously orchestrated by the synergistic interaction of the activated Ghrelin/GHSR1a/MAPK signalling pathway and the reduction of hepcidin under conditions of HH and hypoxia.

This study provides direct evidence for the involvement of ghrelin in iron regulation in the liver/hepatocytes and spleen/macrophages under HH or hypoxia. These results indicated that to meet the iron requirements of increased RBC production induced by HH, iron was mainly released from the duodenum, liver, and spleen into the blood for HGB synthesis in bone marrow. During this intricate process of iron homeostasis, it has been discerned that, apart from the hepcidin pathway, ghrelin exerts a substantial regulatory influence on iron mobilization and export. This regulatory activity is mediated through the activation of Fpn expression, which occurs via the GHSR1a/MAPK signalling pathway under both HH and hypoxic conditions. This mechanism is operational in the liver, affecting hepatocytes, as well as in the spleen, impacting macrophages in mice, as illustrated in Fig. 9. Based on these investigative findings, we have elucidated that ghrelin may play a pivotal role in maintaining the equilibrium of iron homeostasis, extending its influence across both physiological and HH conditions. This revelation positions ghrelin as a potential hormone involved in the regulation of iron, highlighting its significance in the broader context of iron homeostasis.

### Supplementary Information

Supplementary Information.

## Data Availability

Full scans of the entire original gels displayed in the manuscript are included as supplementary files.

## Abbreviations

HH: Hypobaric hypoxia
NN: Normobaric normoxia
DR: Diet restriction
DMT1: Divalent metal transporter 1
RBC: Red blood cell
HCT: Haematocrit
HGB: Haemoglobin
MCH: Mean corpuscular haemoglobin
GHSR1a: Growth hormone secretagogue receptor 1a
DLG: D-(lys-3)-GHRP-6
RPM: Red pulp macrophage
HIF-1α: Hypoxia inducible factor 1α
TfR: Transferrin receptor
Ft-L: Ferritin light chains
Fpn: Ferroportin
