# Supplementary material for: Hypobaric hypoxia induces iron mobilization from liver and spleen and increases serum iron via activation of ghrelin/GHSR1a/MAPK signalling pathway in mice
Source: Sci Rep. 2023 Nov 20;13:20254. doi: 10.1038/s41598-023-47596-6 (PMC10662372; doi:10.1038/s41598-023-47596-6)

Blots used in the manuscript

Fig 3D-liver-actin

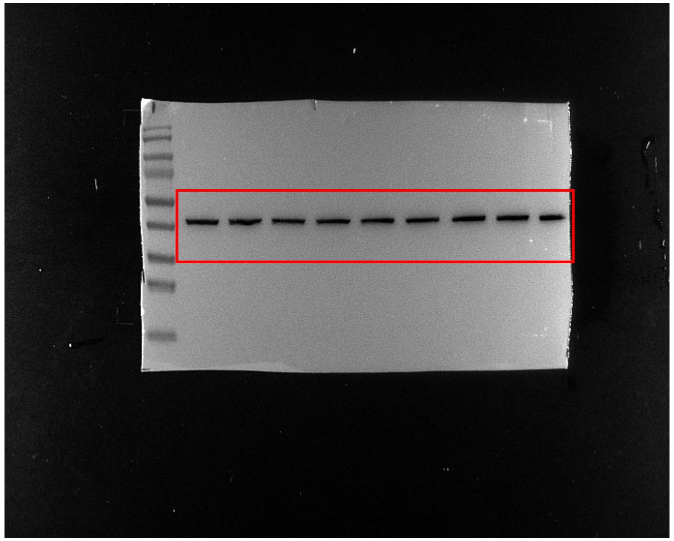

Fig 3D-liver-Fpn

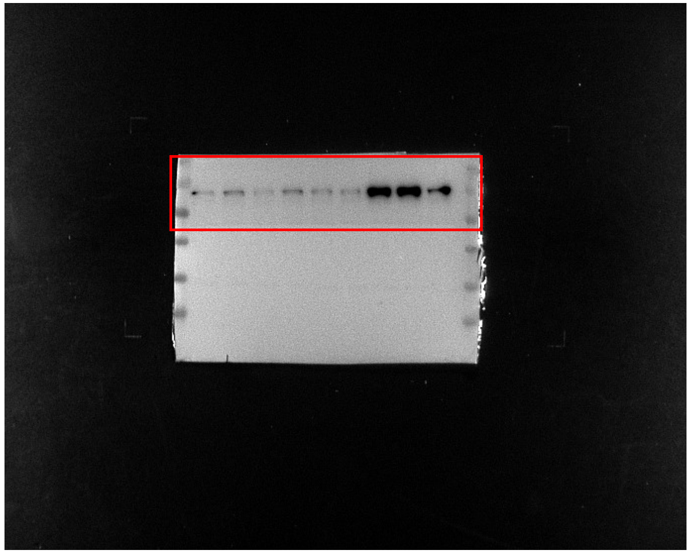

Fig 3D-liver-FtL

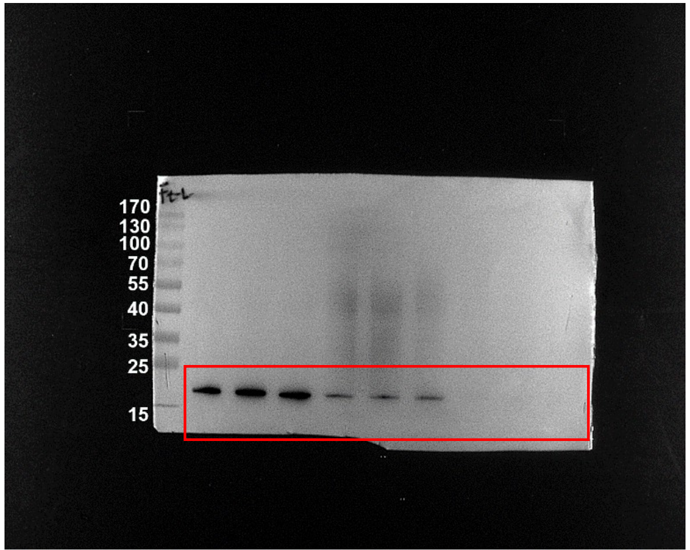

Fig 3D-liver-TfR

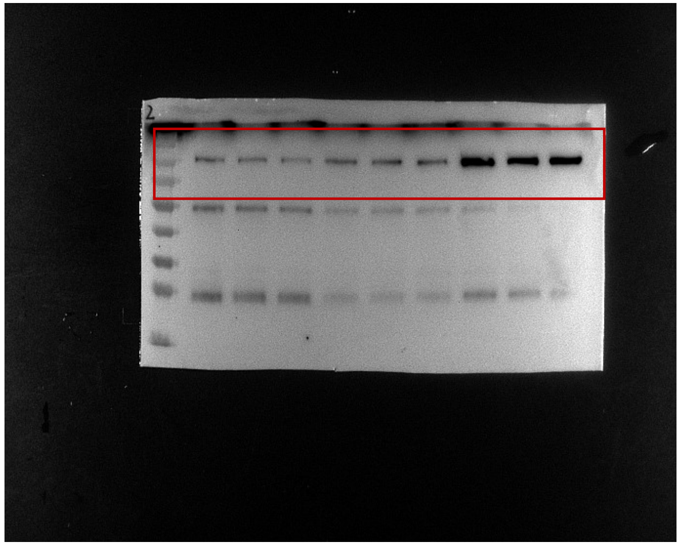

Fig 3E-spleen-actin

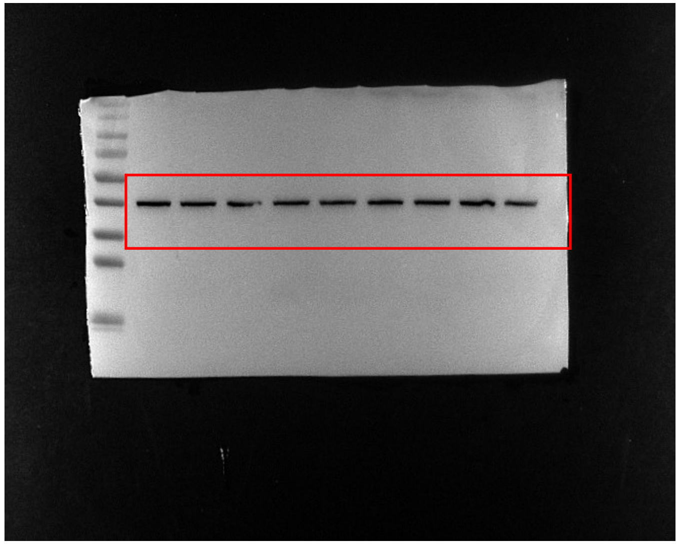

Fig 3E-spleen-Fpn

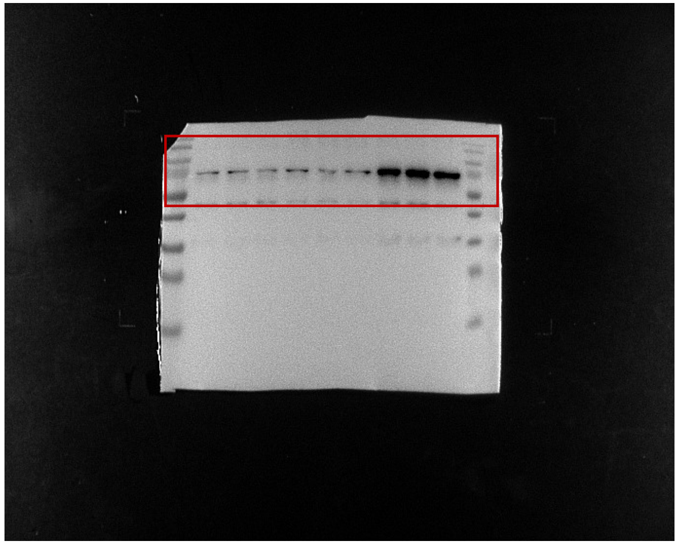

Fig 3E-spleen-FtL

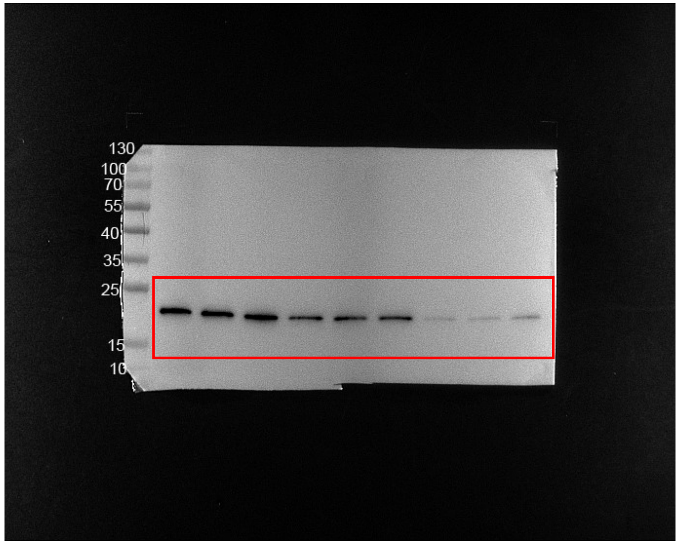

Fig 3E-spleen-TfR

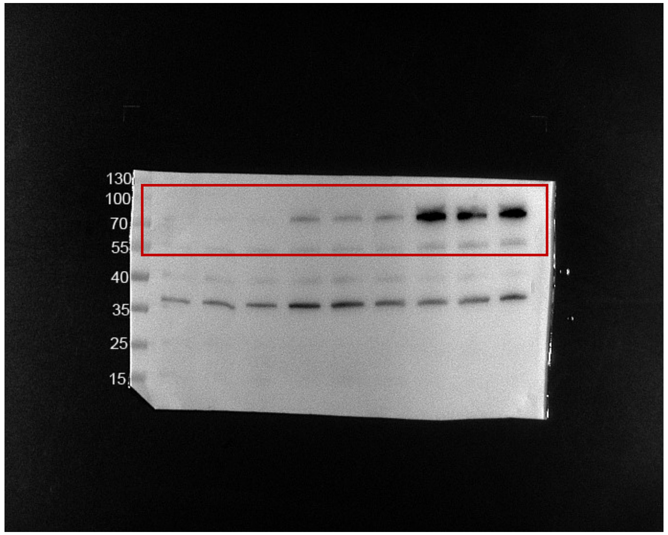

Fig 4A-liver-FtL-12h

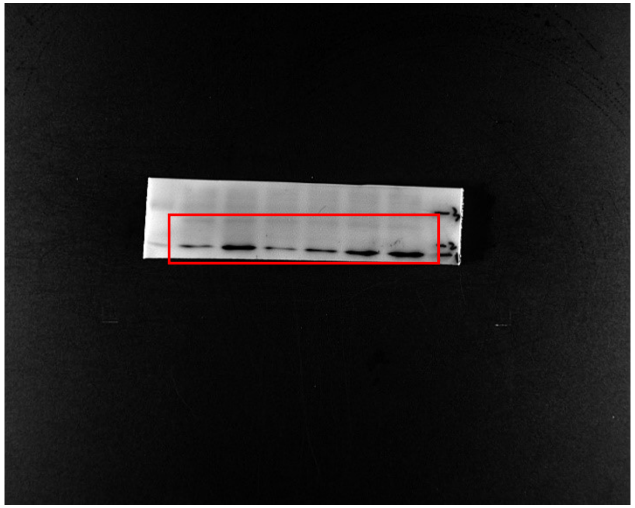

Fig 4A-liver-actin-12h

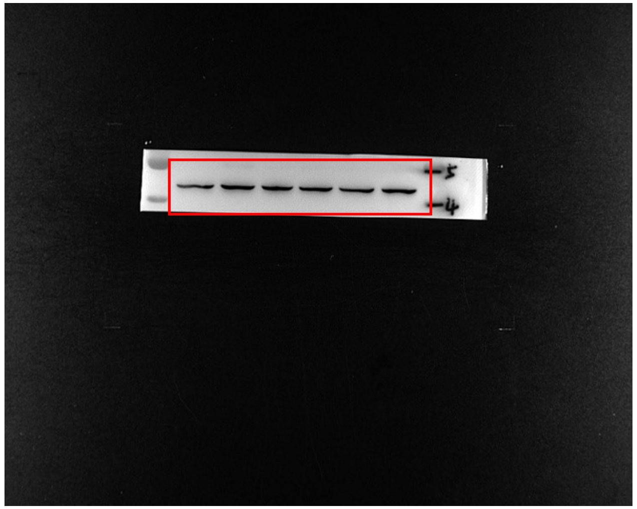

Fig 4A-liver-FtL-3d

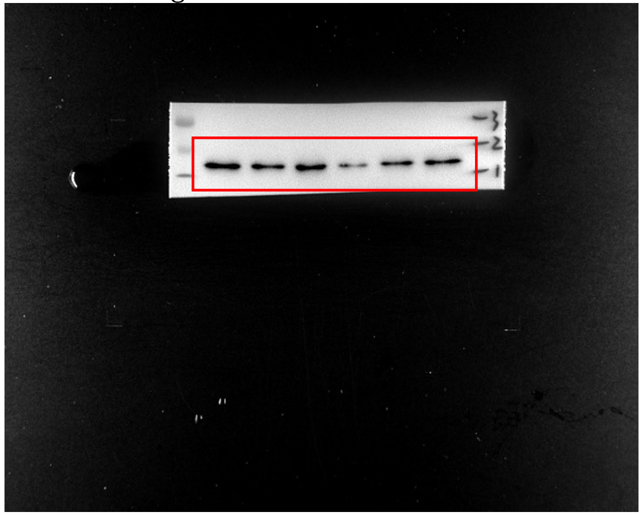

Fig 4A-liver-actin-3d

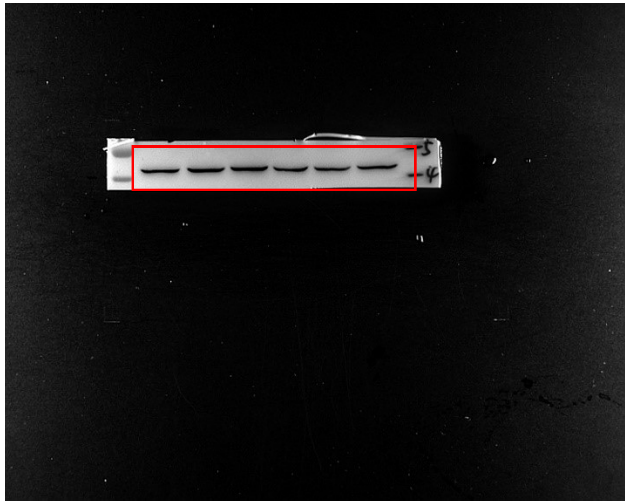

Fig 4A-liver-FtL-7d

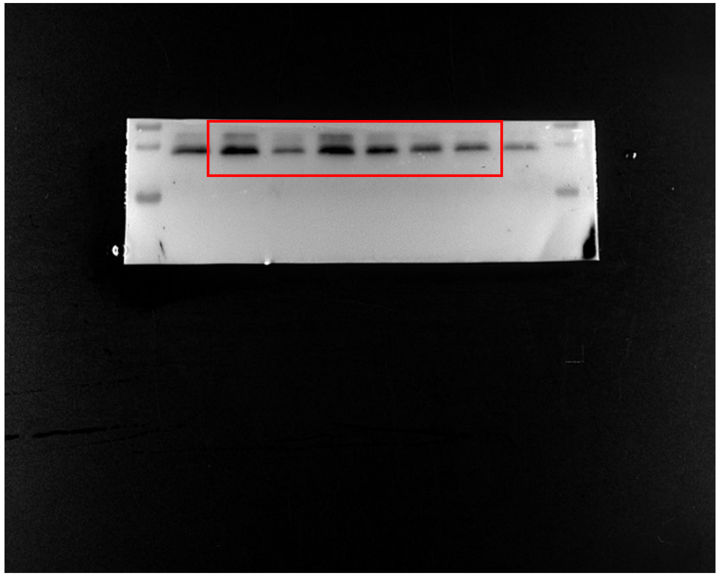

Fig 4A-liver-actin-7d

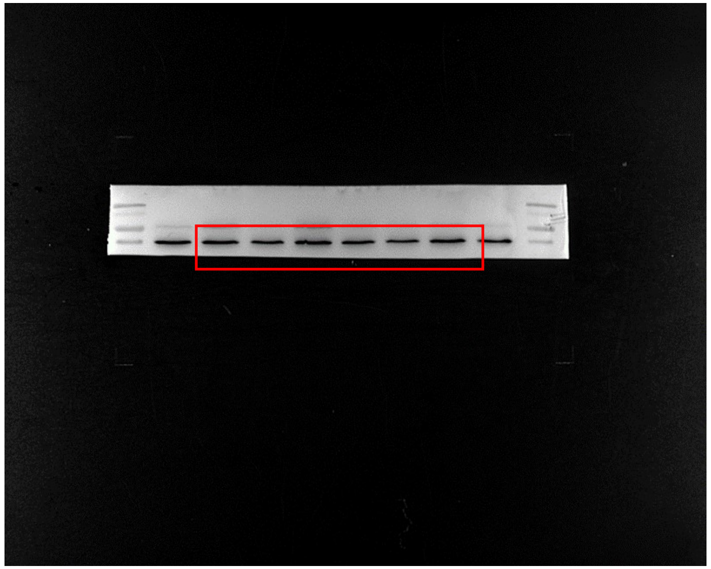

Fig 4K-spleen-FtL-12h

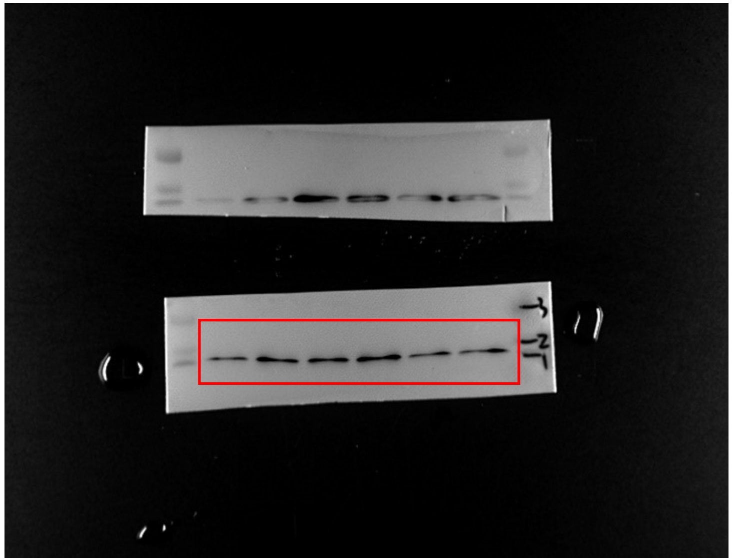

Fig 4K-spleen-actin-12h

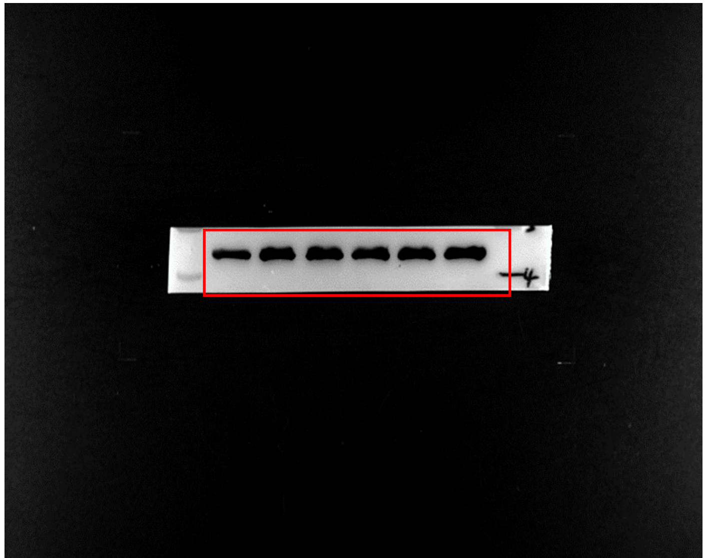

Fig 4K-spleen-FtL-3d

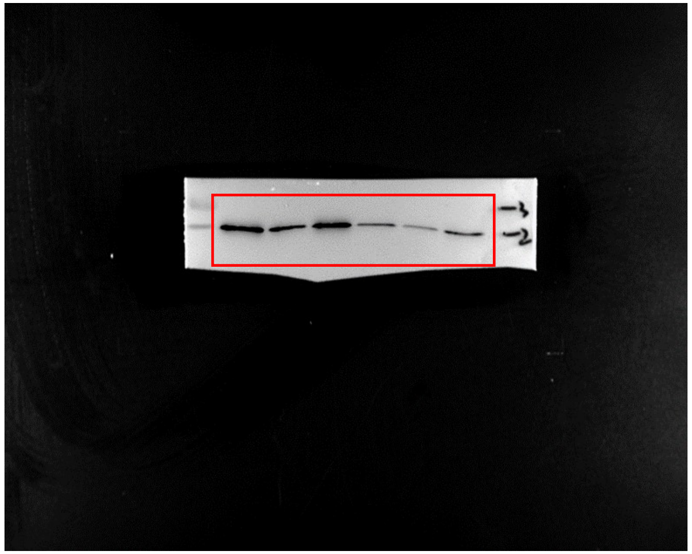

Fig 4K-spleen-actin-3d

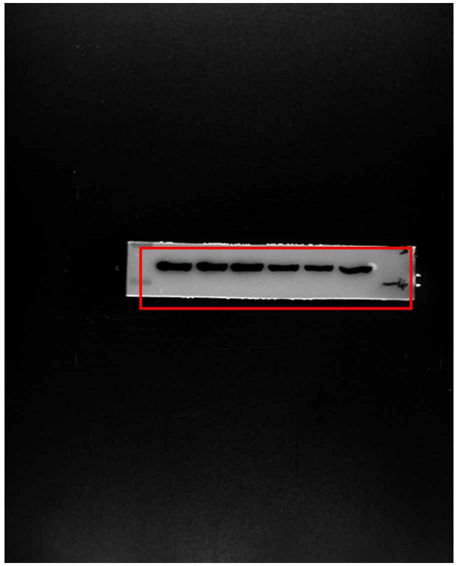

Fig 4K-spleen-FtL-7d

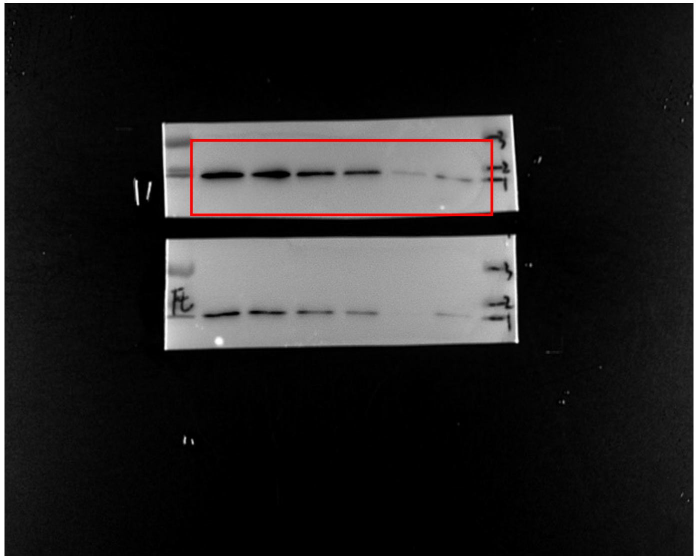

Fig 4K-spleen-actin-7d

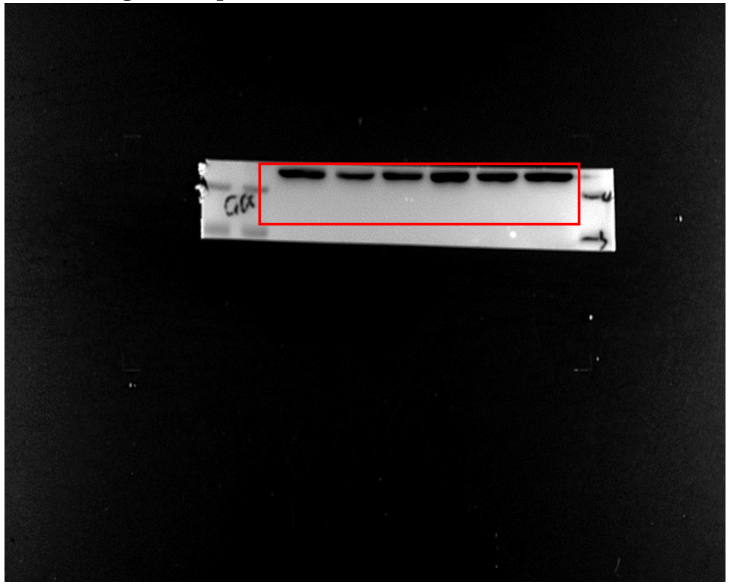

Fig 6A-liver-hif

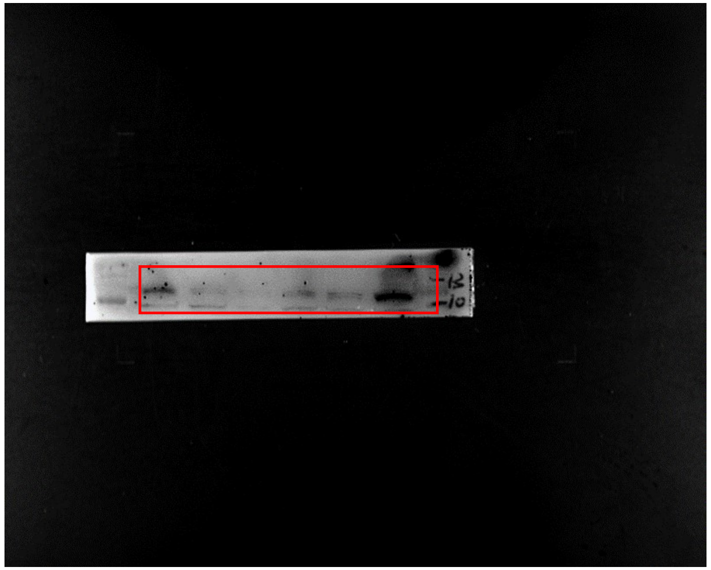

Fig 6A-liver-TfR

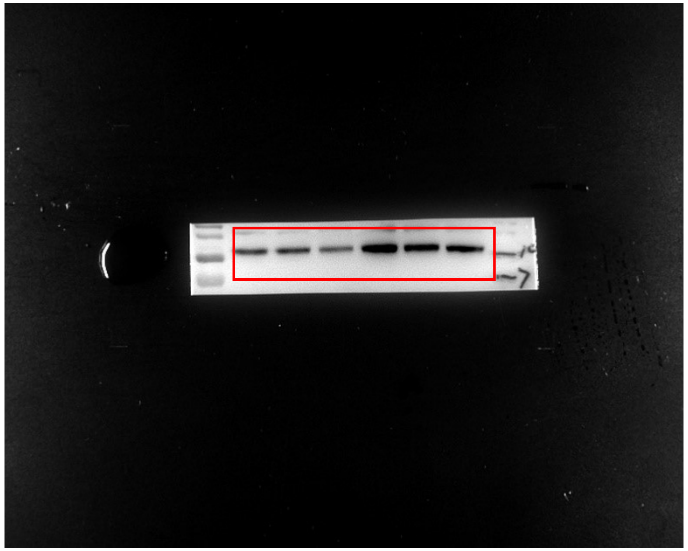

Fig 6A-liver-Fpn

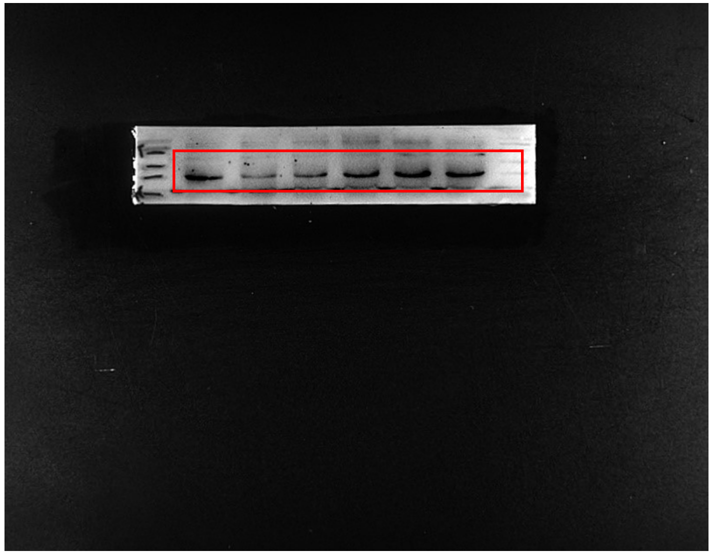

Fig 6A-liver-actin

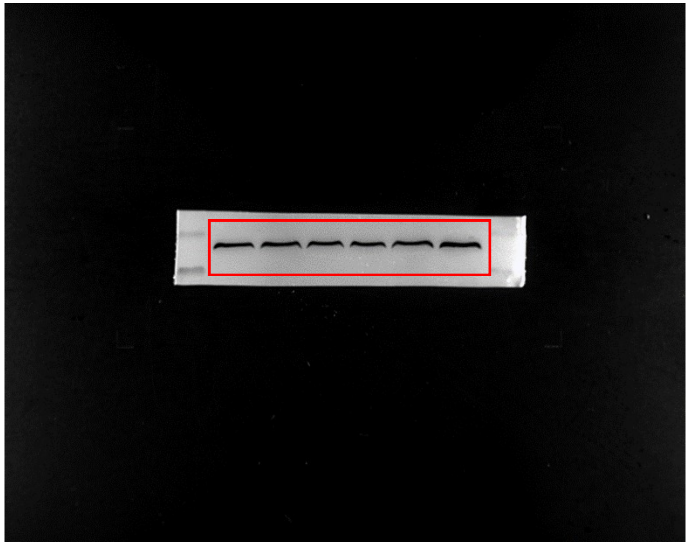

Fig 6F-spleen-hif

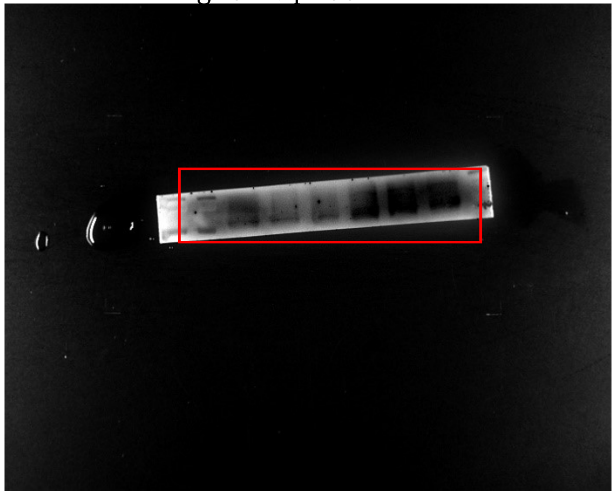

Fig 6F-spleen-TfR

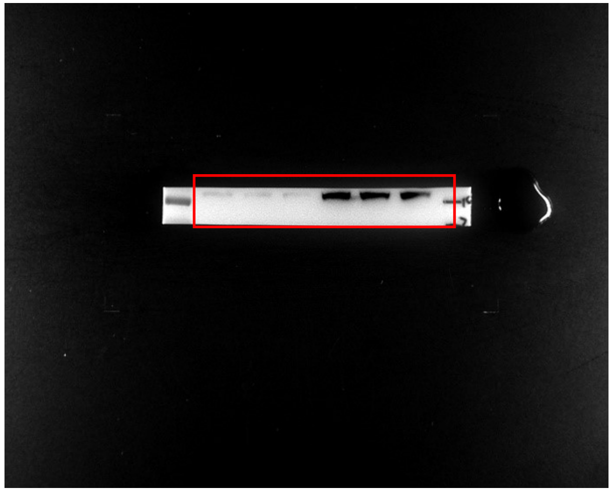

Fig 6F-spleen-Fpn

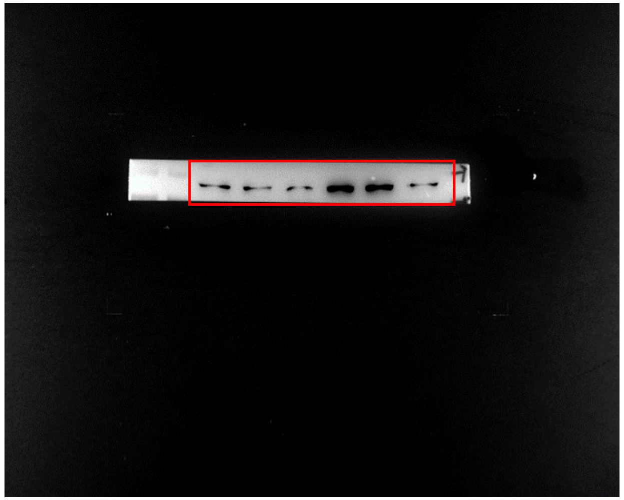

Fig 6F-spleen-actin

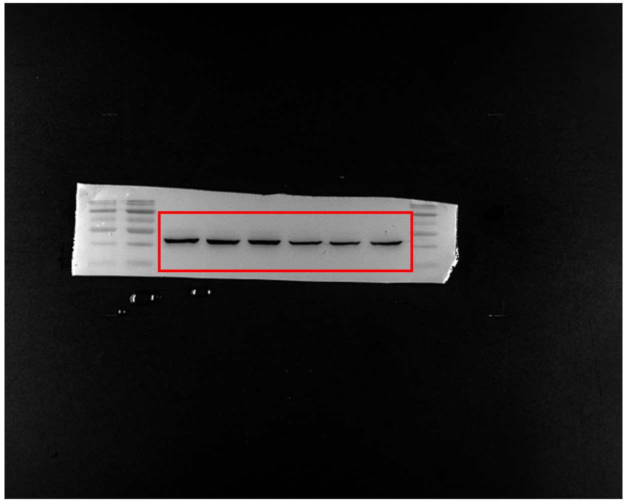

Fig 7A-hep-DLG-Fpn

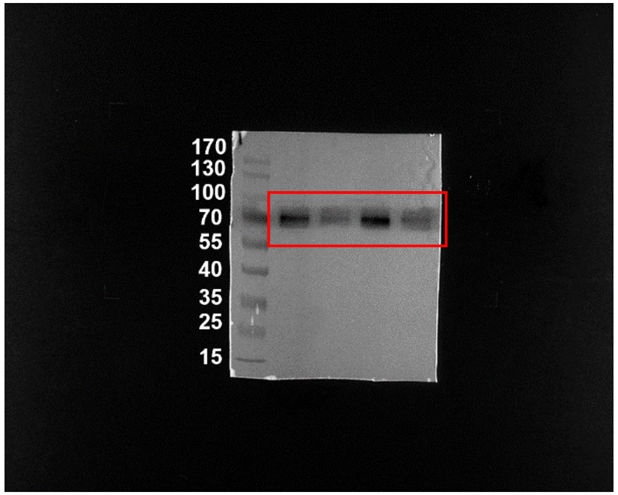

Fig 7F-hep-U0126-Fpn

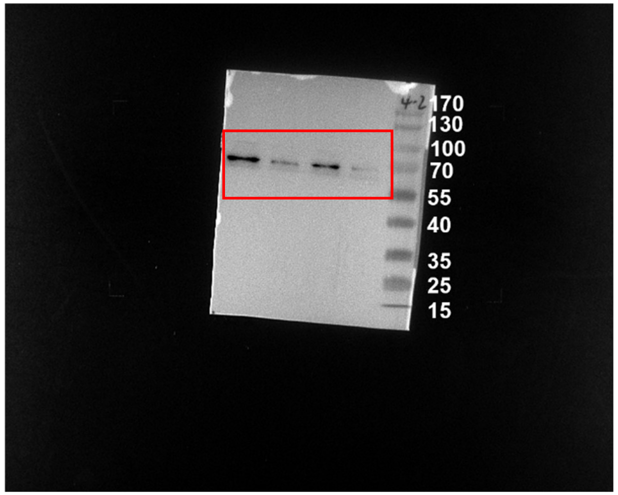

Fig 7-hep-TfR(right-A, left-F)

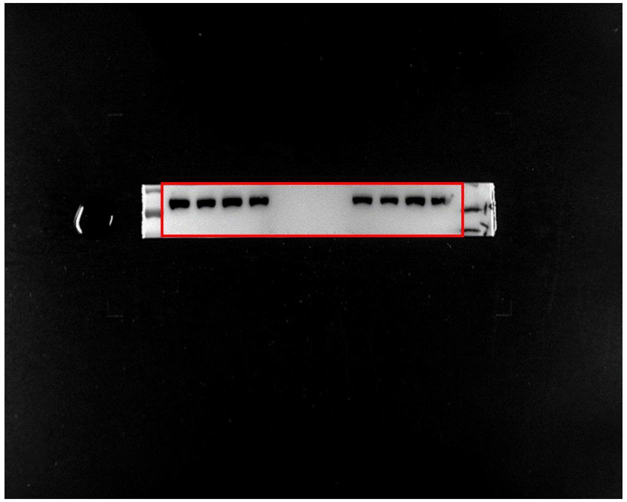

Fig 7-hep-FtL(right-A, left-F)

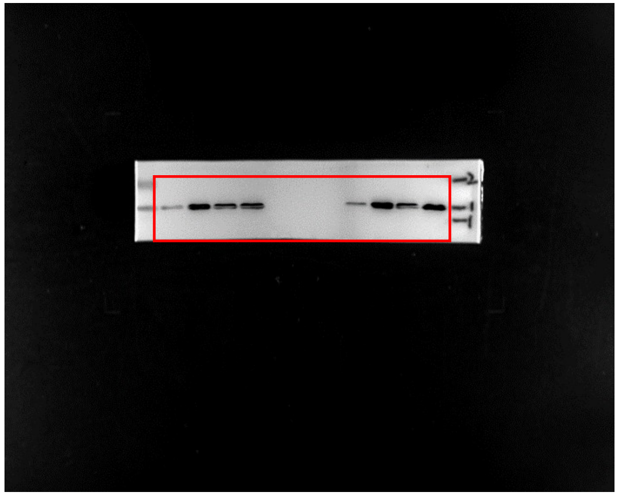

Fig 7-hep-pErK(right-A, left-F)

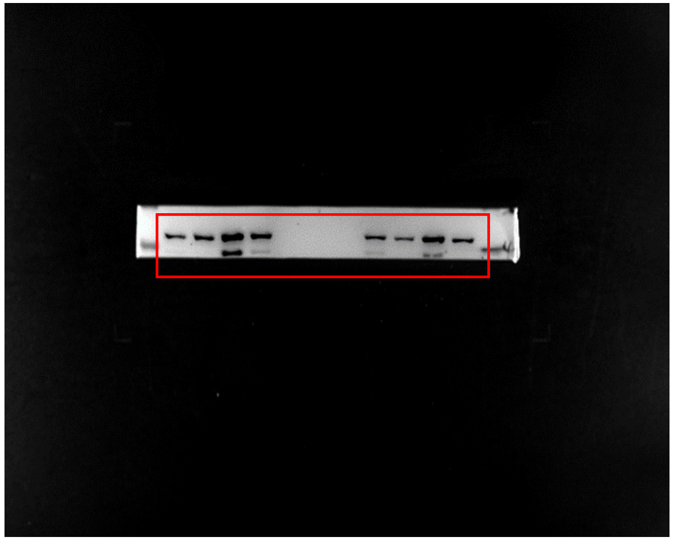

Fig 7-hep-ErK(right-A, left-F)

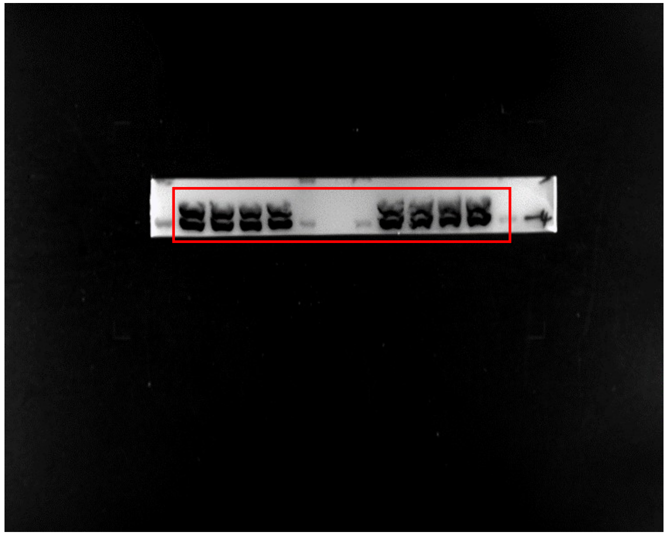

Fig 7-hep-actin(right-A, left-F)

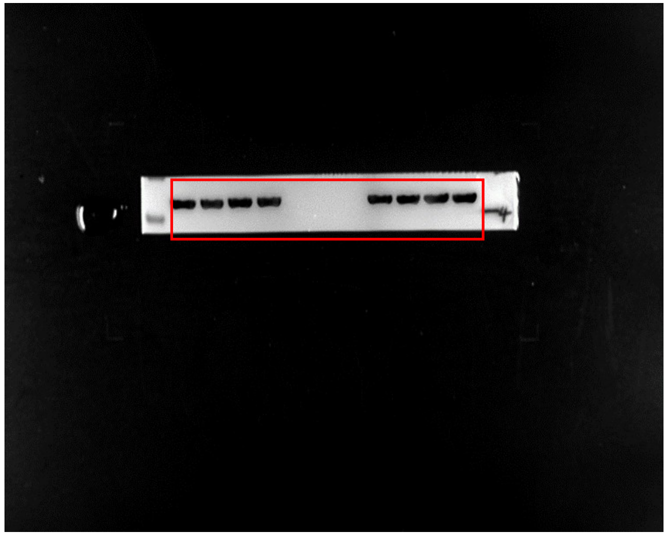

Fig 8-mac-Fpn(left-A, right-F)

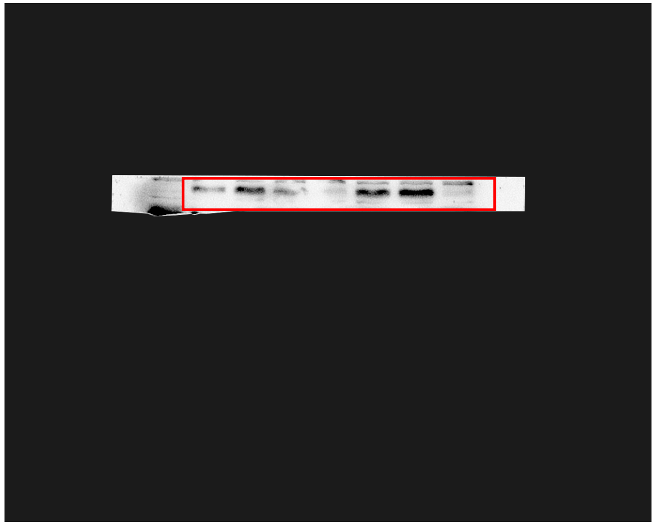

Fig 8-mac-TfR(left-A, right-F)

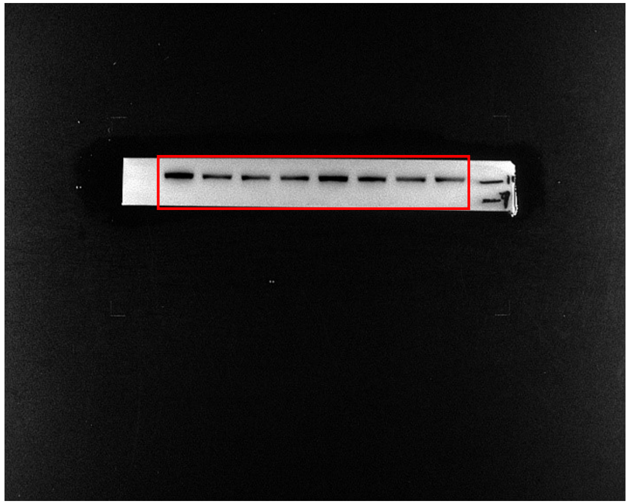

Fig 8-mac-FtL(left-A, right-F)

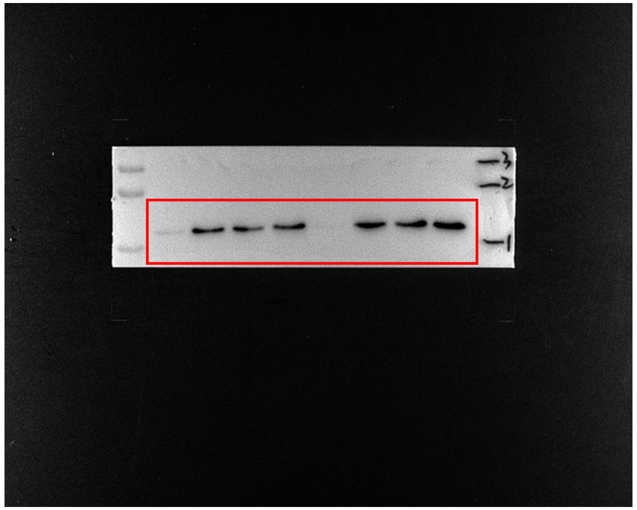

Fig 8-mac-pErK(left-A,right-F)

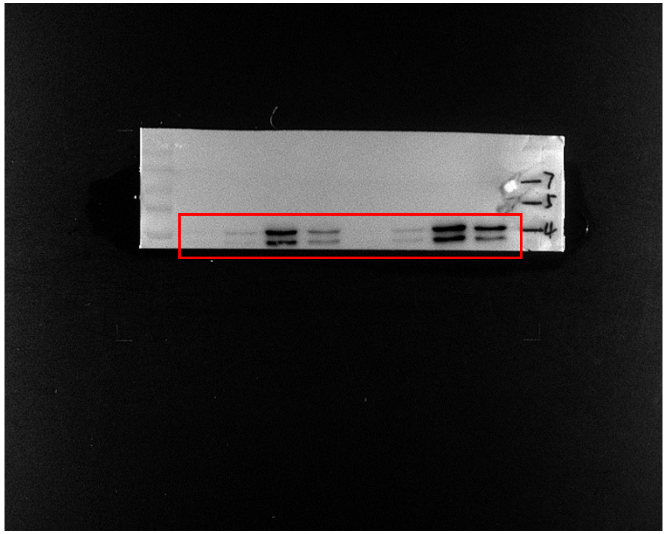

Fig 8-mac-ErK(left-A,right-F)

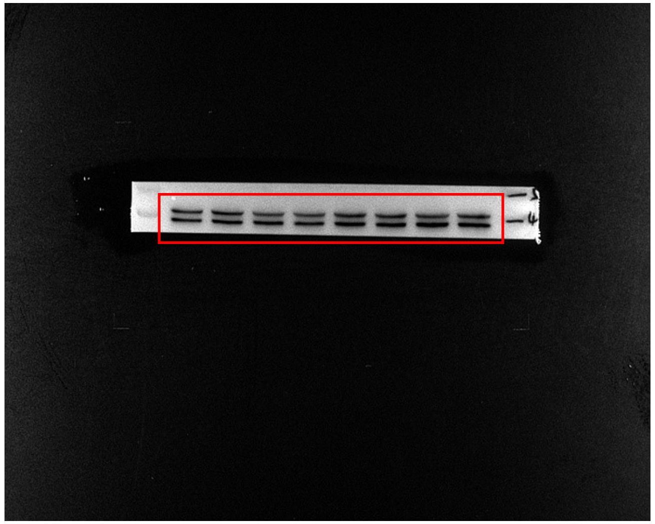

Fig 8-mac-actin (left-A,right-F)

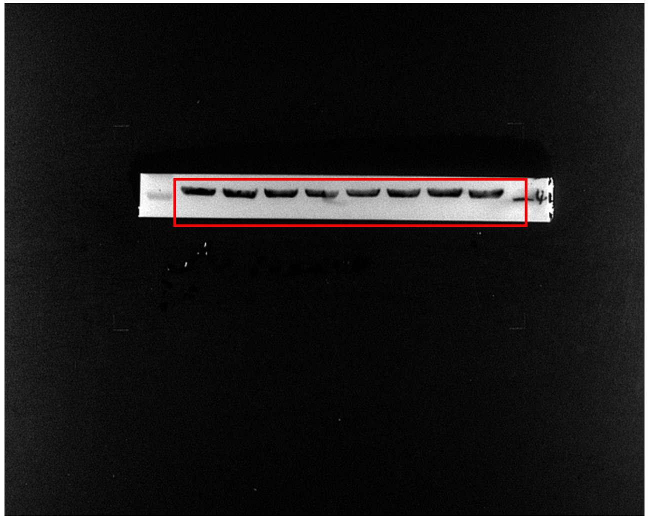

Others replicated blots

Fig 3-liver

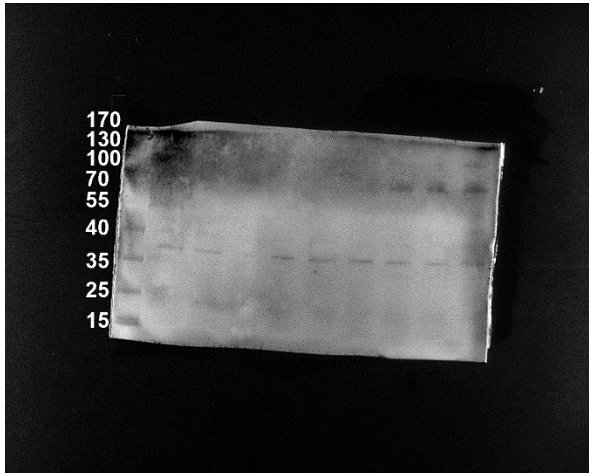

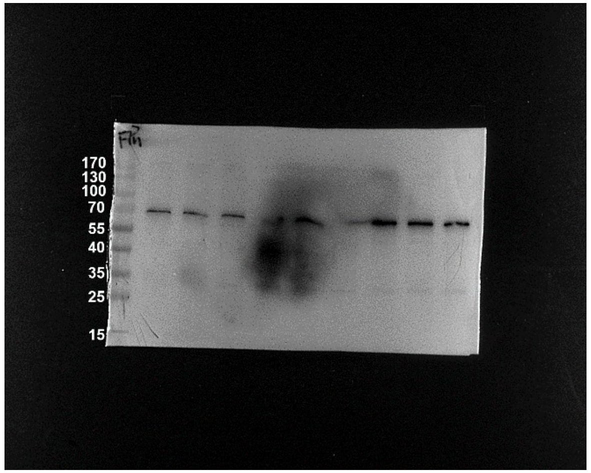

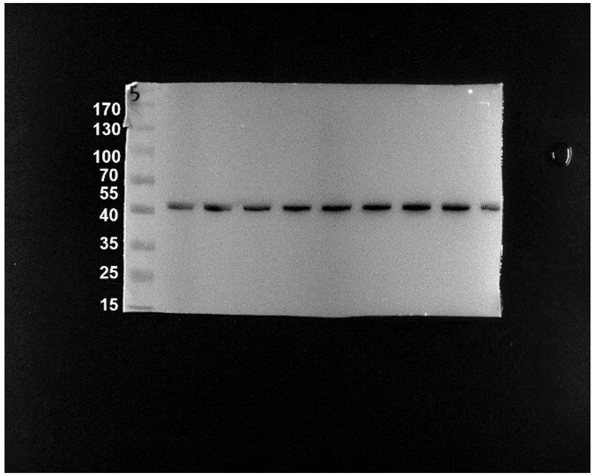

Fig 3-spleen

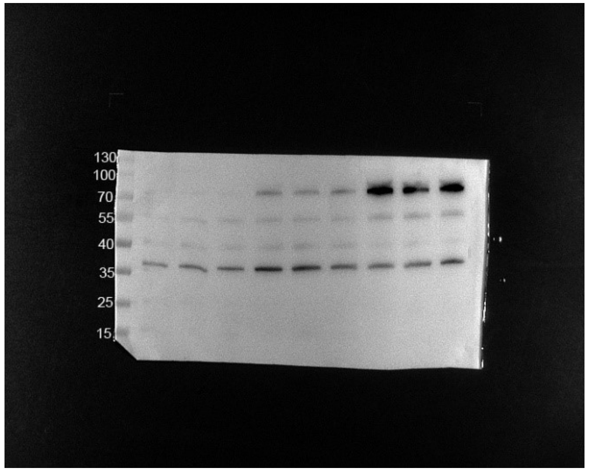

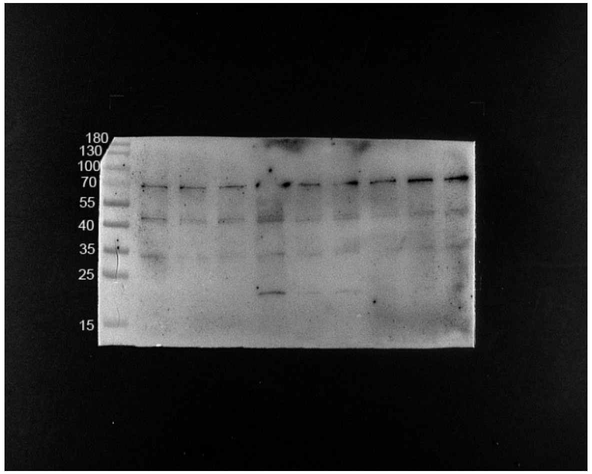

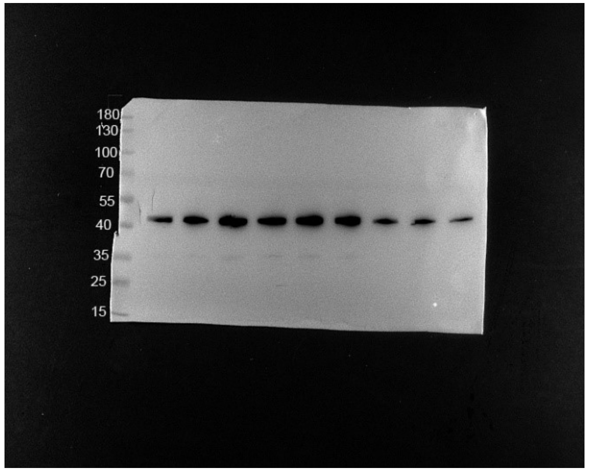

Fig 4A-liver

HH-Liver-Ft-L（Fig. 4A）

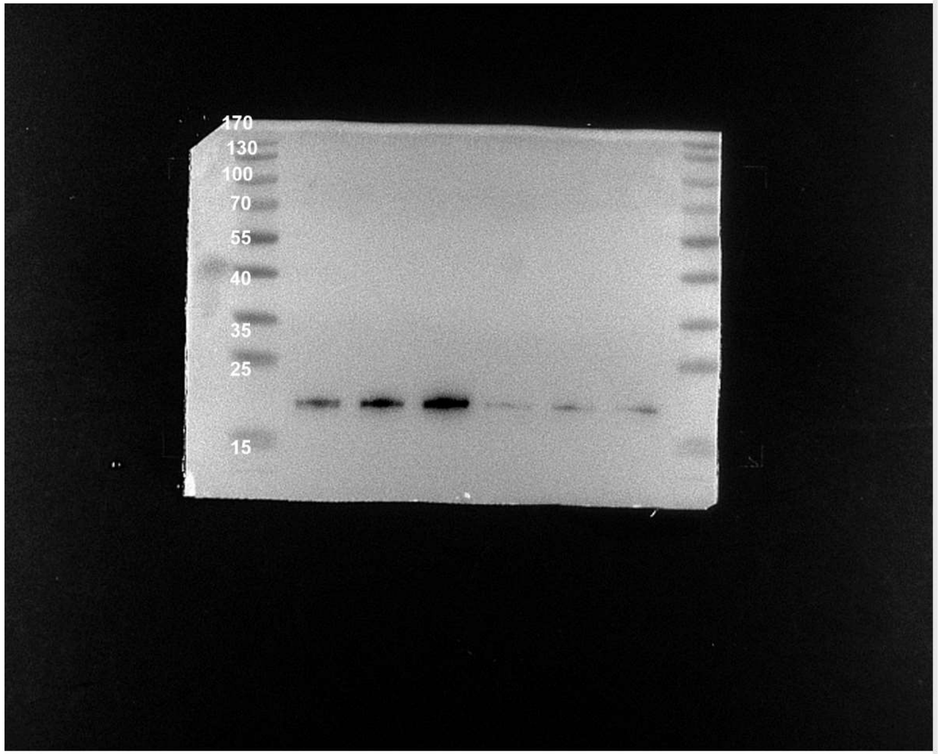

Figure 4A

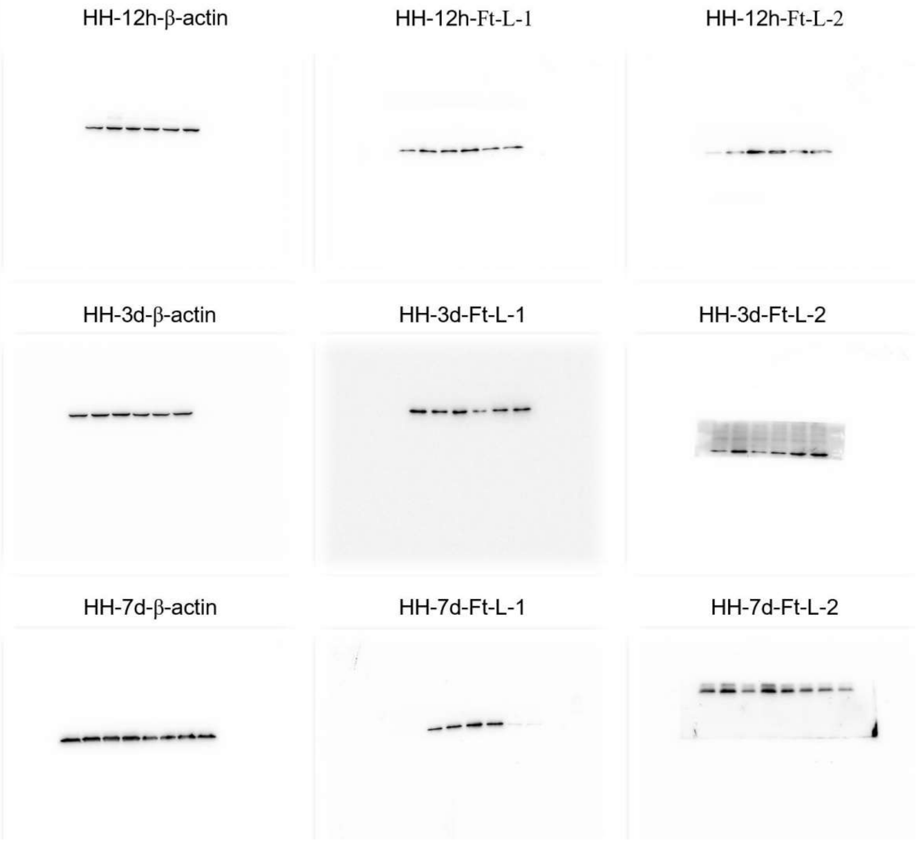

Fig 4K-spleen

HH-Spleen-Ft-L (Fig. 4K)

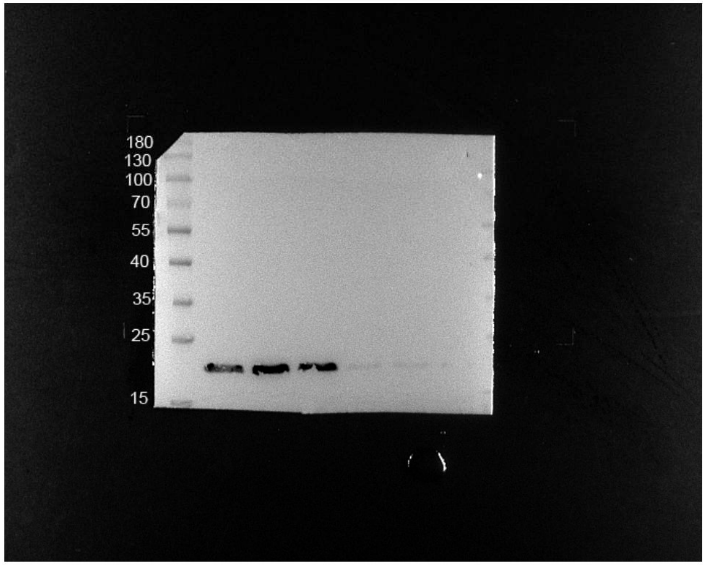

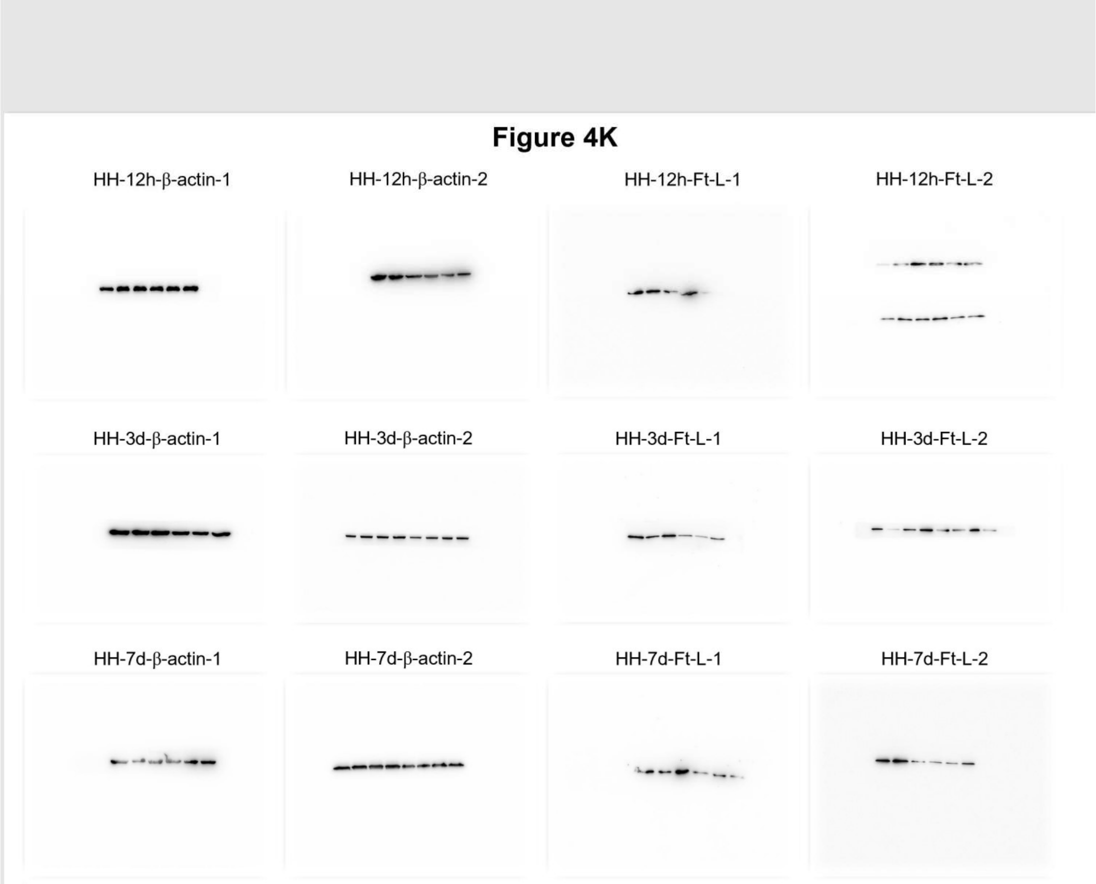

Fig 6-liver

HH-liver-TfR

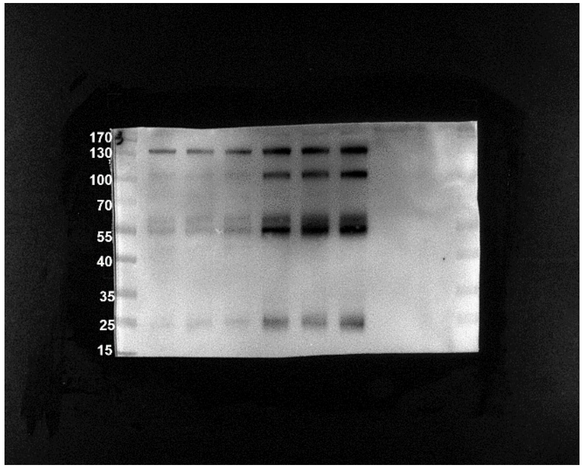

HH-Liver-actin

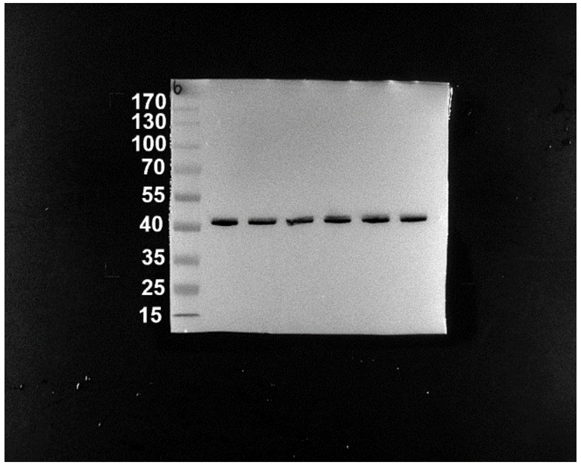

HH-liver-FtL

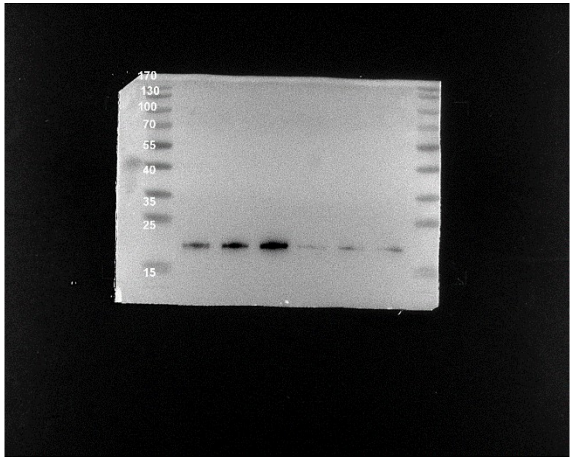

HH-Liver-Fpn (Fig. 6A)

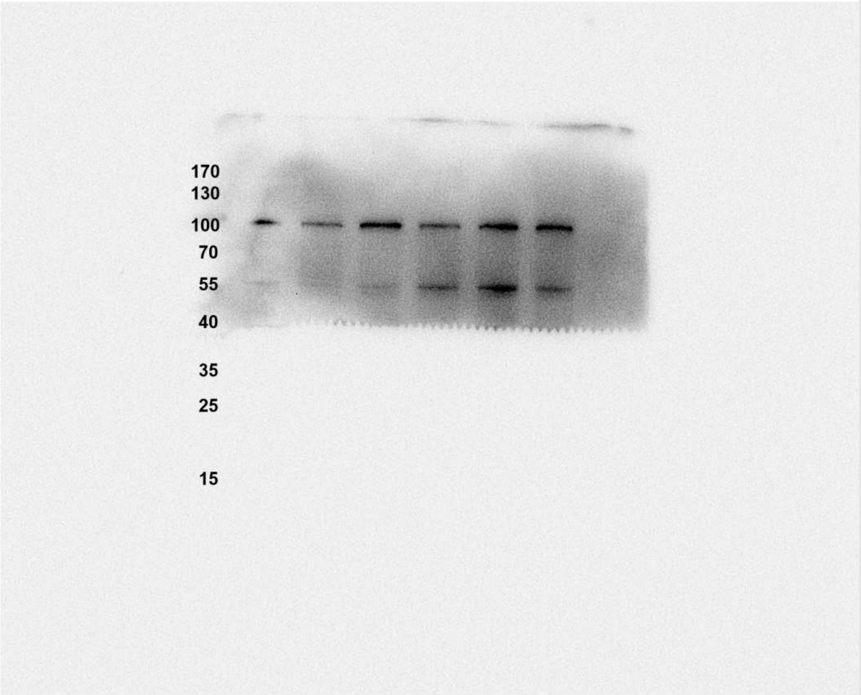

Figure 6A

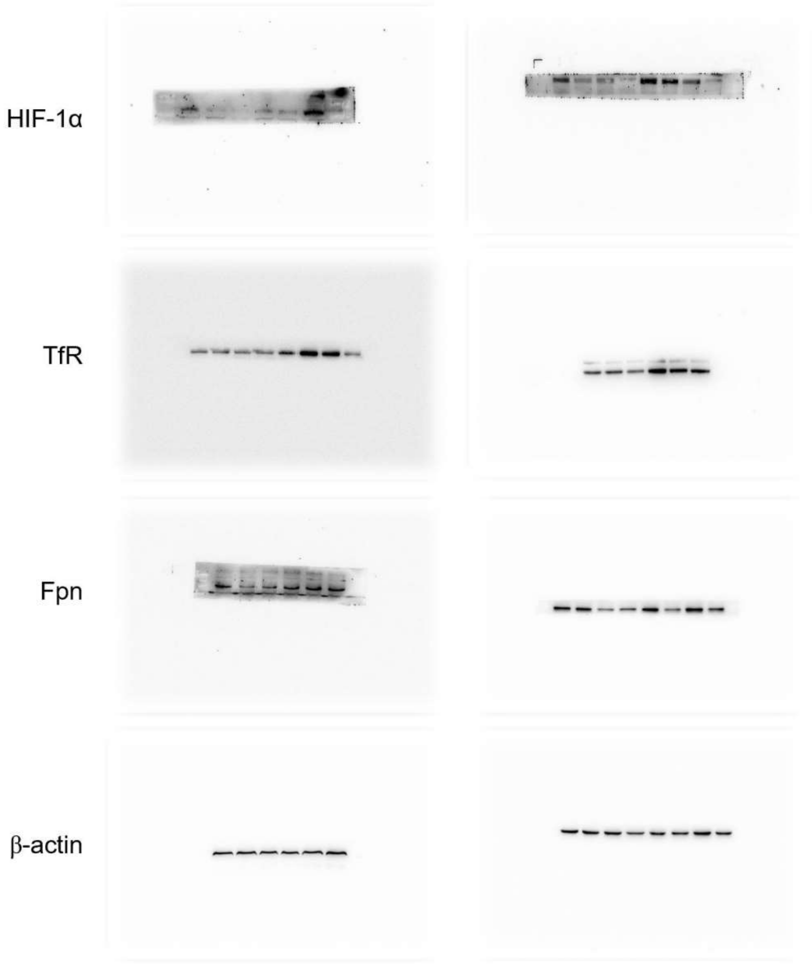

HH-Spleen-beta-actin（Fig. 6F）

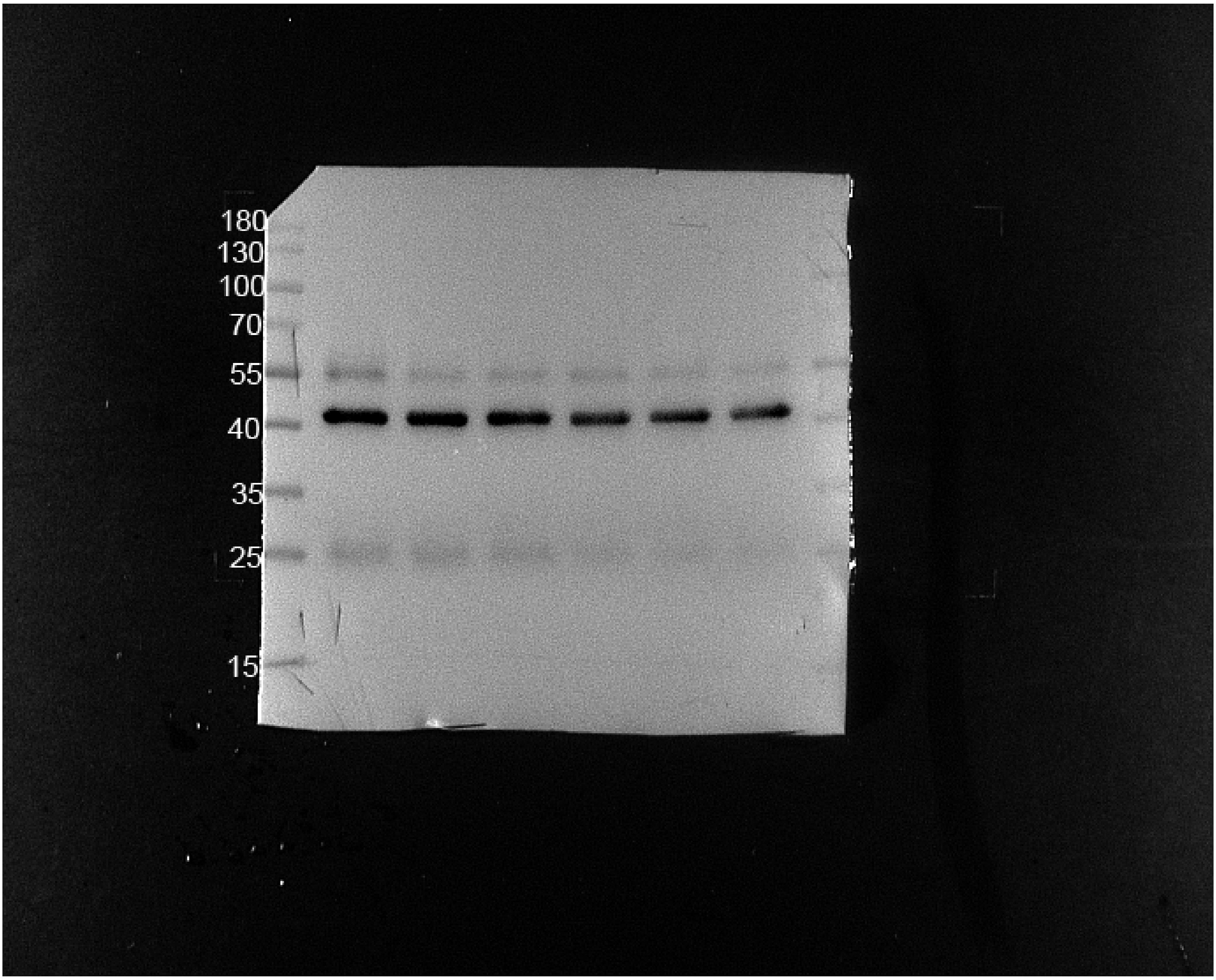

HH-Spleen-Fpn（Fig. 6F）

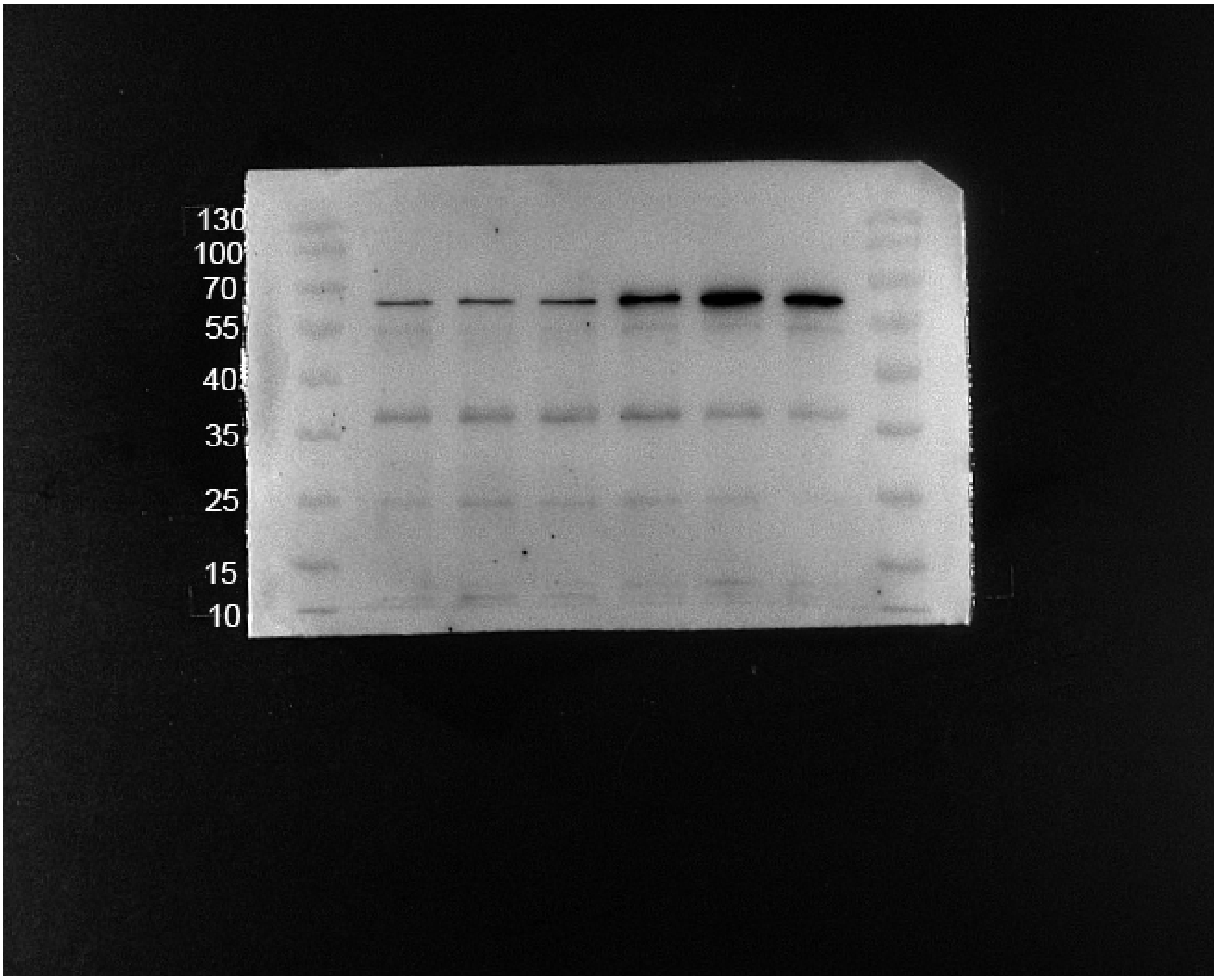

HH-Spleen-TfR（Fig. 6F）

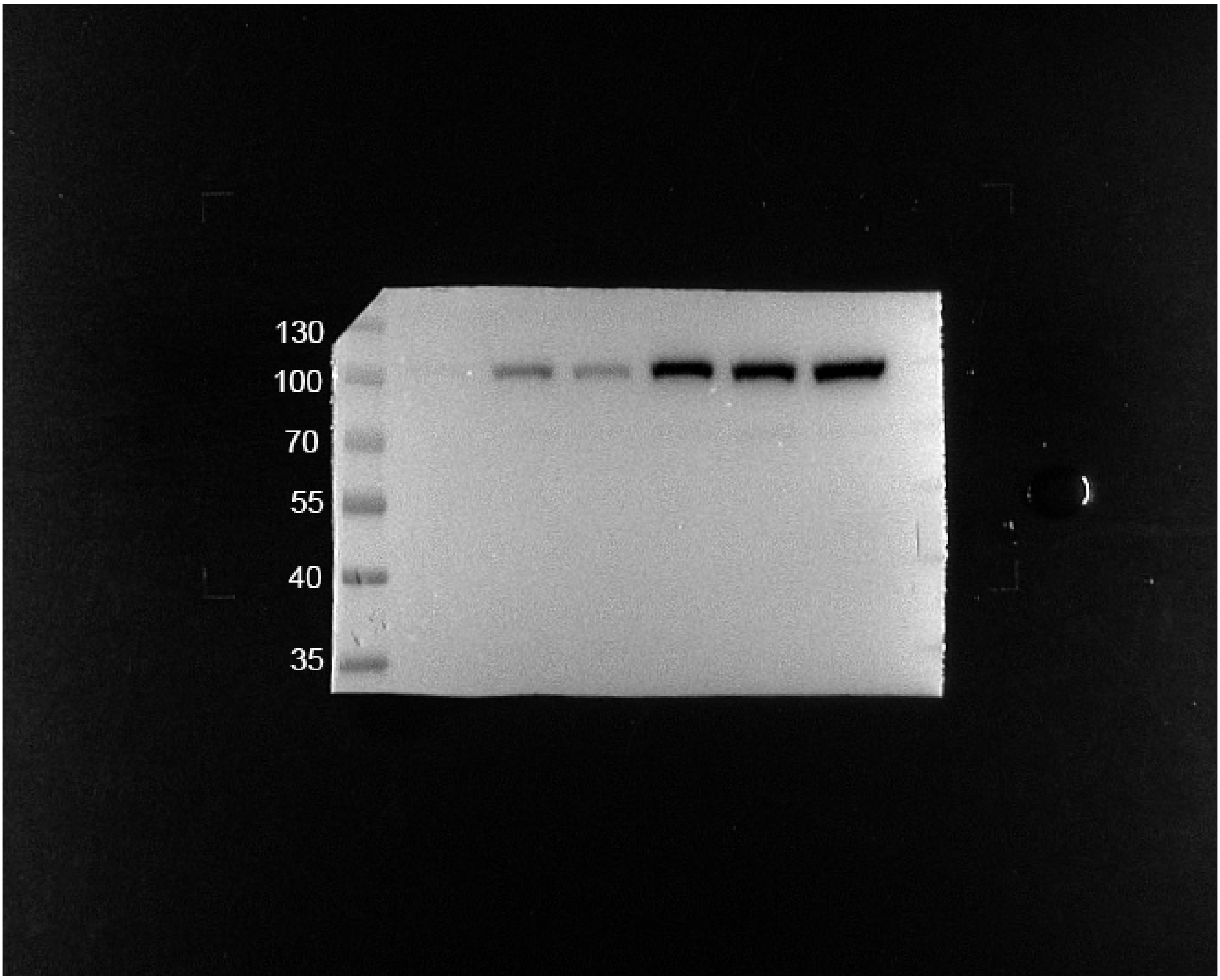

Figure 6F

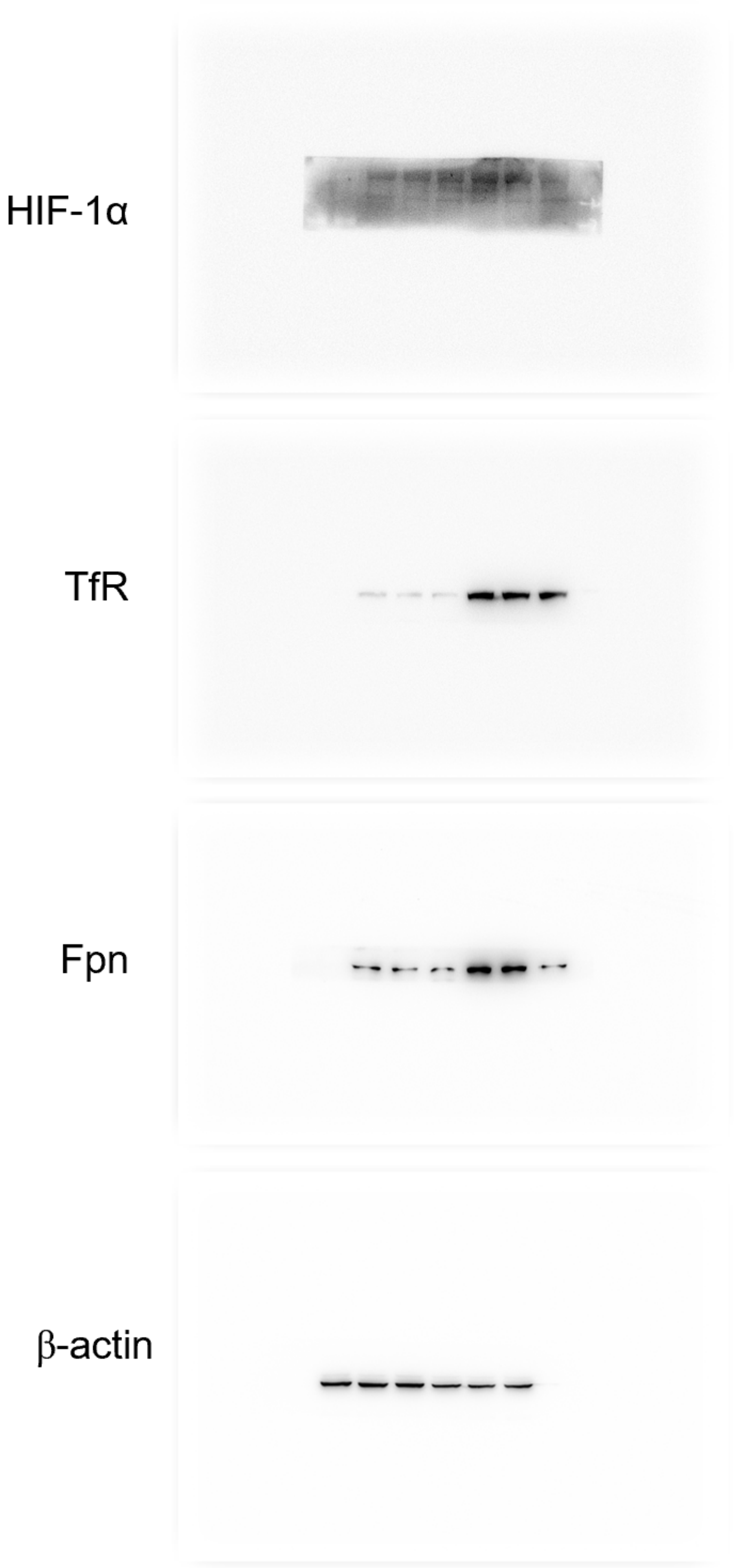

Hyp-Hep-beta-actin（Fig. 7A）

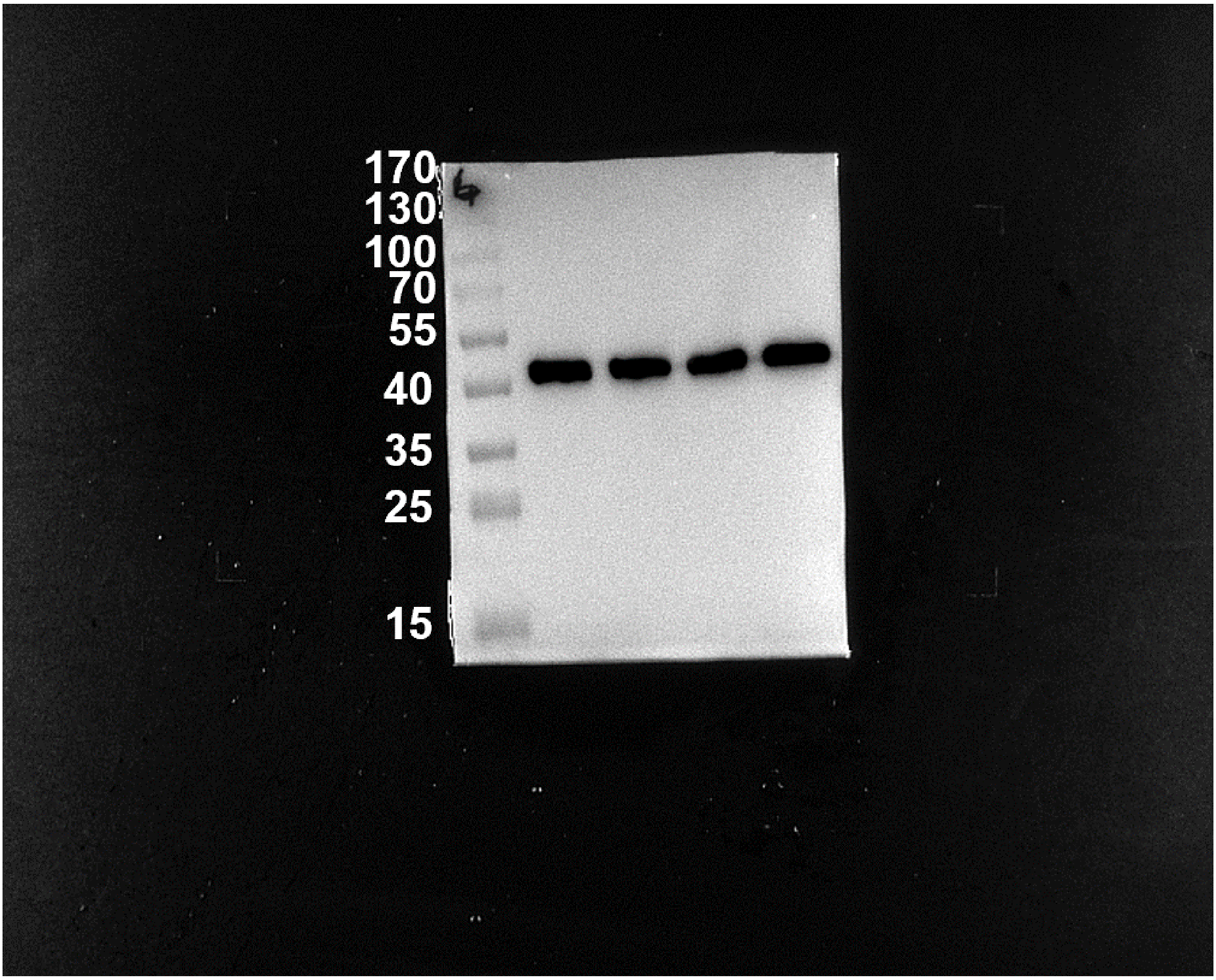

Hyp-Hep-p-Erk (Fig. 7A)

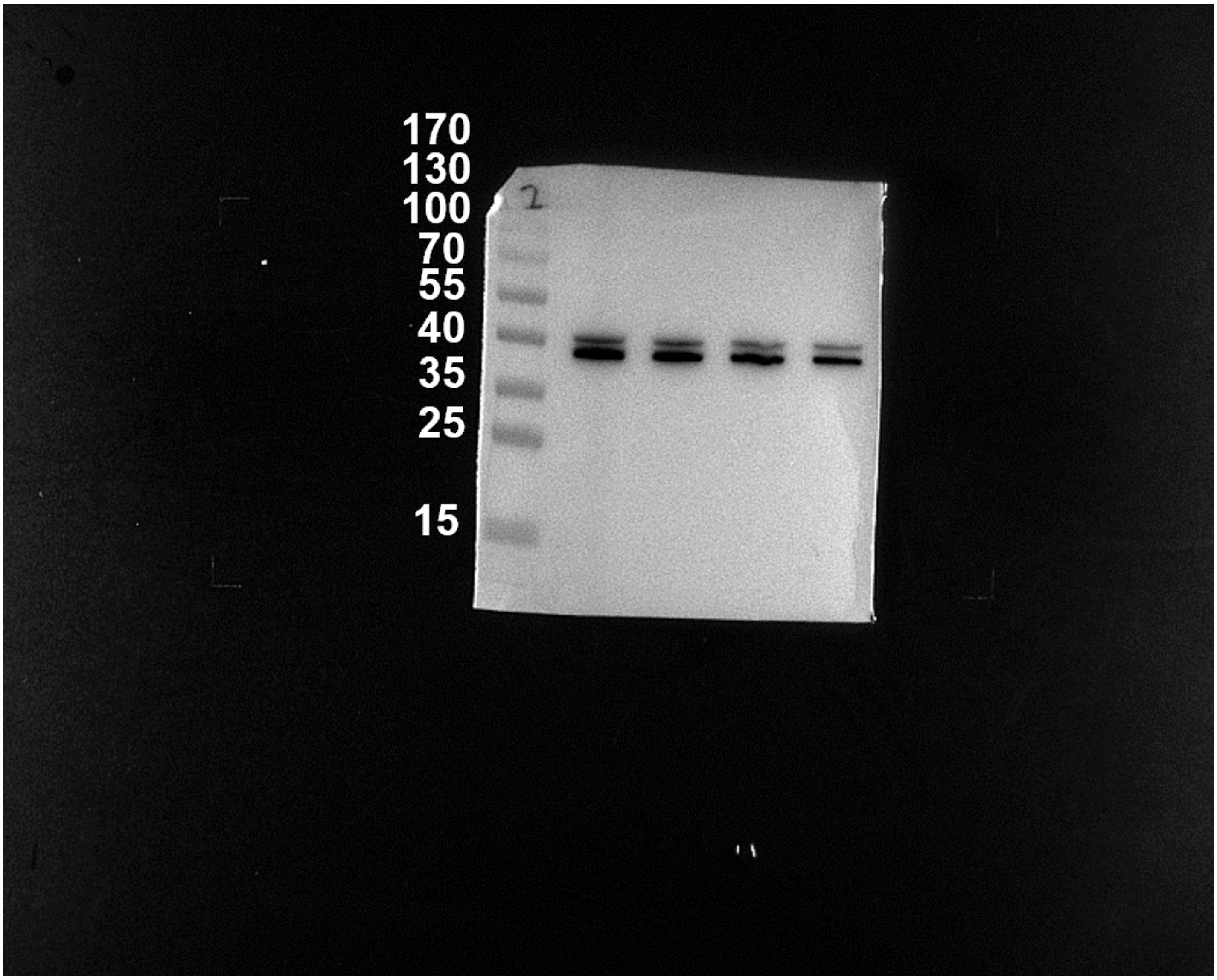

Hyp-Hep-Fpn (Fig. 7A)

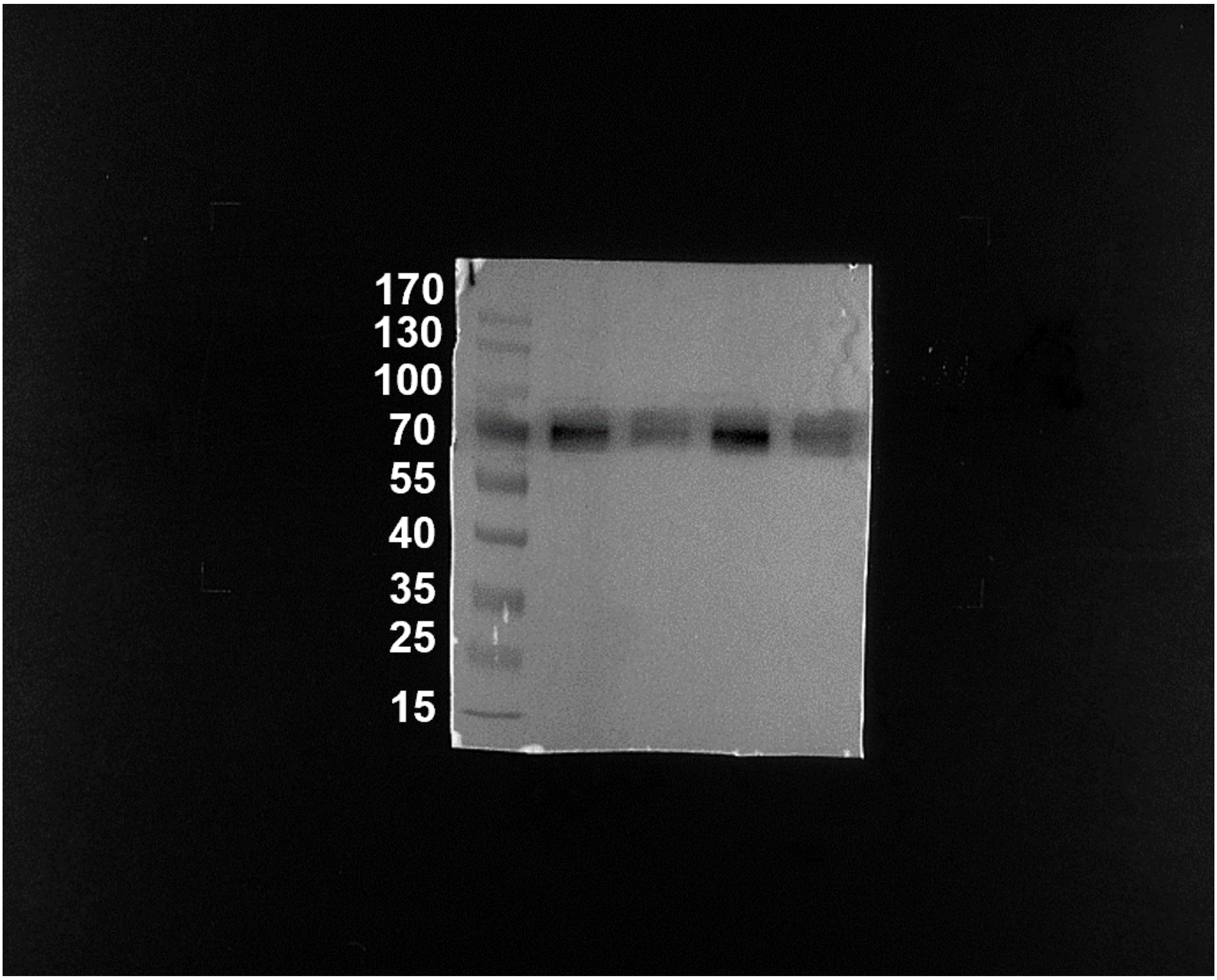

Hyp-Hep-FtL (Fig. 7A)

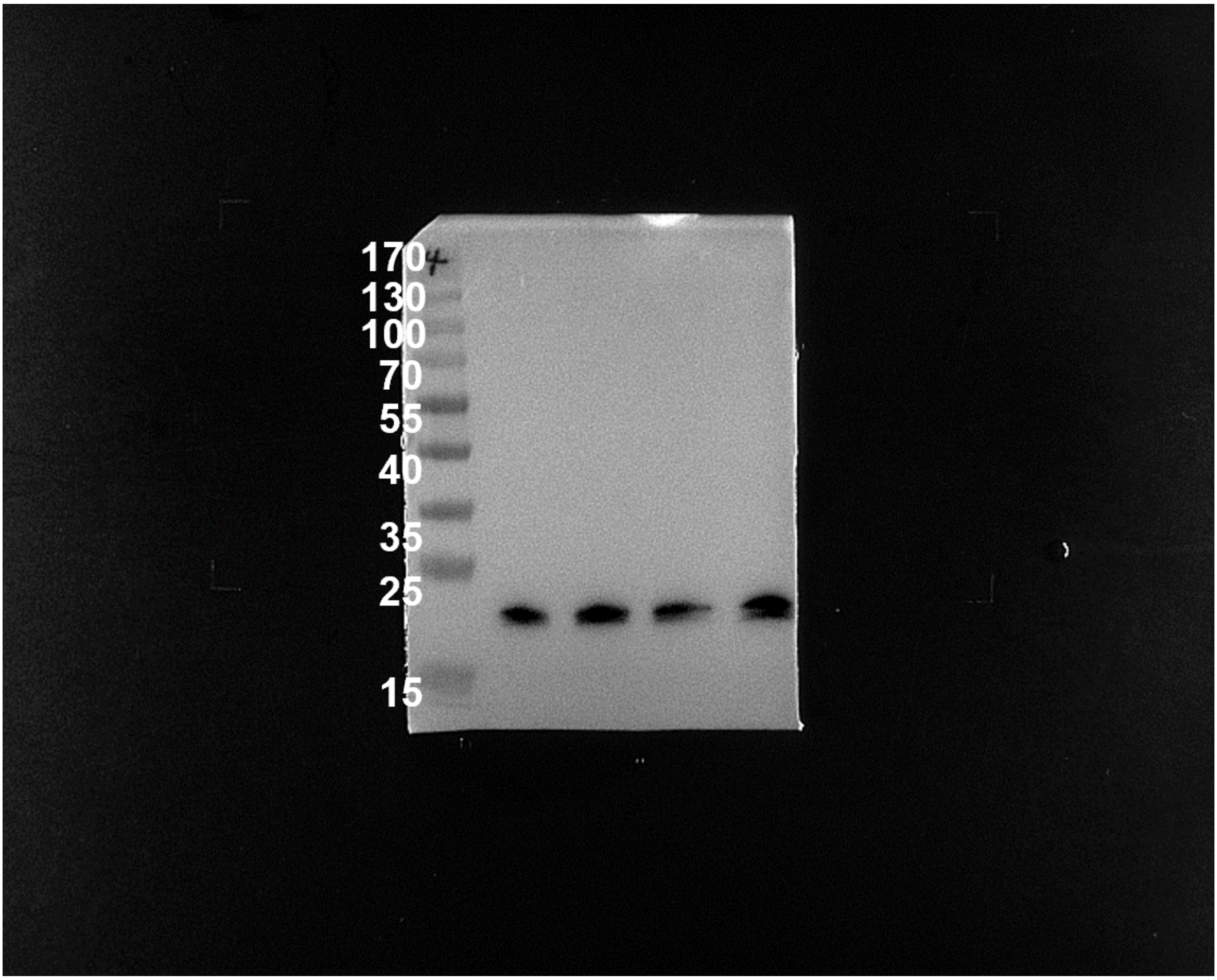

Hyp-Hep-Erk (Fig. 7A)

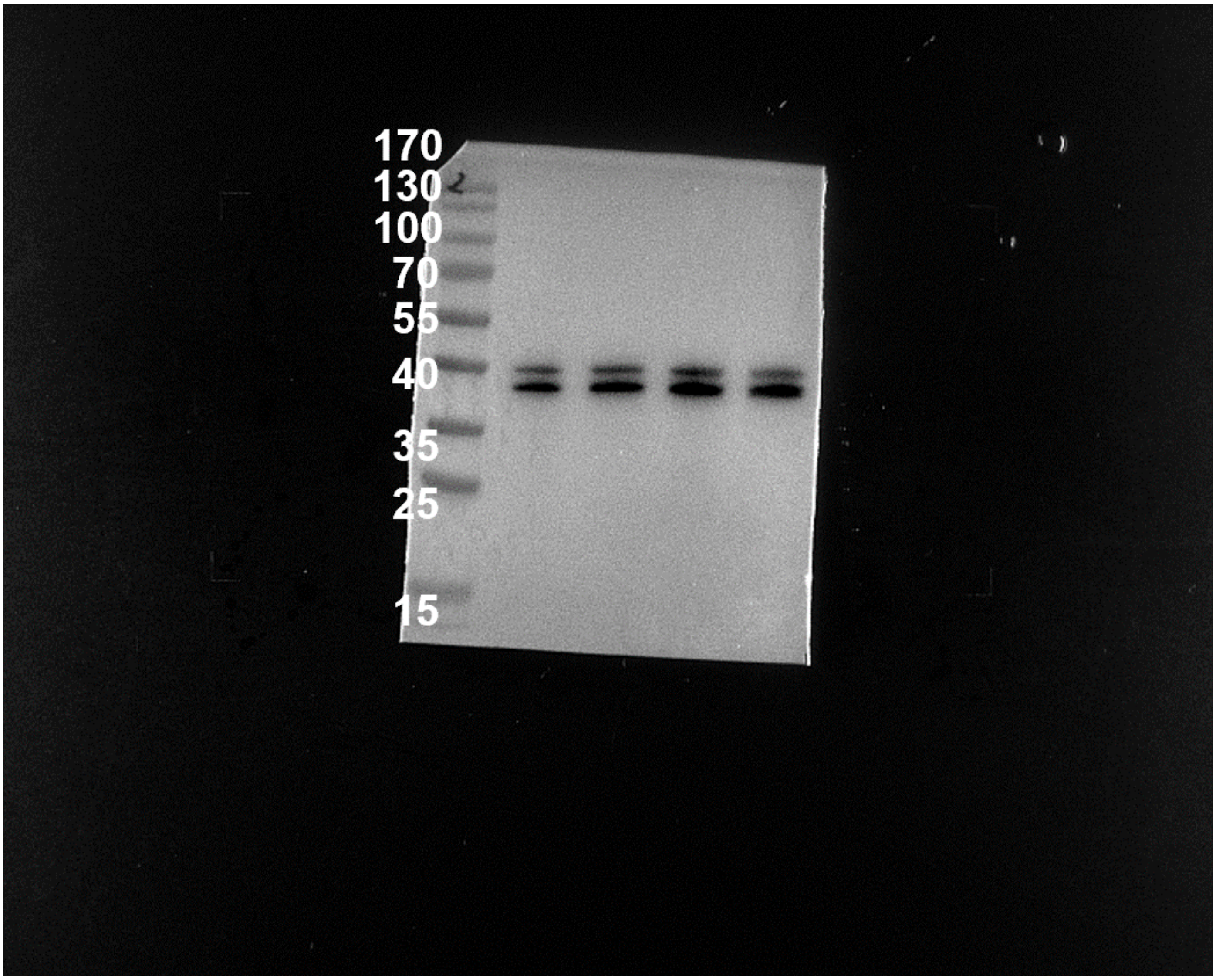

Hyp-Hep-GSHR-TfR (Fig. 7A)

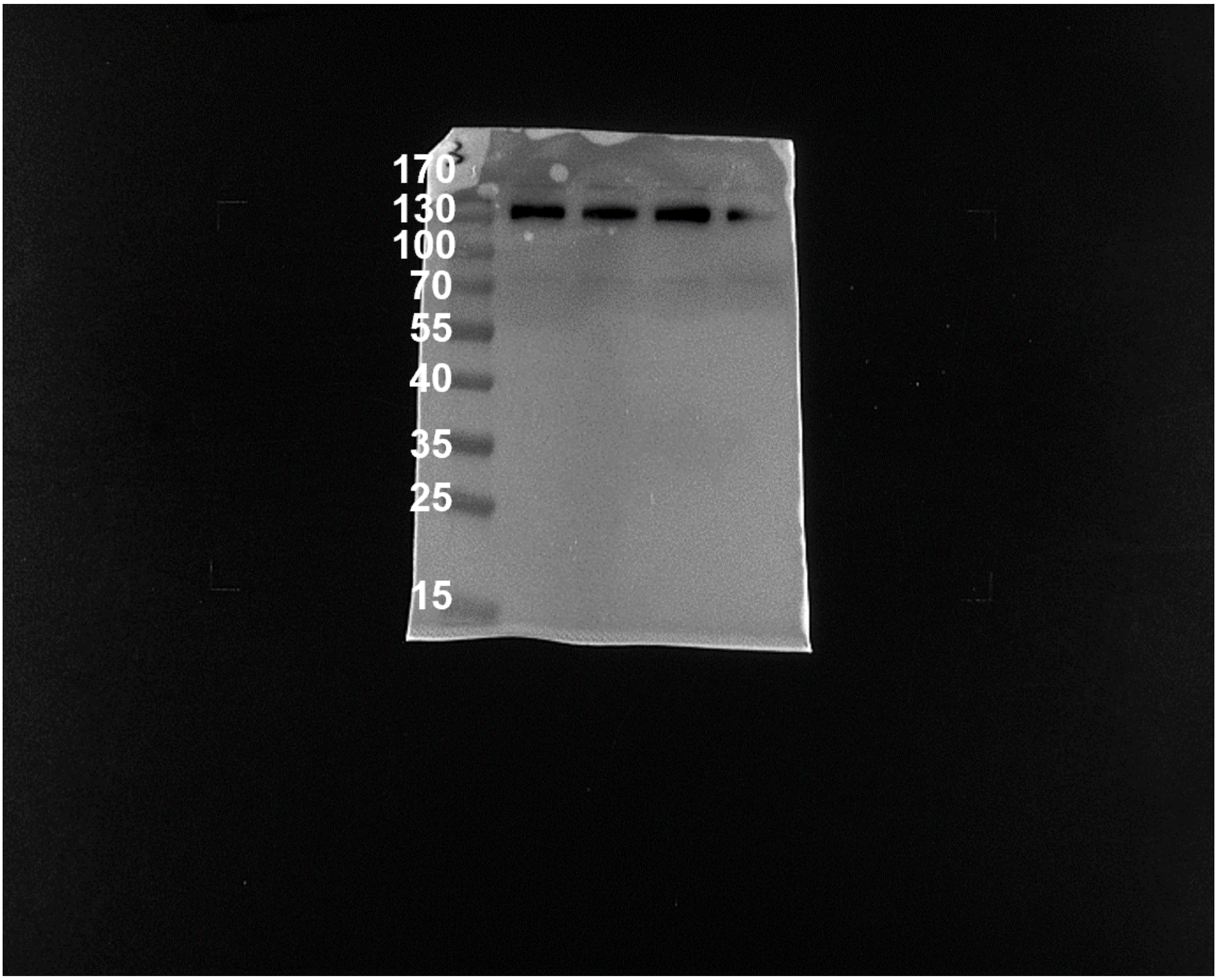

Hyp-Hep-U0126-beta-actin (Fig. 7F)

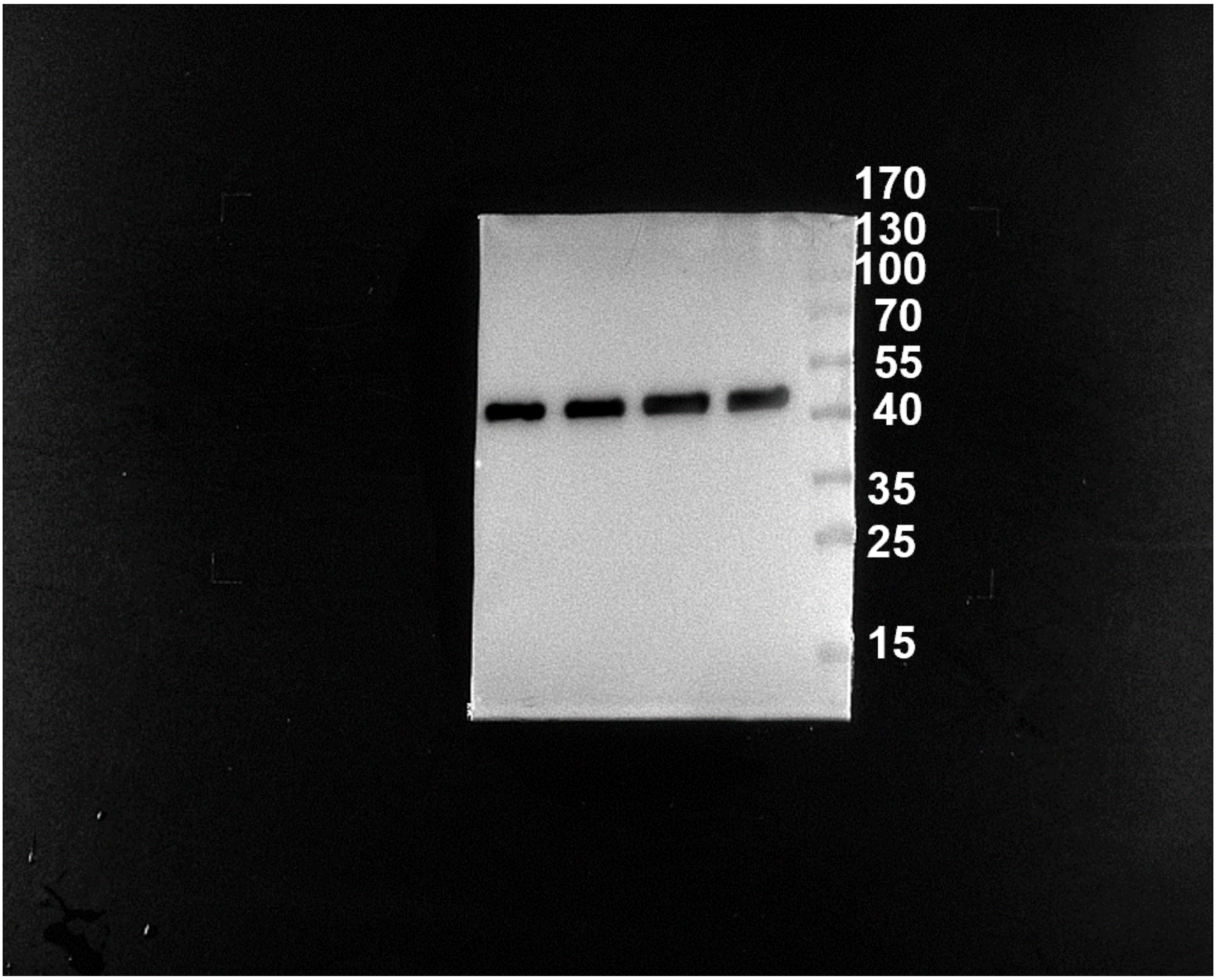

Hyp-Hep-U0126-Erk (Fig. 7F)

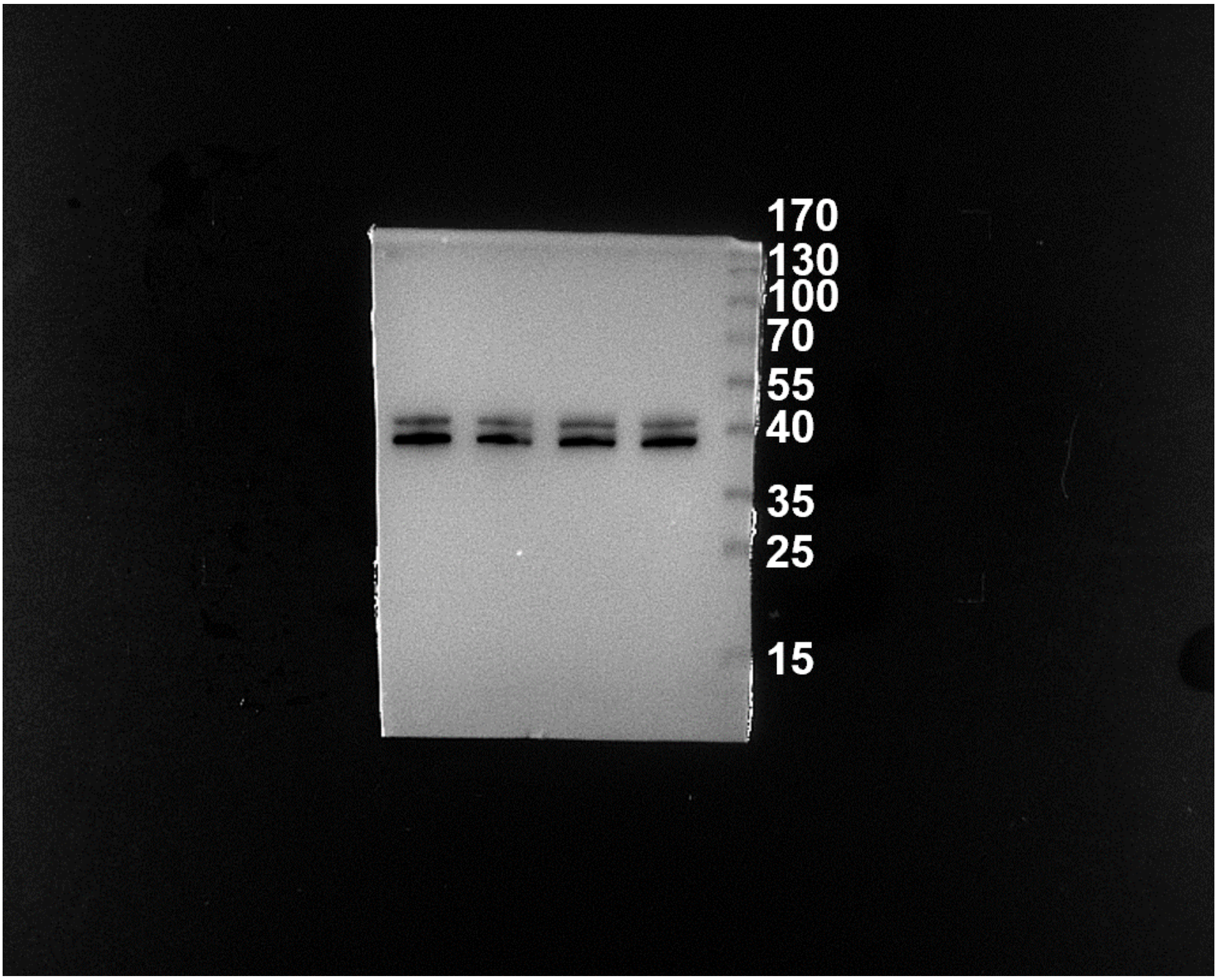

Hyp-Hep-U0126-Fpn (Fig. 7F)

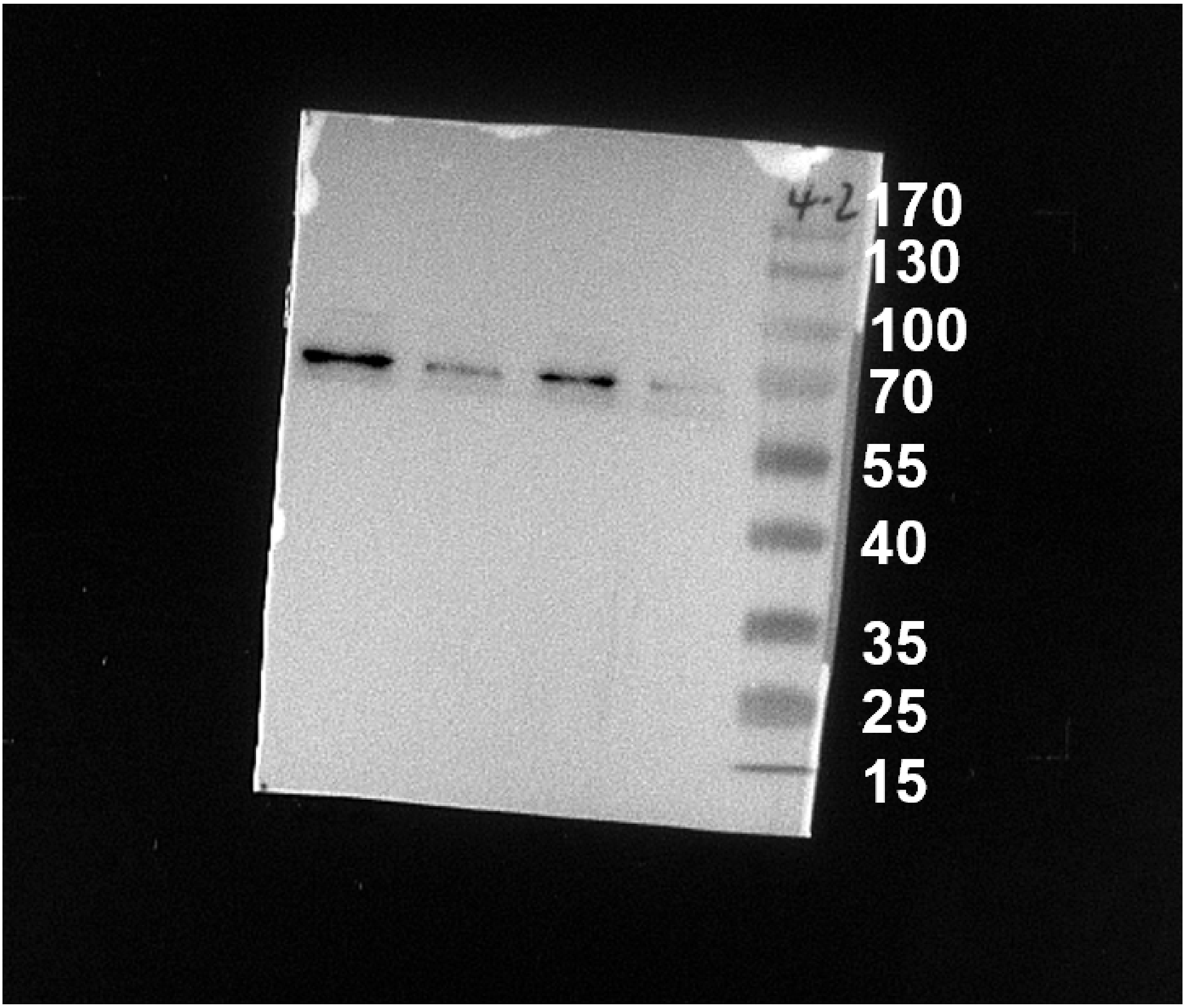

Hyp-Hep-U0126-FtL (Fig. 7F)

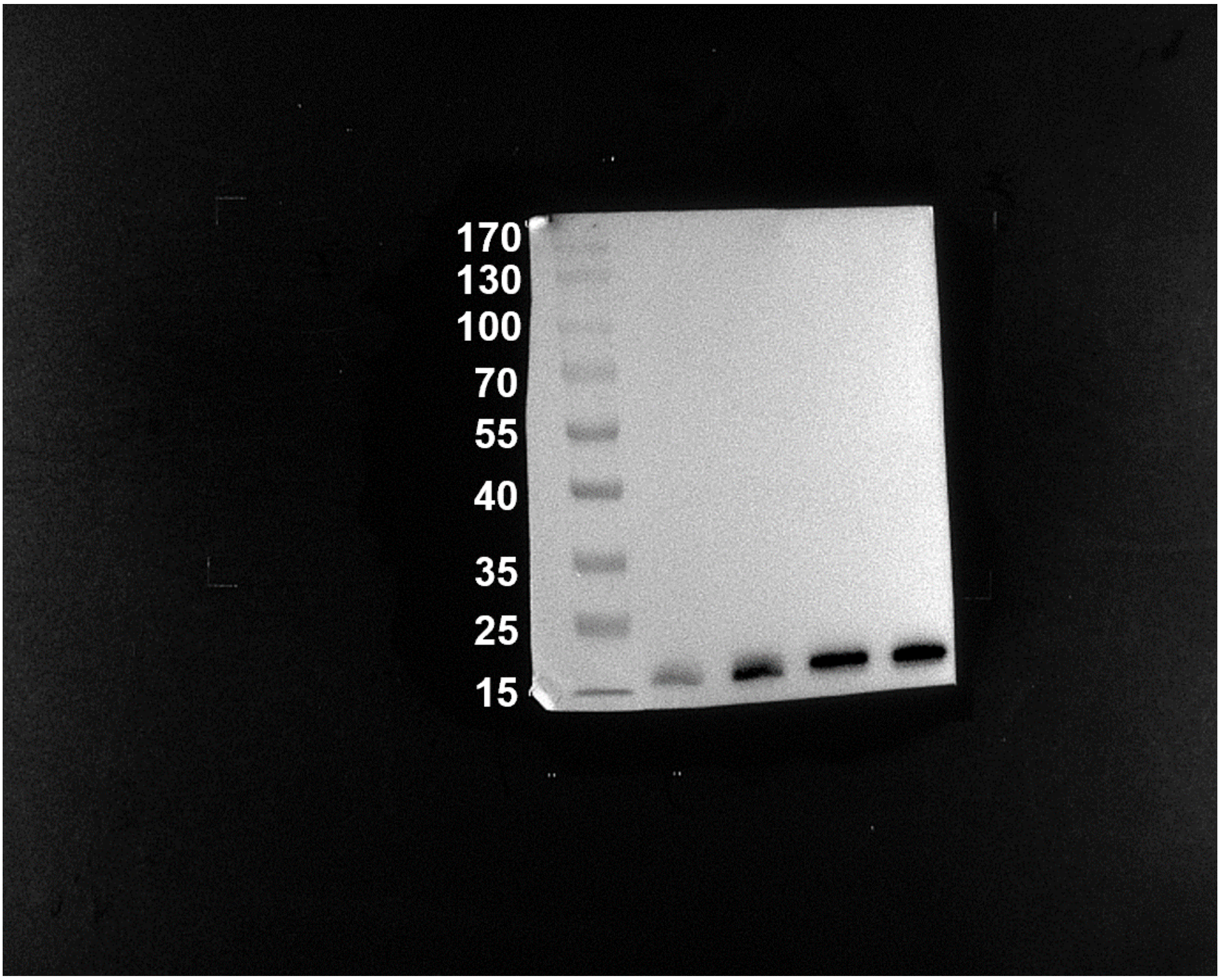

Hyp-Hep-U0126-pErk (Fig. 7F)

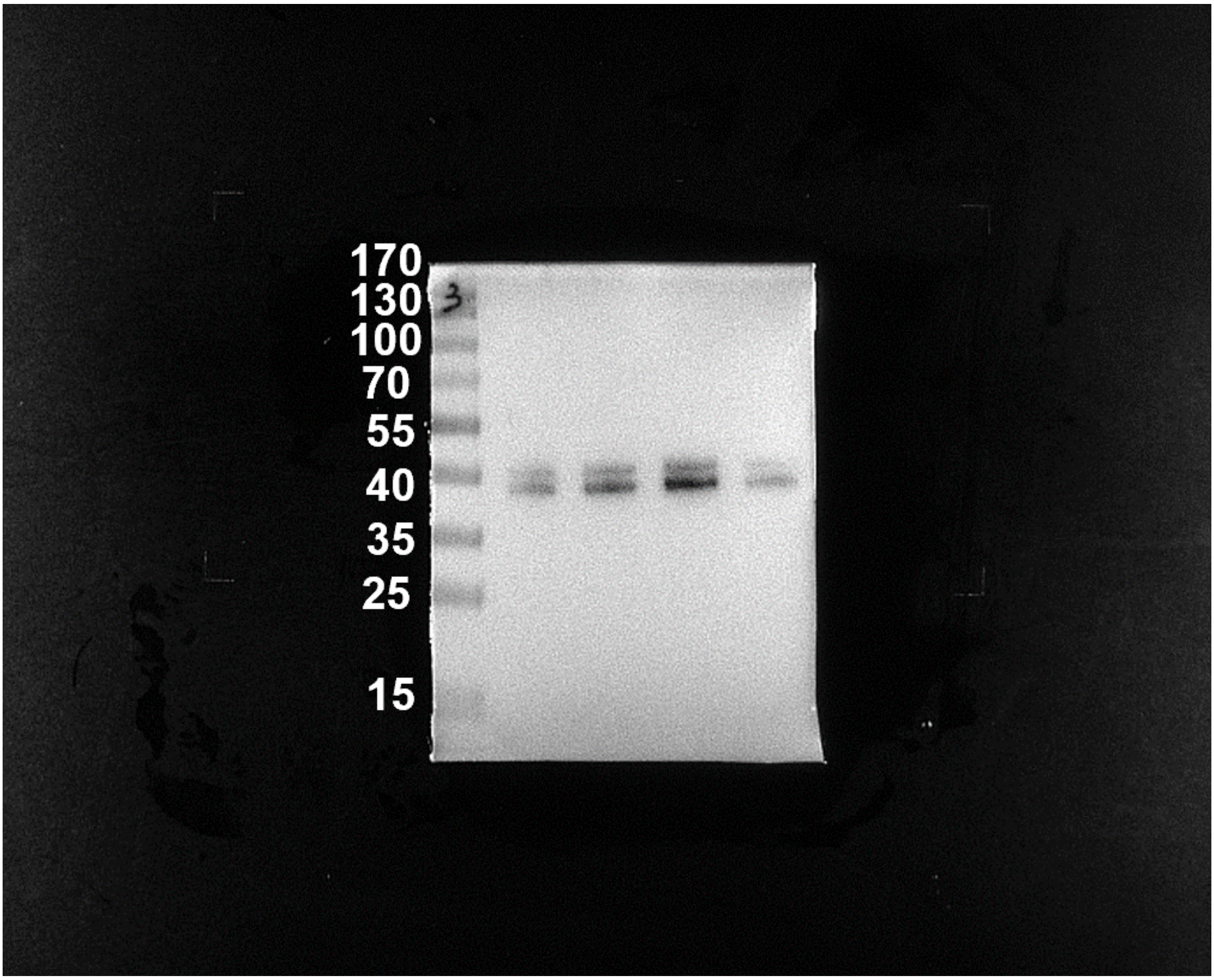

Hyp-Hep-U0126-TfR (Fig. 7F)

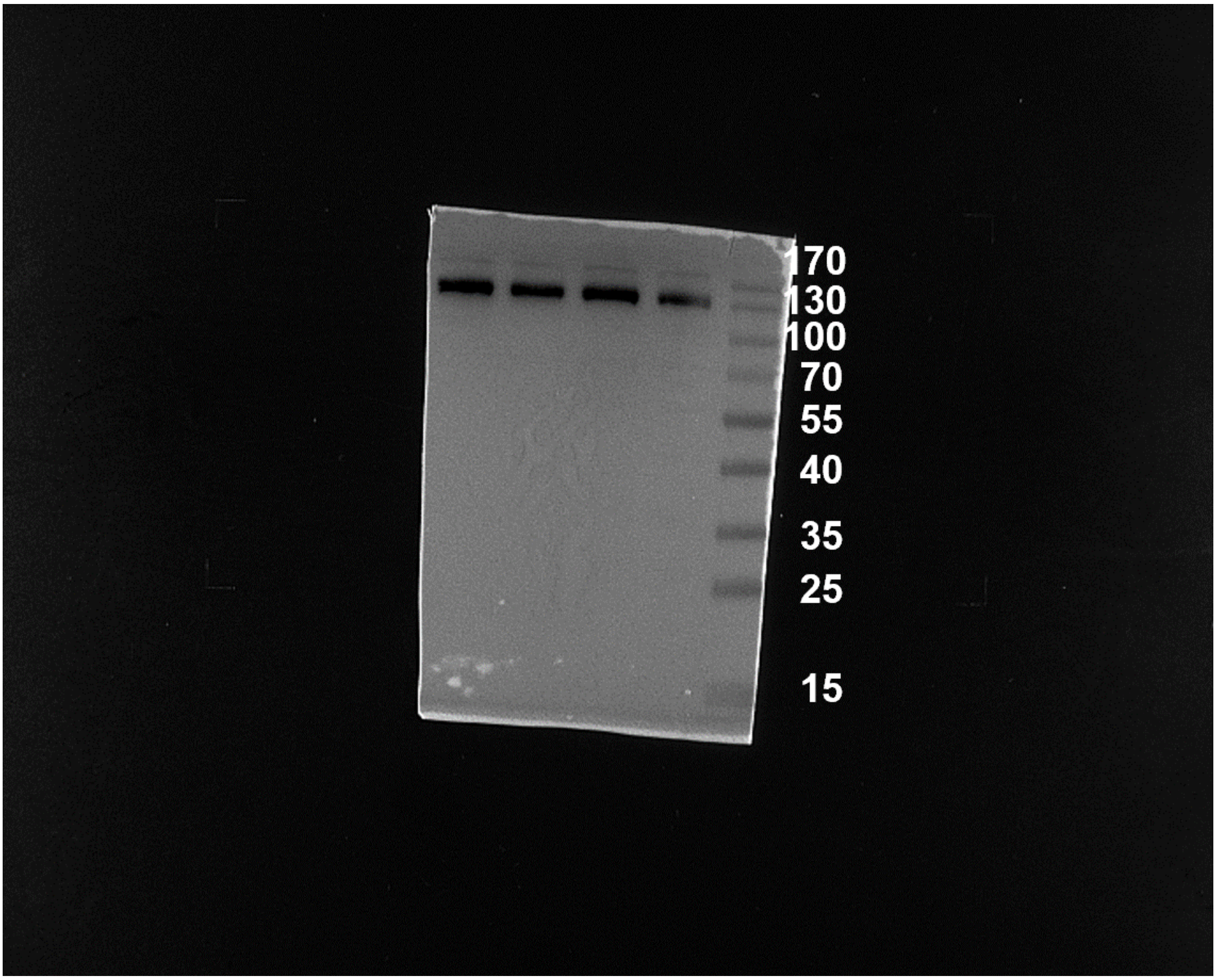

Figure 7A (left 4 lanes of each blot); Figure 7F (right 4 lanes of each blot)

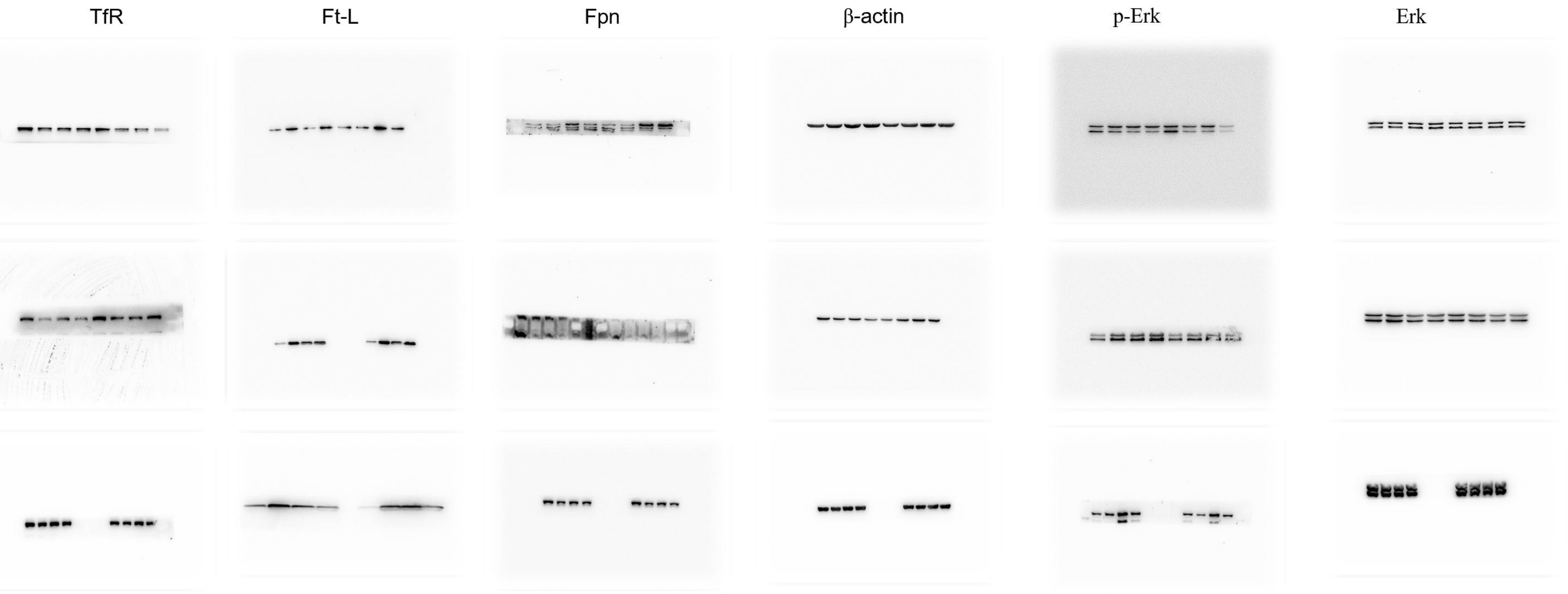

Hyp-PMC-beta-actin (Fig. 8A)

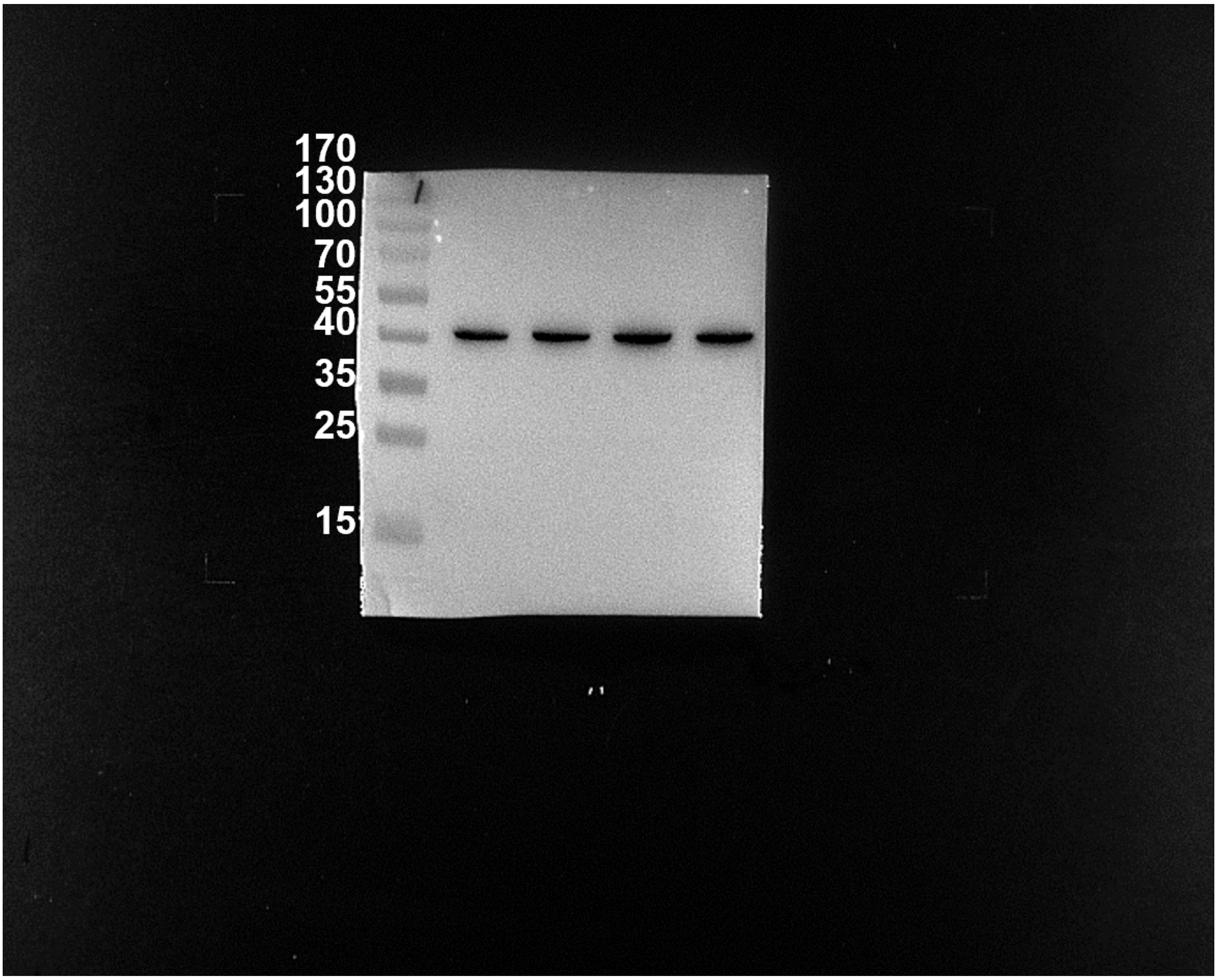

Hyp-PMC-GSHR-Erk (Fig. 8A)

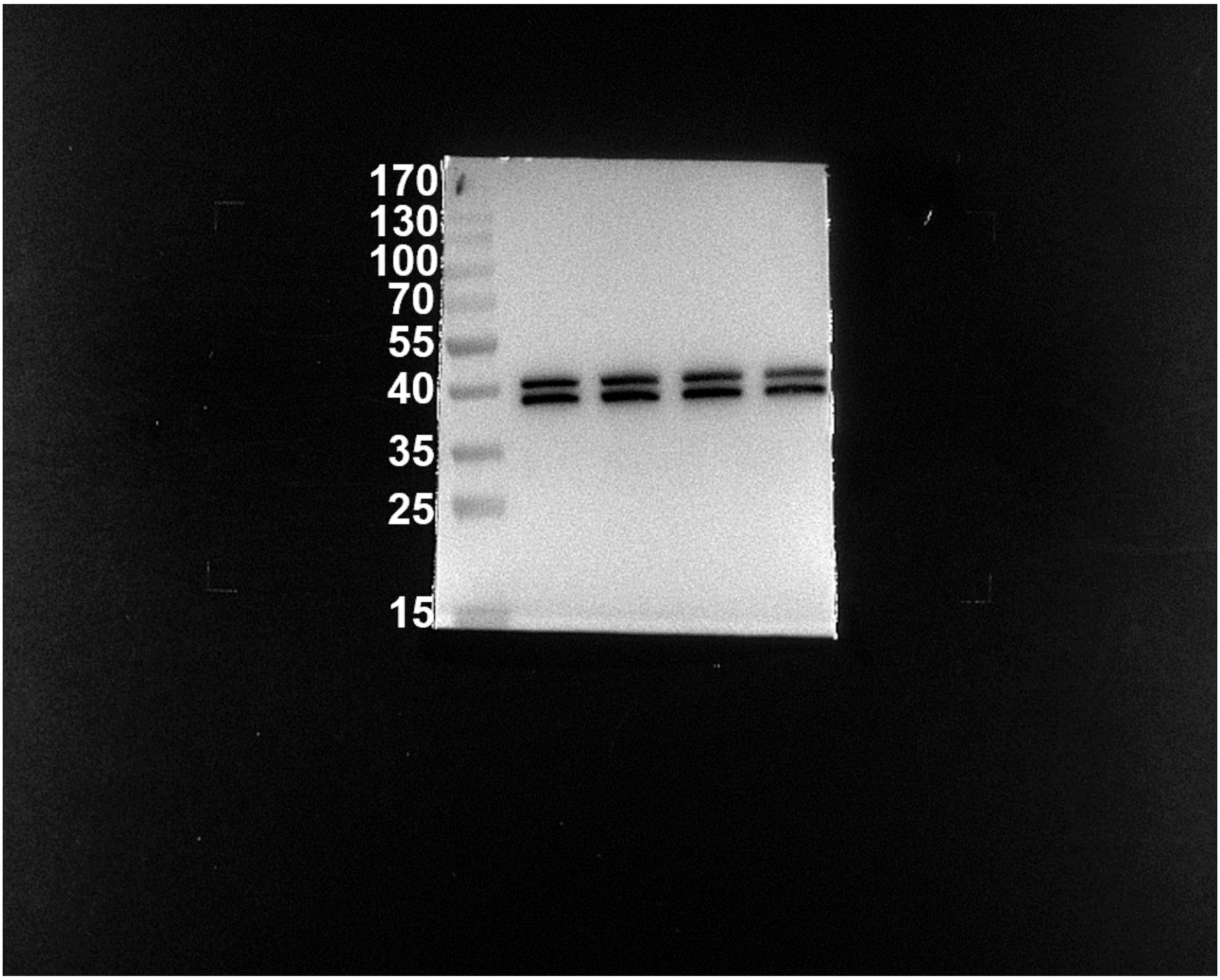

Hyp-PMC-GSHR-Fpn (Fig. 8A)

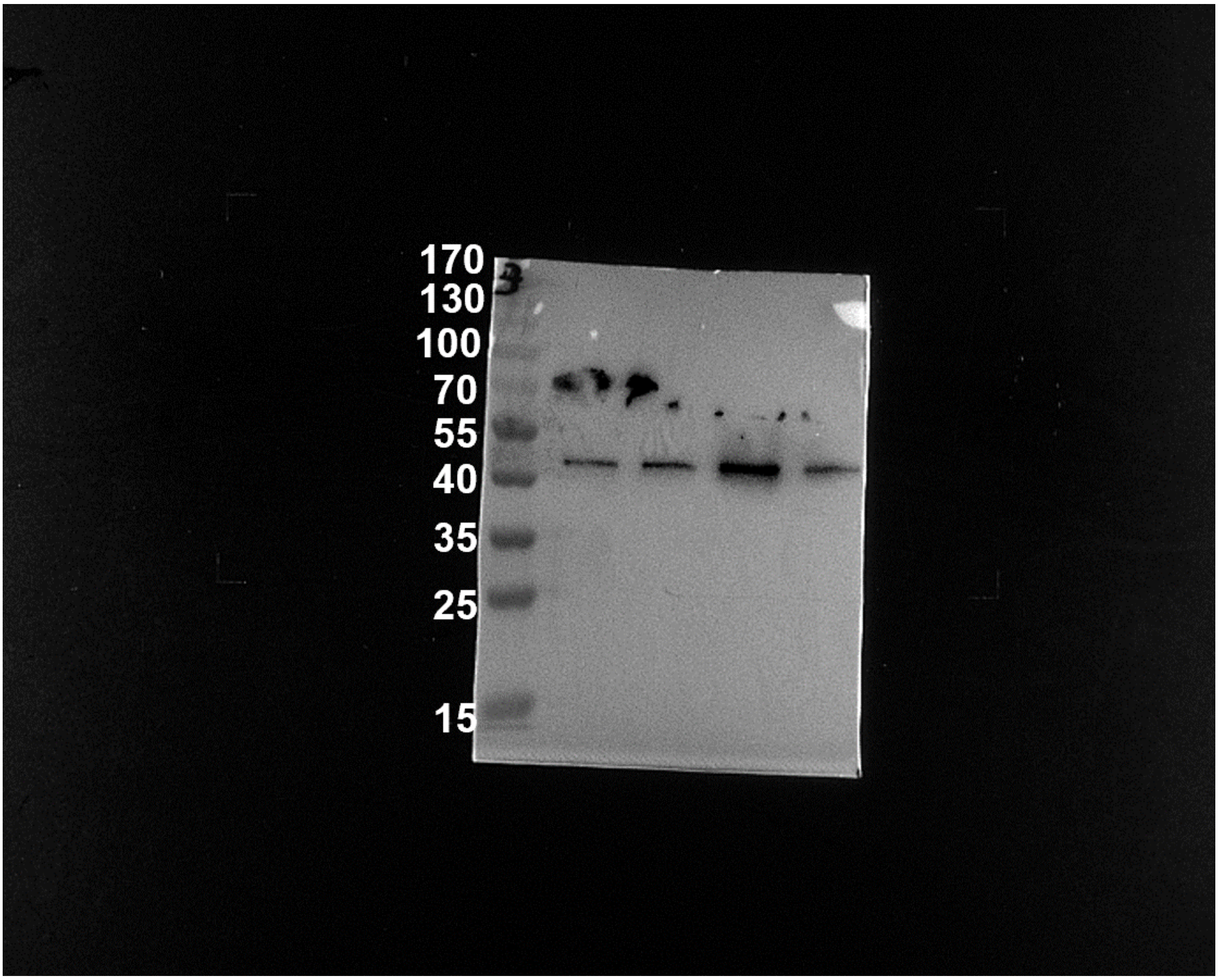

Hyp-PMC-GSHR-FtL (Fig. 8A)

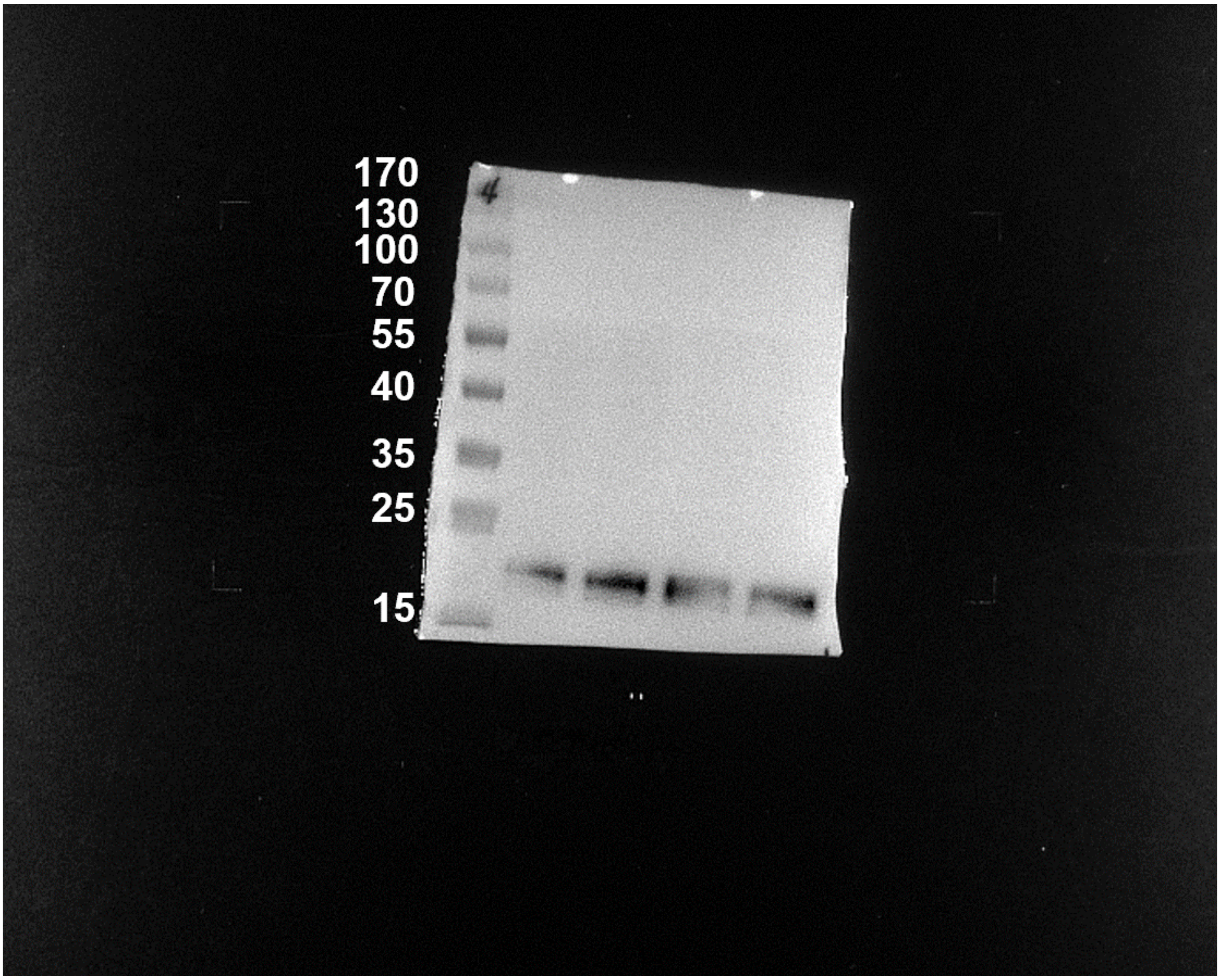

Hyp-PMC-GSHR-pErk (Fig. 8A)

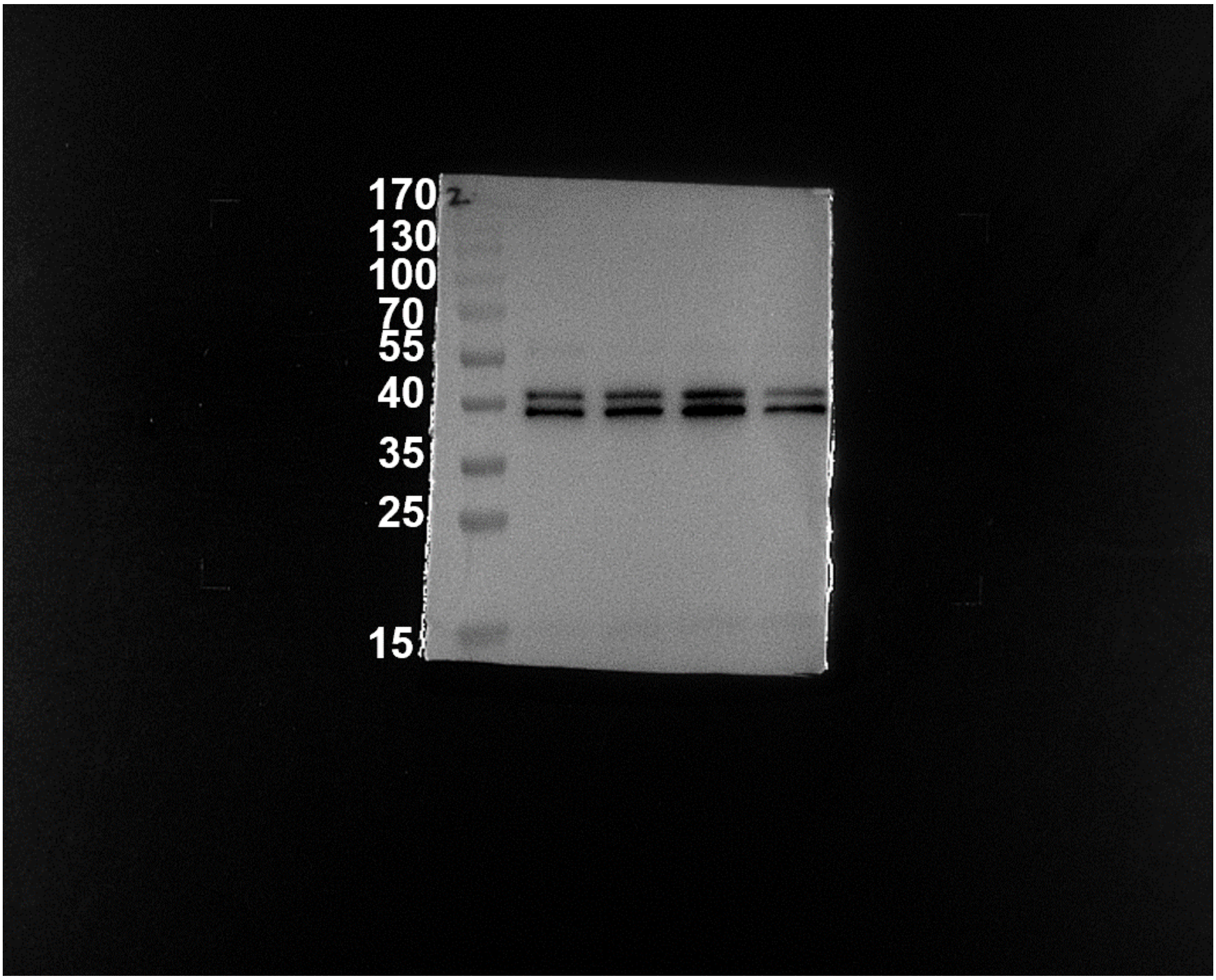

Hyp-PMC-GSHR-TfR (Fig. 8A)

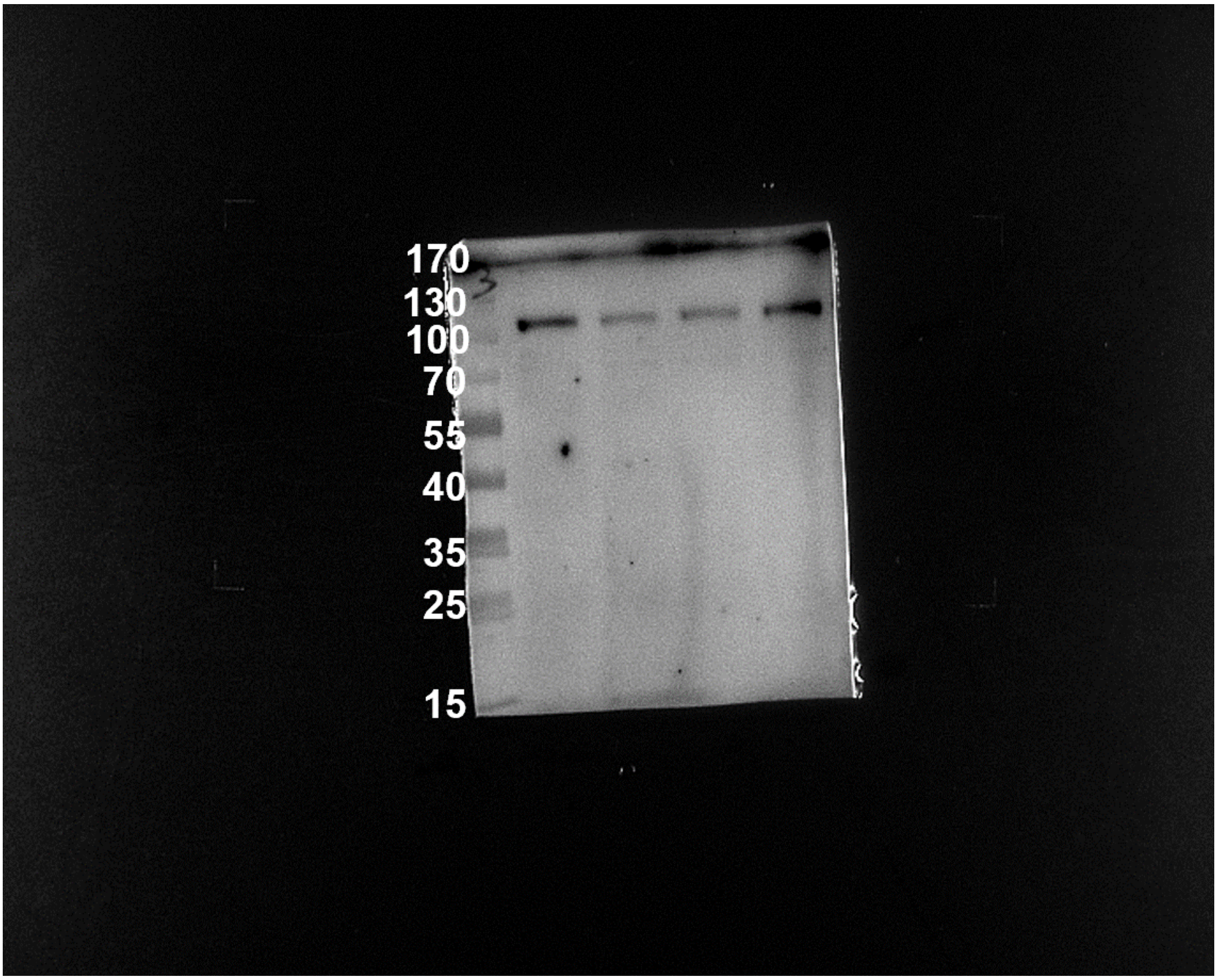

Hyp-PMC-U0126-actin (Fig. 8F)

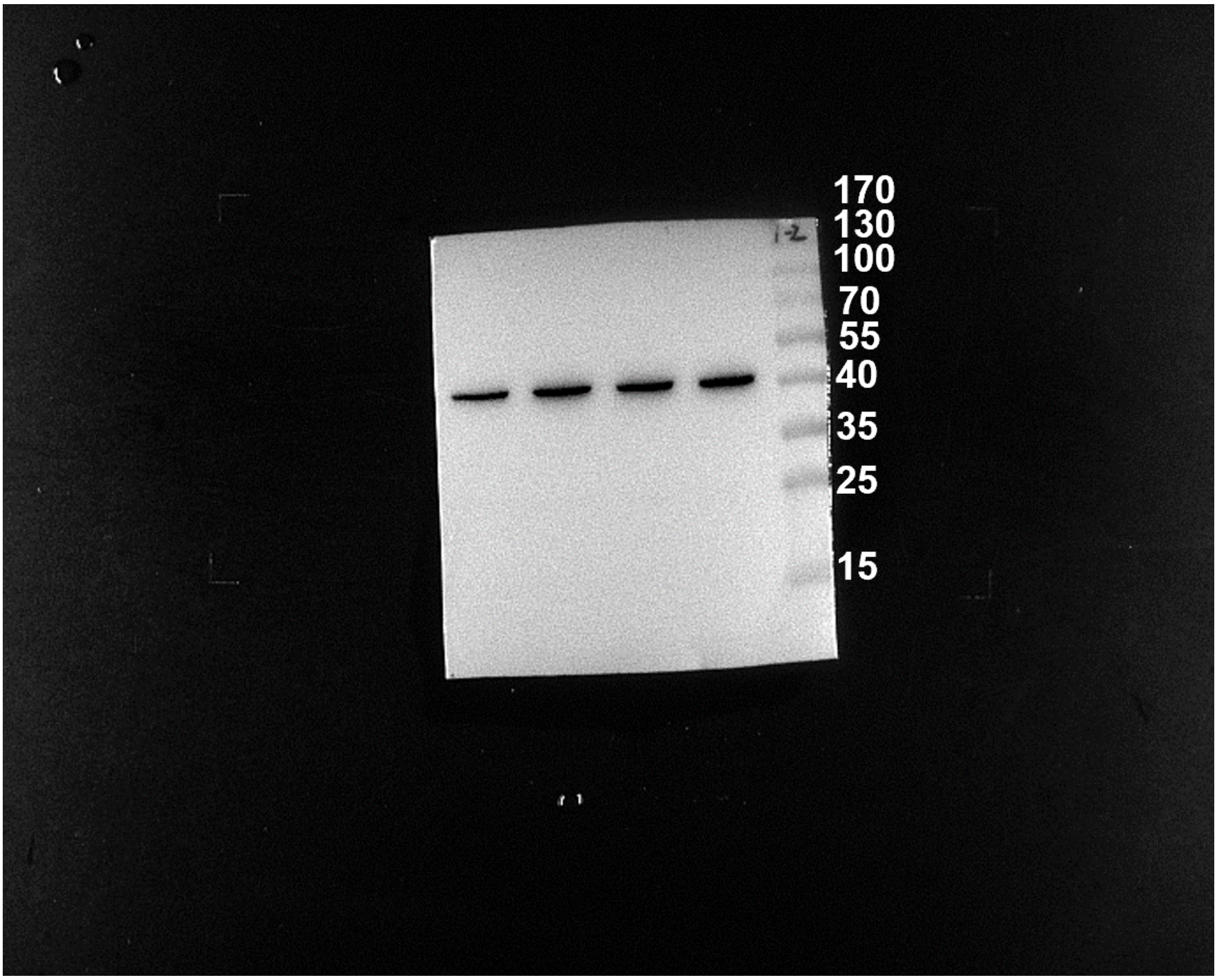

Hyp-PMC-U0126-Erk (Fig. 8F)

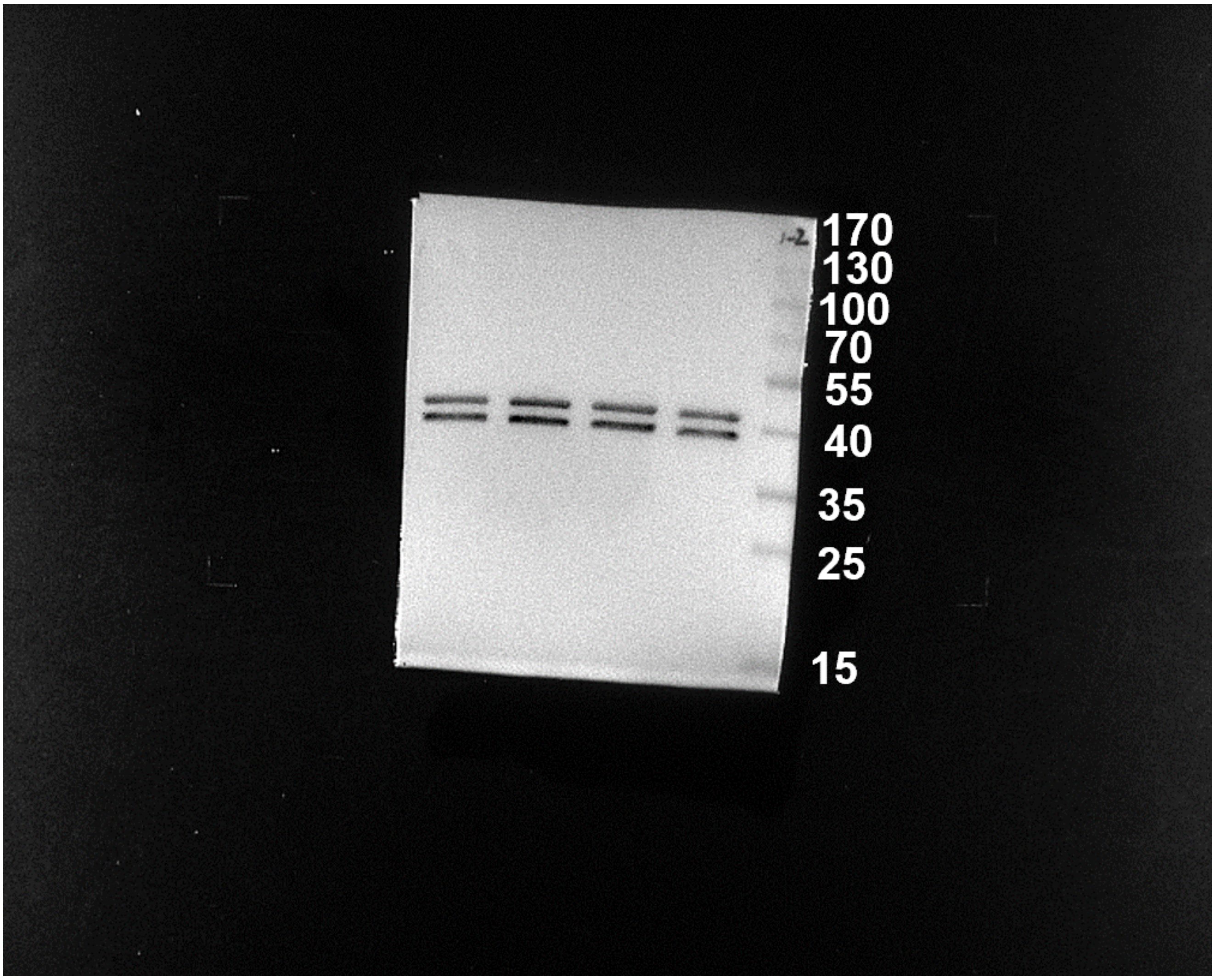

Hyp-PMC-U0126-Fpn (Fig. 8F)

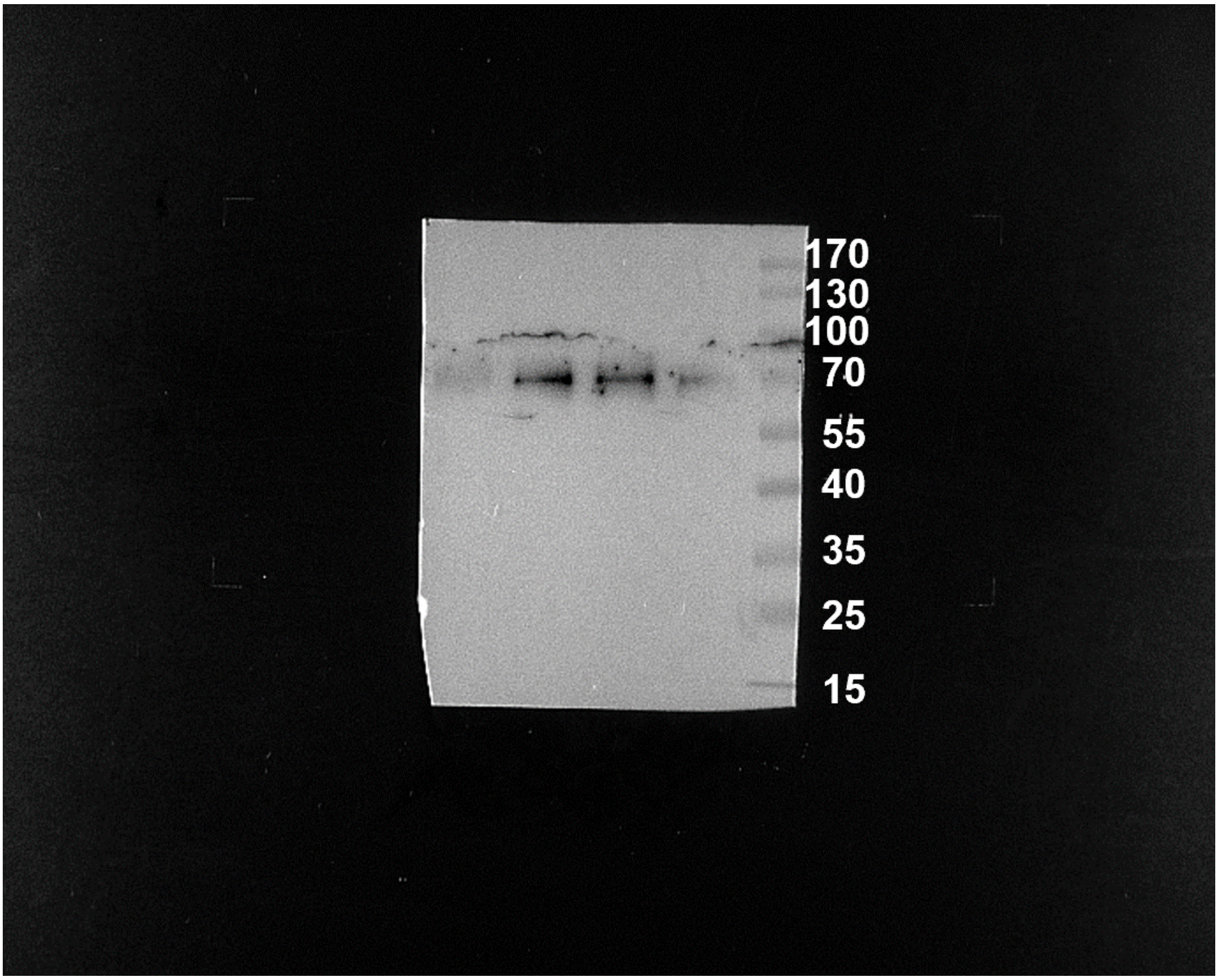

Hyp-PMC-U0126-FtL (Fig. 8F)

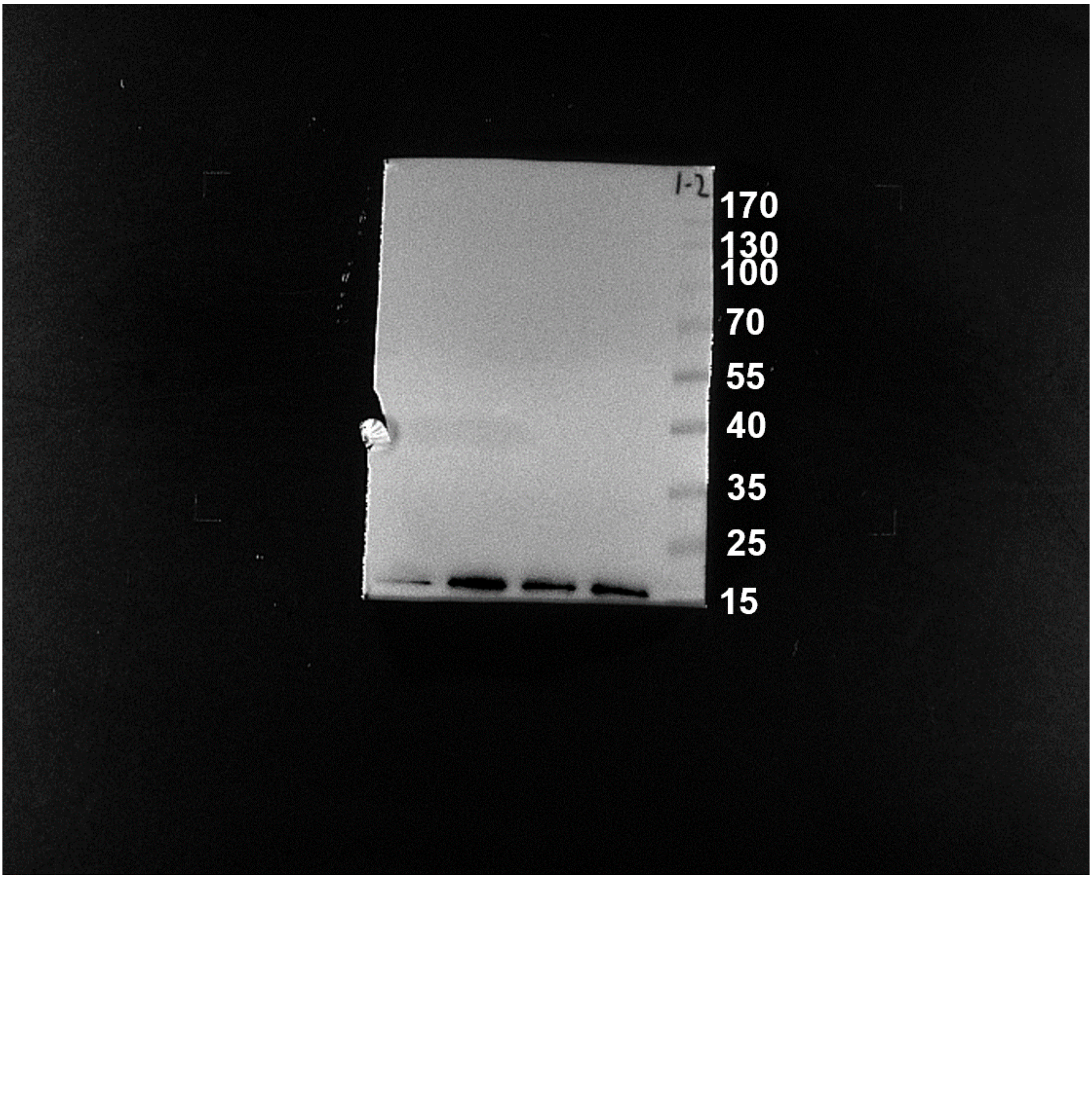

Hyp-PMC-U0126-pErk (Fig. 8F)

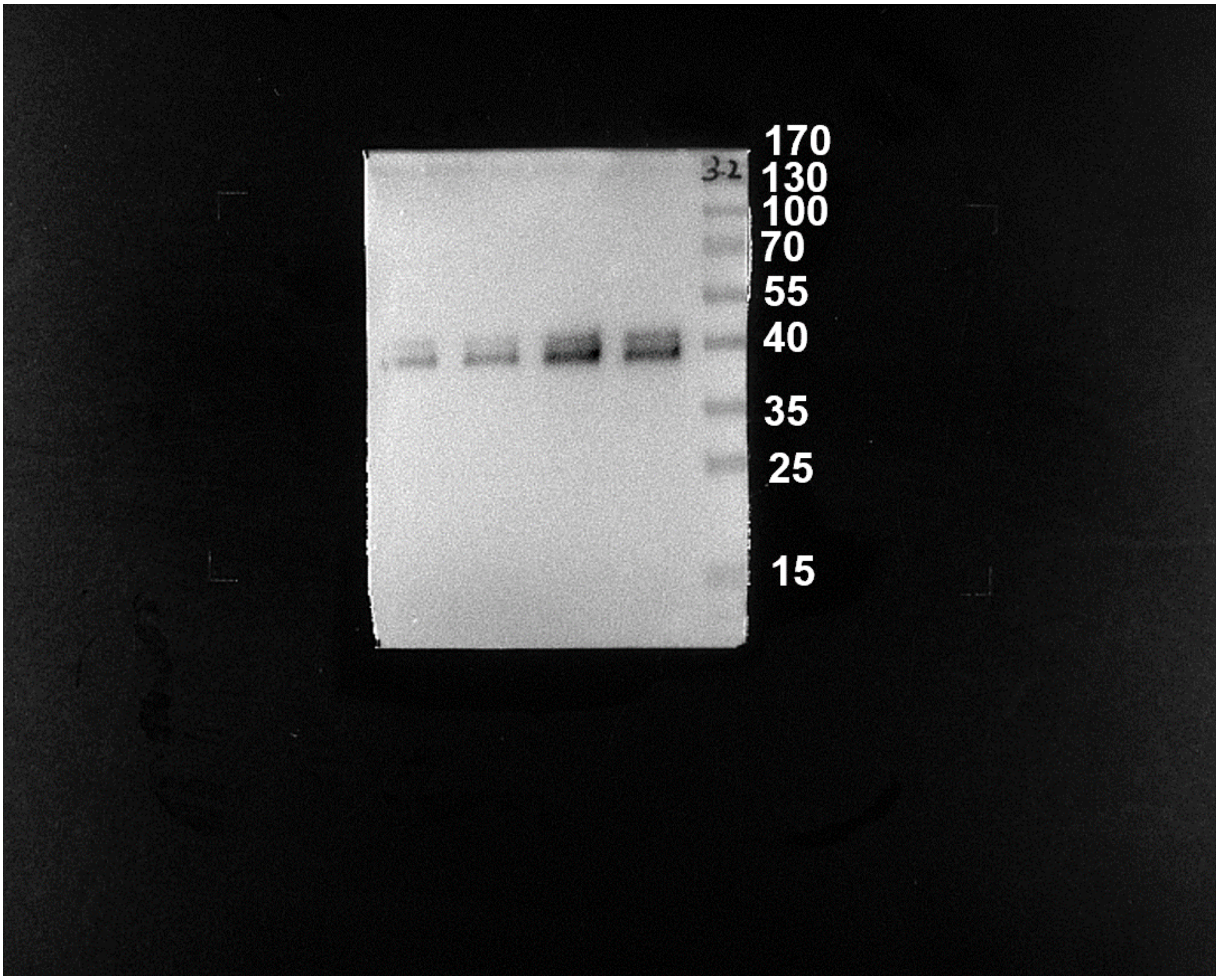

Hyp-PMC-U0126-TfR (Fig. 8F)

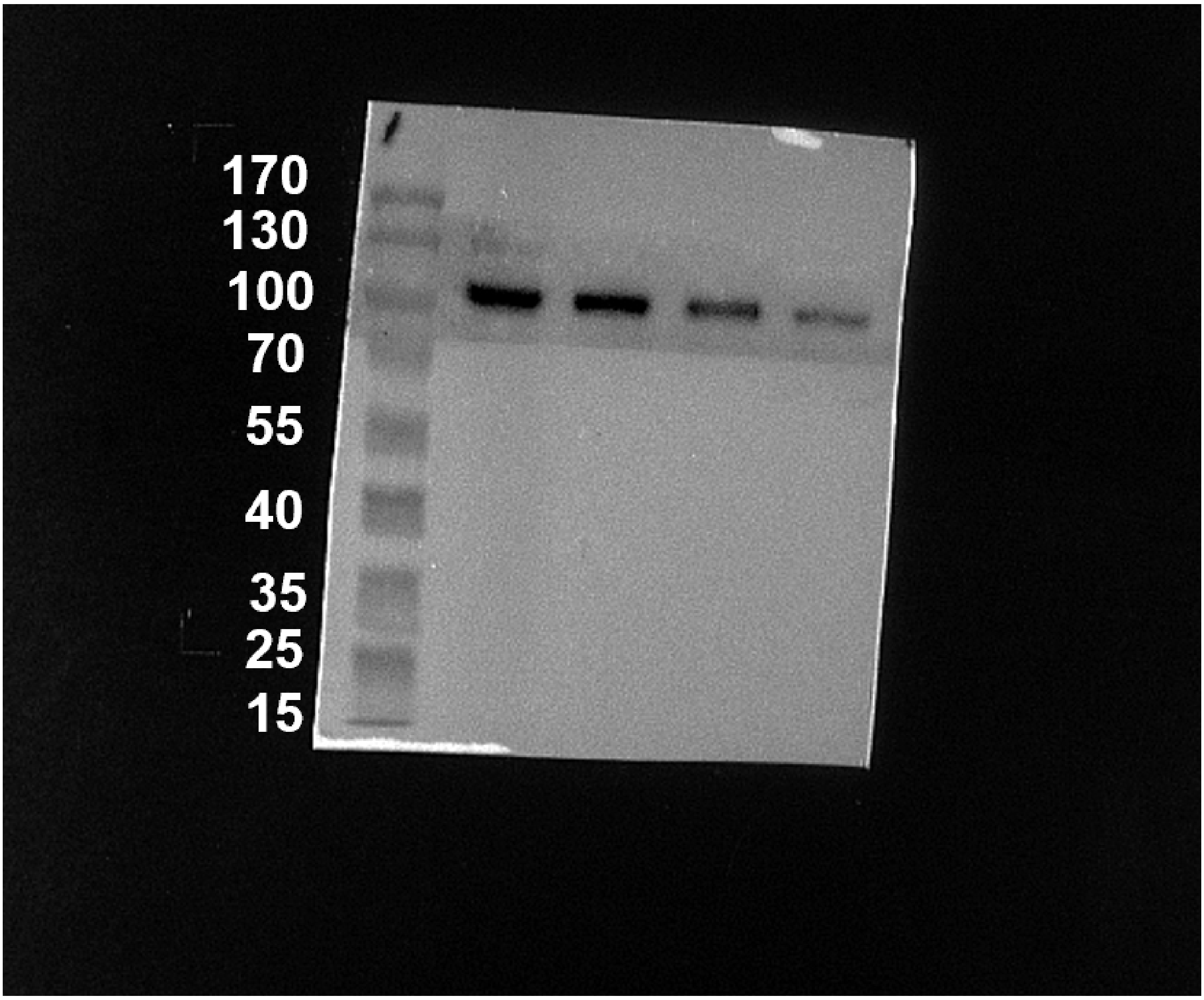

Figure 8A (left 4 lanes of each blot); Figure 8F (right 4 lanes of each blot)

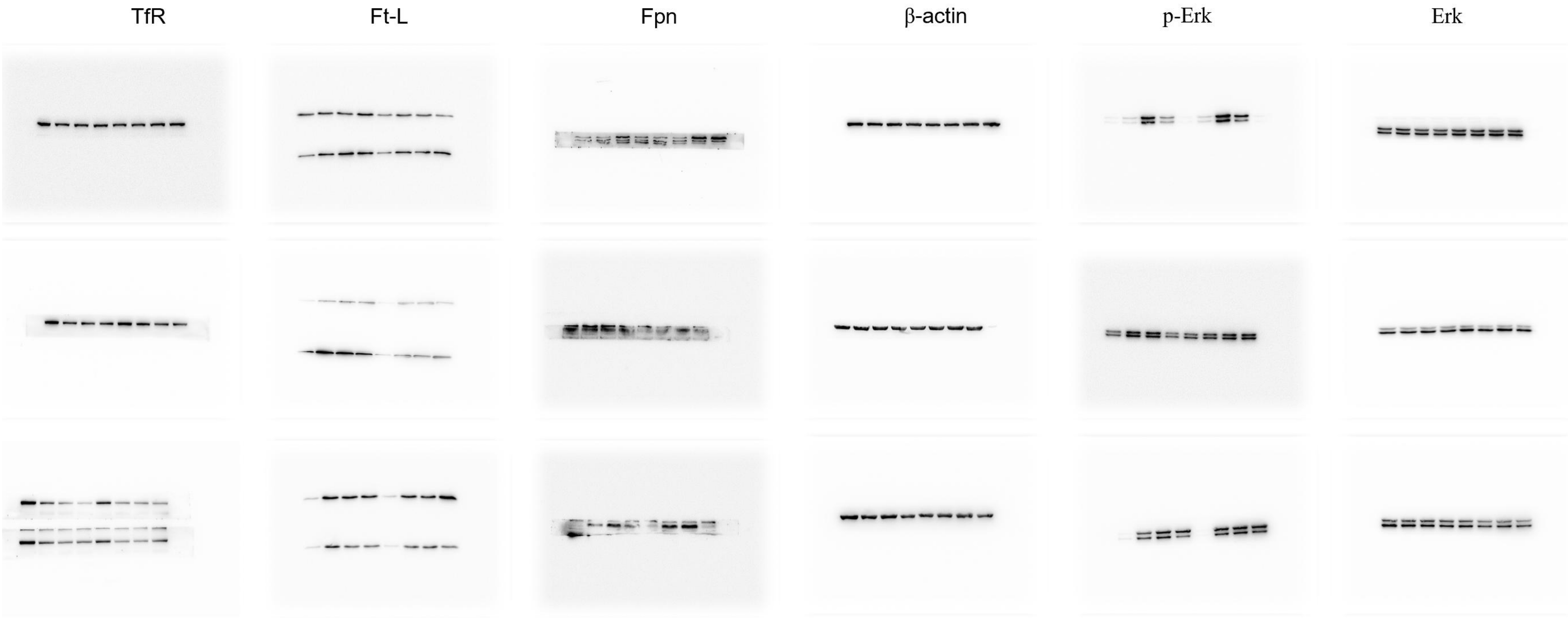

Supplement: Supplementary file 1 — Supplementary Information. [file 41598_2023_47596_MOESM1_ESM.pdf]
